# Trisulfide Bond‐Mediated Molecular Phototheranostic Platform for “Activatable” NIR‐II Imaging‐Guided Enhanced Gas/Chemo‐Hypothermal Photothermal Therapy

**DOI:** 10.1002/advs.202304104

**Published:** 2023-11-20

**Authors:** Gui‐long Wu, Fen Liu, Na Li, Qian Fu, Cheng‐kun Wang, Sha Yang, Hao Xiao, Li Tang, Feirong Wang, Wei Zhou, Wenjie Wang, Qiang Kang, Zelong Li, Nanyun Lin, Yinyin Wu, Guodong Chen, Xiaofeng Tan, Qinglai Yang

**Affiliations:** ^1^ Center for Molecular Imaging Probe Hunan Province Key Laboratory of Tumor Cellular and Molecular Pathology Cancer Research Institute Hengyang Medical School University of South China Hengyang Hunan 421001 China; ^2^ Department of Hepatopancreatobiliary Surgery The First Affiliated Hospital Hengyang Medical School University of South China Hengyang Hunan 421001 China; ^3^ National Health Commission Key Laboratory of Birth Defect Research and Prevention Hunan Provincial Maternal and Child Health Care Hospital Changsha Hunan 410008 China; ^4^ Key Laboratory of Tropical Medicinal Plant Chemistry of Ministry of Education College of Chemistry and Chemical Engineering Hainan Normal University Haikou Hainan 571158 China; ^5^ MOE Key Lab of Rare Pediatric Diseases Hengyang Medical School University of South China Hengyang Hunan 421001 China

**Keywords:** activatable fluorescence imaging, chemodynamic therapy, gas therapy, glutathione depletion, hypothermal photothermal therapy (HPTT), near‐infrared II, photoinduced electron transfer

## Abstract

Tumor microenvironment (TME)‐triggered phototheranostic platform offers a feasible strategy to improve cancer diagnosis accuracy and minimize treatment side effects. Developing a stable and biocompatible molecular phototheranostic platform for TME‐activated second near‐infrared (NIR‐II) fluorescence imaging‐guided multimodal cascade therapy is a promising strategy for creating desirable anticancer agents. Herein, a new NIR‐II fluorescence imaging‐guided activatable molecular phototheranostic platform (IR‐FEP‐RGD‐S‐S‐S‐Fc) is presented for actively targeted tumor imaging and hydrogen sulfide (H_2_S) gas‐enhanced chemodynamic‐hypothermal photothermal combined therapy (CDT/HPTT). It is revealed for the first time that the coupling distance between IR‐FE and ferrocene is proportional to the photoinduced electron transfer (PET), and the aqueous environment is favorable for PET generation. The part of Cyclic‐RGDfK (cRGDfk) peptides can target the tumor and benefit the endocytosis of nanoparticles. The high‐concentration glutathione (GSH) in the TME will separate the fluorescence molecule and ferrocene by the GSH‐sensitive trisulfide bond, realizing light‐up NIR‐II fluorescence imaging and a cascade of trimodal synergistic CDT/HPTT/gas therapy (GT). In addition, the accumulation of hydroxyl radicals (•OH) and down‐regulation of glutathione peroxidase 4 (GPX4) can produce excessive harmful lipid hydroperoxides, ultimately leading to ferroptosis.

## Graphical abstract

Trisulfide bond‐mediated molecular phototheranostic platform for “activatable” NIR‐II imaging‐guided enhanced gas‐chemo‐hypothermal photothermal therapy. A new phototheranostic platform is developed, utilizing cyclic‐RGDfk (cRGDfk) peptide for active tumor cell targeting and achieving glutathione (GSH)‐rich tumor environment photoinduced electron transfer (PET) blockade for fluorescence‐specific illumination and therapy.

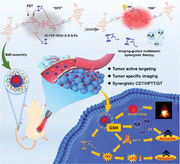

## 1 Introduction

Malignant tumors represent a significant and persistent global public health challenge, and effective treatment remains elusive.^[^ ^1^ ^]^ Conventional therapies such as surgery, chemotherapy, and radiation therapy are widely used clinically. However, their limited efficacy and significant drawback, including high recurrence rates, unpredictable side effects, and risk of secondary cancers, have prompted improved and innovative treatment approaches.^[^ ^2^ ^]^ A potential solution is to develop multifunctional phototheranostic platforms to diagnose structural abnormalities in neoplastic tissues and improve the therapeutic effect guided by imaging with deeper tissue penetration.^[^ ^3^ ^]^

The activatable therapeutic strategy could be triggered by the specific tumor microenvironment (TME), including reducibility, acidosis, hypoxia, and overexpressed hydrogen peroxide (H_2_O_2_) and glutathione （GSH）.^[^ ^4^ ^]^ These strategies yield precise diagnosis and therapeutic outcomes at the site of the tumor lesion.^[^ ^5^ ^]^ Compared with the conventional phototheranostics, most of which are in an “always‐on” mode, the activatable one could lighten fluorescence signals in specified lesions rather than normal tissues, thus obtaining a high tumor‐to‐normal tissue (T/N) signal ratio, sound sensitivity, and avoiding “false positive” signals. Moreover, the superior selectivity makes them overcome undesired toxic side effects during diagnosis and treatment.^[^ ^6^ ^]^ Therefore, developing activatable phototheranostic platforms would effectively improve the diagnosis specificity and therapeutic efficacy of the tumor. Recently, a series of TME‐responsive materials have been designed for antitumor research. For instance, Zhang et al. developed an intelligent nano‐factory AUC‐Gox/Cel that employs a tumor H_2_O_2_‐triggered strategy for homologous activated second near‐infrared (NIR‐II) fluorescence and chemodynamic therapy. This strategy significantly boosted H_2_O_2_ levels, achieving outstanding Self‐enhancing chemodynamic therapy (CDT) therapeutic efficacy.^[^ ^7^ ^]^ Zhao and Fan et al. developed an hydrogen sulfide (H2S)‐activatable nanostructured photothermal agent (Nano‐PT) for site‐specific NIR‐II fluorescence‐guided photothermal therapy（PTT） of colorectal cancer （CRC）. This study represents the first example of cancer biomarker‐mediated in situ conversion of synthetic molecules into NIR light‐responsive photothermal agents for NIR‐II fluorescence‐guided CRC therapy.^[^ ^8^ ^]^ Additionally, Zhao et al. reported the design of a tumor GSH‐activated NIR‐II phototheranostic nanoplatforms (Ag_2_S)‐Fe(III)‐DBZ Pdots, AFD NPs), which leverages the principle of Förster resonance energy transfer (FRET) to activate NIR‐II fluorescence and PTT by the overexpressed GSH in tumor tissues.^[^ ^9^ ^]^ These reports take advantage of the apparent strengths of NIR‐II fluorescence imaging, namely deep tissue penetration depth, low tissue scattering, and minimal background fluorescence interference.^[^ ^10^ ^]^ Moreover, the combination of NIR‐II imaging and multimodal therapy is an exact and efficient strategy for real‐time monitoring and assessment of treatment efficacy, thus further optimizing the overall safety and effectiveness of the therapeutic strategy.^[^ ^11^ ^]^ Nonetheless, developing TME‐activated NIR‐II optical imaging and therapeutic agents continues to pose a significant challenge. At present, the low sensitivity of the activated platform,^[^ ^12^ ^]^ the normal tissue damage from high‐extrinsic energy irradiation,^[^ ^13^ ^]^ and the low efficiency of monotherapies^[^ ^14^ ^]^ need to be addressed. At the same time, most TME‐responsive materials consist of inorganic metal and organic polymer. The potential toxicity caused by the release of metal ions strongly limits their translation toward clinical applications.^[^ ^14^, ^15^ ^]^ Hence, constructing a molecular phototheranostic platform with good stability and biocompatibility represents a rational proposal for developing TME‐activated NIR‐II fluorescence imaging‐guided multimodal cascade therapy.

Chemodynamic therapy (CDT) is an innovative cancer treatment strategy that exploits the Fenton and Fenton‐like reactions to selectively generate highly cytotoxic hydroxyl radicals (•OH) in tumor tissues with overexpressed H_2_O_2_ while minimizing damage to normal tissues.^[^ ^16^ ^]^ However, the complex and dynamic physiological environment of tumors, combined with their inherent self‐healing ability, present significant challenges to the effectiveness of CDT.^[^ ^17^ ^]^ To overcome these limitations, several approaches have been proposed to enhance the performance of CDT. One promising approach involves introducing exogenous energy fields, such as the photothermal effect, to elevate the temperature of the TME and stimulate the endogenous Fenton reaction, thereby increasing the production of •OH radicals.^[^ ^18^ ^]^ For example, Yang et al. constructed a CDT‐based nanoplatform by exploiting nanozymes with dual enzyme‐like activities, resulting in efficient therapy for cancer.^[^ ^19^ ^]^ Another strategy is introducing exogenous thioether bonding substances, such as disulfide bonds, to deplete glutathione (GSH) in the TME, thus preventing the consumption of •OH radicals and enhancing their availability for cancer cell destruction.^[^ ^20^ ^]^ Additionally, some reducing substances, such as H_2_S, can accelerate the conversion of Fe^3+^/Fe^2+^ and further promote •OH production, improving CDT's therapeutic efficacy.^[^ ^21^ ^]^ Photothermal therapy (PTT) achieves spatiotemporally controllable irradiation in tumor tissues while constrained by the low penetration ability and the risk of ambustion from the high local temperature.^[^ ^13^, ^22^ ^]^ Hypothermal photothermal therapy (HPTT) has attracted extensive attention by employing hyperthermia (<45 °C) to damage tumor cells with tiny side effects on the normal tissues.^[^ ^13^, ^23^ ^]^ However, the upregulated expression of heat shock proteins (HSPs) during the process of HPTT enhances the heat and drug tolerance of the cells. Consequently, significant worldwide interest has been devoted to exploring hypothermal PTT strategies that suppress the expression of HSPs.^[^ ^13^, ^24^ ^]^ Gas therapy (GT) is an emerging therapeutic strategy that exploits the bioeffects of various gases, such as hydrogen (H_2_),^[^ ^3a^ ^]^ hydrogen sulfide (H_2_S),^[^ ^25^ ^]^ and carbon monoxide (CO).^[^ ^26^ ^]^ The available gas at appropriate concentrations has an anticancer effect by reversing the Warburg effect in cancer cells and inhibiting the HSPs expression without adversely affecting the normal cells because of its endogeneity with various metabolic pathways. Thus, combining PTT with GT is expected to achieve an HPTT strategy with enhanced effectiveness against malignant tumors.^[^ ^23^, ^24^, ^26^, ^27^ ^]^ Consequently, TME‐activated NIR‐II fluorescence‐guided multiple treatment strategies could meet the requirements of precision medicine to combat malignant tumors.

**Scheme 1:**
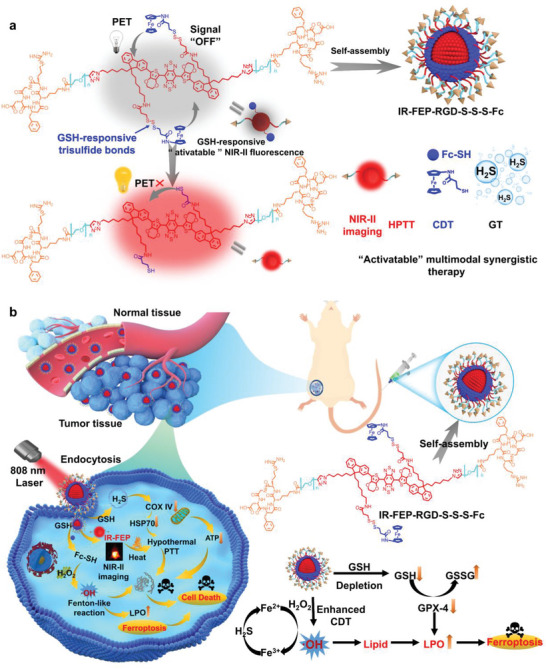
a) Proposed activation mechanism of the probe IR‐FEP‐RGD‐S‐S‐S‐Fc mediated by GSH. b) Mechanism of the NIR‐II fluorescent IR‐FEP‐RGD‐S‐S‐S‐Fc imaging‐guided synergistic CDT/HPTT/GT treatment.

Taking all these issues into account, a trisulfide bond‐mediated light‐up molecular phototheranostic platform (IR‐FEP‐RGD‐S‐S‐S‐Fc) is developed for GSH‐activatable NIR‐II imaging‐guided enhanced gas‐chemo‐photothermal synergistic therapy (**Scheme** **1**). The designed IR‐FEP‐RGD‐S‐S‐S‐Fc consists of two ferrocene units with a trisulfide linker, S‐D‐A‐D‐S type of NIR‐II fluorophore (IR‐FE), and two Cyclic‐RGDfK (cRGDfK) peptides (This peptide binds specifically to integrin αvβ3 (overexpressed on certain types of cancer cells^[^ ^28^ ^]^) provide active targeting ability to the tumor cells and benefit the endocytosis of nanoparticles). Significantly, the PET quenching mechanism of ferrocene molecules for S‐D‐A‐D‐S type molecules was verified for the first time, and the relationship between distance and quenching ability was explored. The magnitude of intramolecular quenching increased with the decreasing of spacer length between the fluorescent quencher (ferrocene) and core IR‐FEP (*λ*_ex_/*λ*_em_ = 808/1047 nm) and strengthened in water due to hydrophobicity pulling closer together. Interestingly, the PET quenching process could be removed in the presence of GSH owing to the release of ferrocene caused by cleavage of the trisulfide bond of IR‐FEP‐RGD‐S‐S‐S‐Fc in an aqueous solution, resulting in the recovery of fluorescence (Scheme 1a). It is worth noting that IR‐FEP‐RGD‐S‐S‐S‐Fc demonstrated a high tumor‐to‐normal tissue (T/N) ratio (up to 8.60 at 12 h) with low injection doses owing to its TME‐activated feature. In addition, the cleavage of sensitive trisulfide bond is accompanied by the release of H_2_S,^[^ ^29^ ^]^ which effectively suppress the catalase activity of mitochondrial cytochrome c oxidase (COX IV), thus leading to mitochondrial dysfunction and amplified oxidative stress. The lower expression of COX IV and mitochondrial dysfunction would further contribute to decreasing adenosine triphosphate (ATP) synthesis, thus blocking the expression of HSPs to improve the HPTT efficiency. Moreover, the accumulation of H_2_S could accelerate the conversion of Fe^3+^ to Fe^2+^, promoting the Fenton reaction cycle to generate more •OH. The exfoliated ferrocene could convert the endogenous hydrogen peroxide (H_2_O_2_) into plenty of reactive oxygen species (ROS) under laser irradiation.^[^ ^30^ ^]^ In the oxidative stress reaction, •OH would attack lipid molecules to produce more harmful lipid peroxides. The depletion of GSH cause the down‐regulation of glutathione peroxidase 4 (GPX4). The two aspects contribute to the accumulation of lipid hydroperoxides, ultimately leading to the ferroptosis process. Notably, this work employed low toxicity molecular phototheranostic platform with a laser power of 0.33 W cm^−2^ (the criteria of the Food and Drug Administration (FDA), 0.33 W cm^−2^ of 808 nm laser for skin exposure) for a cascade of trimodal synergistic therapy of CDT/HPTT/GT, which avoid the thermal diffusion caused by excessive hyperthermia that causes inflammation and collateral damage to the surrounding healthy tissues (Scheme 1b). Both in vitro and in vivo results showed that IR‐FEP‐RGD‐S‐S‐S‐Fc is a promising agent for activated imaging‐guided precise synergistic therapy in living systems. This work presents a distinct paradigm for developing the NIR‐II molecular phototheranostic platforms for imaging‐guided anticancer treatments of enhanced CDT/HPTT/GT.

## 2 Results and Discussion

### 2.1 Design of IR‐FEP‐RGD‐S‐S‐S‐Fc

In this study, we developed a NIR‐II fluorescence imaging‐guided organic molecular phototherapy platform (IR‐FEP‐RGD‐S‐S‐S‐Fc). The amphiphilic IR‐FEP‐RGD‐S‐S‐S‐Fc could be self‐assembled into stable nanoparticles and decomposed by the high concentration of GSH. The part of cRGDfk peptides provides active targeting ability to the tumor cells and benefits the endocytosis of nanoparticles. After decomposition by the highly expressive GSH in the tumor cell, the trisulfide bond with good GSH sensitivity is cleaved to activate NIR‐II fluorescence from an extremely robust PET process in an aqueous environment. Simultaneously, this process would generate H_2_S gas to reduce enzyme activity, thus lowering ATP production. Moreover, H_2_S gas therapy would downregulate heat shock protein 70 (HSP70), lowering tumor heat resistance and enhancing HPTT therapeutic effect. The part of IR‐FEP‐RGD generates hyperthermia under NIR laser (808 nm) irradiation for thermal ablation of cancer cells. The unbound ferrocene could convert the endogenous hydrogen peroxide (H_2_O_2_) into hydroxyl radical •OH to kill tumor cells. Additionally, lipid peroxidation caused by ferrocene metabolism and devitalization of Glutathione Peroxidase 4 (GPx4) triggers tumor ferroptosis, further enhancing the final tumor therapeutic effect.

### 2.2 PET Fluorescence Quenching of IR‐FEP‐RGD‐S‐S‐S‐Fc

**Figure 1:**
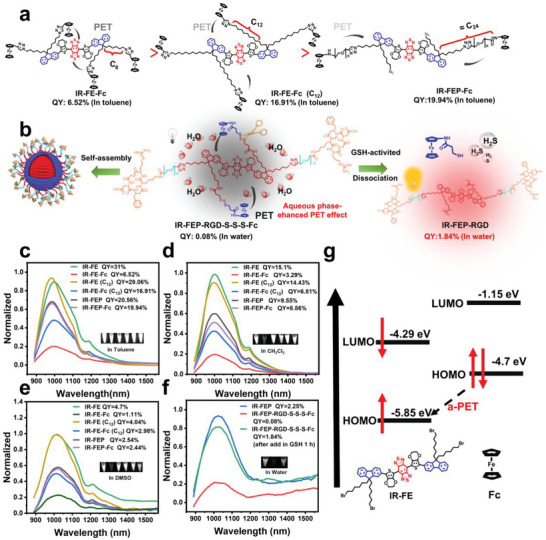
a) The PET quenching mechanism of ferrocene molecules for S‐D‐A‐D‐S type molecules and the relationship between distance and quenching. b) The self‐assembly under the action of hydrophobic interaction in the aqueous phase system enhanced the PET effect of IR‐FEP‐RGD‐S‐S‐S‐Fc. c) Fluorescence emission spectra of the molecular fluorophores in toluene solutions. d) Fluorescence emission spectra of the molecular fluorophores in Dichloromethane (DCM) solutions. e) Fluorescence emission spectra of the molecular fluorophores in DMSO solutions. The emission spectra in (c), (d), and (e) were measured with an optical density (OD) of 0.08 (*λ*_ex_: 808 nm laser, QY data were quantified with IR‐FE as a reference^[^ ^32^ ^]^). Fluorescence was normalized with IR‐FE peak intensity. Inset: corresponding NIR‐II fluorescence images. f) Fluorescence emission spectra of the PEGylated fluorophores in aqueous solutions. The emission spectra in (f) were measured with an OD of 0.08 (*λ*_ex_: 808 nm laser, QY data were quantified with IR‐FEP as a reference^[^ ^32^ ^]^). Fluorescence normalized with IR‐FEP peak intensity. g) The calculated HOMO/LUMO levels of IR‐FE and Fc.

In our previous report^[^ ^31^ ^]^ ferrocene molecules could partially quench the fluorescence of IR‐FE‐Fc and decrease its quantum yields (QYs) owing to the PET quenching mechanism. Furthermore, a high laser power of 1.0 W cm^−2^ was employed to excite combined PTT/CDT to achieve the desired treatment effect. Herein, a GSH activatable NIR‐II fluorescence molecule was designed by exploiting the PET quenching mechanism between the fluorophores and ferrocene. The structure‐quenching relationship between ferrocene molecules and S‐D‐A‐D‐S type molecules was first systematically investigated. Three different chain lengths of S‐D‐A‐D‐S type of NIR‐II fluorophores, IR‐FE‐Fc, IR‐FE‐Fc (C_12_), and IR‐FEP‐Fc (the distance between the fluorescent core and the quencher ferrocene is 6, 12, and 24 carbons approximately), were synthesized according to the previous references.^[^ ^32^ ^]^ (Schemes S1–S3, Supporting Information). As a control, IR‐FEP was produced by a stacking‐alkyl click cyclization reaction between IR‐FE and Alkyne‐PEG (Scheme S4, Supporting Information). These compounds were characterized by nuclear magnetic resonance (NMR) and high‐resolution resolution mass spectrometry (HRMS) (Figures S1–S16, Supporting Information). The PET quenching mechanism of ferrocene molecules for S‐D‐A‐D‐S type of NIR‐II fluorophores was verified for the first time, and the relationship between distance and quenching ability was explored. Alkyl chains of different lengths have been examined as spacers to investigate the effect of spacer length on PET efficiency, and the impact of molecular distance on the quenching mechanism was concluded. These results demonstrated that the magnitude of intramolecular quenching by the PET process increased with the decreasing of spacer length between the fluorescent quencher (ferrocene molecules) and an organic NIR‐II fluorescence IR‐FEP (*λ*_ex_/*λ*_em_ = 808/1047 nm) (**Figure** **1**a). Organic quenched fluorophores IR‐FEP‐RGD‐S‐S‐S‐Fc synthesized based on the PET mechanism tend to self‐assemble in an aqueous solution due to hydrophobicity pulling closer together and enhanced PET (Figure 1b).

As shown in Figure 1c–e, the measured fluorescence QYs of compounds IR‐FEP‐Fc (19.94%) and IR‐FE‐Fc (C_12_) (16.91%) experienced a 0.03% and 41.8% quenching of fluorescence compared to compounds IR‐FEP (20.56%) and IR‐FE (C_12_) (29.06%) in toluene. Meanwhile, the QY of compound IR‐FE‐Fc (6.52%) experienced a substantially more significant quenching effect of 79% compared to compound IR‐FE (31%) in toluene. Similar results were confirmed by testing in the dichloromethane and dimethyl sulfoxide (DMSO). These results demonstrated that the effectiveness of the quenching event decreases as the fluorescent quencher (ferrocene) becomes more distal. IR‐FE and Fc act as electron acceptors and donors during the a‐PET quenching process to estimate the orbital levels. As shown in Figure 1g, the highest occupied molecular orbital (HOMO) energy of Fc is ≈ −4.7 eV,^[^ ^33^ ^]^ which is between the HOMO (−5.85 eV) and lowest occupied molecular orbital (LUMO) (−4.29 eV) energy of IR‐FE.^[^ ^10b^ ^]^ Therefore, electron transfer could happen from the acceptor (Fc) into the fluorophore to cause fluorescence quenching because the HOMO energy level of the receptor is higher than that of the fluorophore.^[^ ^34^ ^]^ Notably, the self‐quenching of the IR‐FEP‐RGD‐S‐S‐S‐Fc based on the PET mechanism could be intensively achieved in the aqueous environment. As shown in Figure 1f, compared to IR‐FEP (measured fluorescence QY: 2.25%), the measured fluorescence QY of compounds IR‐FEP‐RGD‐S‐S‐S‐Fc (measured fluorescence QY:0.08%) experienced a 96.4% fluorescence quenching. These results aligned with several reports that self‐assembly under the action of hydrophobic interaction in the aqueous phase system can bring the distance closer and enhance the PET effect.^[^ ^35^ ^]^ Moreover, fluorescence QY of IR‐FEP‐RGD‐S‐S‐S‐Fc obtains a remarkable fluorescence enhancement (≈23 fold, QY is 1.84%) after GSH incubation for 1 h, which lays a foundation for the GSH‐activated molecular therapeutical platform. IR‐FEP‐RGD‐S‐S‐S‐Fc was prepared by amidation reactions between IR‐FEP‐RGD and Fc‐S‐S‐S‐COOH. The precursors and final product (3,3′‐trisulfanediyldipropionic acid, Fc‐S‐S‐S‐COOH, and IR‐FEP‐RGD‐S‐S‐S‐Fc) were all characterized and confirmed with NMR, HRMS and size‐exclusion chromatography (SEC) (Schemes S5 and S6, Supporting Information) (Figures S17–S30, Supporting Information).

### 2.3 Characterization of IR‐FEP‐RGD‐S‐S‐S‐Fc

In view of the excellent GSH‐activated fluorescence feature of IR‐FEP‐RGD‐S‐S‐S‐Fc, its physicochemical properties were further investigated. IR‐FEP, IR‐FEP‐RGD, IR‐FEP‐Fc and

**Figure 2:**
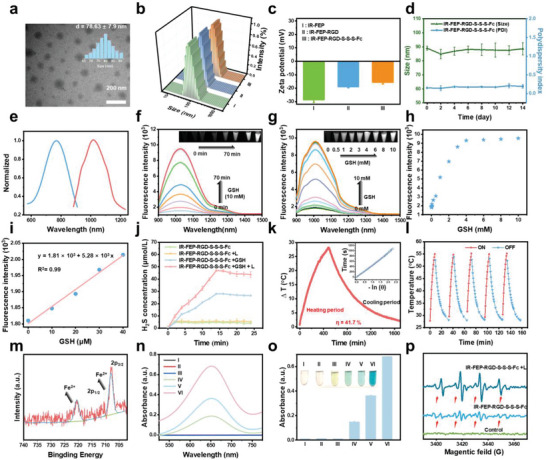
Characterization and physicochemical properties. a) TEM images of IR‐FEP‐RGD‐S‐S‐S‐Fc. b) Hydrodynamic diameter of (I: IR‐FEP, II: IR‐FEP‐RGD, III: IR‐FEP‐RGD‐S‐S‐S‐Fc) measured by the dynamic light scattering system. c) *ζ* potential of IR‐FEP, IR‐FEP‐RGD, and IR‐FEP‐RGD‐S‐S‐S‐Fc. d) Size stability of IR‐FEP‐RGD‐S‐S‐S‐Fc in PBS for 14 days. e) Normalized absorption and emission spectra of IR‐FEP‐RGD‐S‐S‐S‐Fc in aqueous solution (*λ*_ex_: 808 nm laser). f) Temporal gradient fluorescence spectra of activable probe IR‐FEP‐RGD‐S‐S‐S‐Fc (50 µm, *λ*_ex_: 808 nm laser) in the presence of GSH (10.0 mm) in aqueous solution. Inset: corresponding NIR‐II fluorescence images of IR‐FEP‐RGD‐S‐S‐S‐Fc. g) Fluorescence spectra of IR‐FEP‐RGD‐S‐S‐S‐Fc (50 µm, *λ*_ex_: 808 nm laser) in aqueous solution with the gradual addition of GSH concentration (0–10.0 mm). h) Fluorescence intensity variation of IR‐FEP‐RGD‐S‐S‐S‐Fc at different concentrations of GSH (0–10.0 mm). i) The linear plot of the fluorescence intensity of IR‐FEP‐RGD‐S‐S‐S‐Fc with the concentrations of GSH over 0–40 µm. j) H_2_S release curves of IR‐FEP‐RGD‐S‐S‐S‐Fc detected by WSP‐1 under different conditions. k) PCE curve of IR‐FEP‐RGD‐S‐S‐S‐Fc (50 µm, *λ*_ex_: 808 nm laser, 1.0 W cm^−2^). l) Five “on−off” cycles of IR‐FEP‐RGD‐S‐S‐S‐Fc (*λ*_ex_: 808 nm laser, 1.0 W cm^−2^). m) XPS spectra of IR‐FEP‐RGD‐S‐S‐S‐Fc powder. n) The absorption spectra of TMB aqueous solution with different treatments (I: TMB − L, II: TMB + H_2_O_2_ − L, III: TMB + H_2_O_2_ + IR‐FEP‐RGD − L, IV: TMB + H_2_O_2_ + IR‐FEP‐RGD‐S‐S‐S‐Fc − L, V: TMB + H_2_O_2_ + IR‐FEP‐RGD‐S‐S‐S‐Fc + GSH, VI: TMB + H_2_O_2_ + IR‐FEP‐RGD‐S‐S‐S‐Fc +GSH + L, + L and − L are present as 808 nm laser/non‐laser irradiation). o) A652 TMB oxidation intensity of Figure 2n, Inset: images of relevant color variations). p) ESR spectra of H_2_O_2_ processed with different treatments: Control (DMPO) − L, IR‐FEP‐RGD‐S‐S‐S‐Fc − L, GSH + IR‐FEP‐RGD‐S‐S‐S‐Fc − L, GSH + IR‐FEP‐RGD‐S‐S‐S‐Fc + L. Data were presented as mean ± SD (*n* = 4).

IR‐FEP‐RGD‐Fc were also prepared as a control group. The SEC traces and related data of IR‐FEP, IR‐FEP‐RGD, IR‐FEP‐Fc, and IR‐FEP‐RGD‐S‐S‐S‐Fc are shown in Figure S30 and Table S1 (Supporting Information). The retention time decreased after being coupled with different moieties. The calculated *M*_w_ differences in IR‐FEP (4000), IR‐FEP‐Fc (4500), IR‐FEP‐RGD (4800), and IR‐FEP‐RGD‐S‐S‐S‐Fc (5600) indicated that different moieties were stably coupled. Transmission electron microscopy (TEM) and dynamic light scattering (DLS) were performed to analyze the morphology and size distribution of the samples. IR‐FEP‐RGD‐S‐S‐S‐Fc demonstrated a spherical shape with an average diameter of ≈78 nm (**Figure** **2**a), which aligns with the DLS findings. (Figure 2b). IR‐FEP presented a hydrodynamic diameter of 74.2 ± 14 nm and a ζ potential of −28.6 ± 2.2 mV. The IR‐FEP‐RGD sizes did not change significantly, while the modification of positively charged cRGDfk peptide changed its zeta potential to −18.9 ± 0.8 mV. After being coupled with Fc‐S‐S‐S‐COOH, the hydrodynamic diameter of IR‐FEP‐RGD‐S‐S‐S‐Fc increased to 85.9 ± 16.2 nm, and its zeta potential changed from −18.9 ± 0.8 to −15.6 ± 1.25 mV owing to the conjugation of the Fc (Figure 2c).^[^ ^36^ ^]^ Figure 2d shows the mean hydrodynamic diameter and polydispersity index (PDI) of IR‐FEP‐RGD‐S‐S‐S‐Fc as a function of time. Within 14 days, the size and PDI almost have no fluctuation in PBS. The optical spectra of IR‐FEP‐RGD‐S‐S‐S‐Fc displayed a broad absorption peak at 770 nm and emission at 1030 nm (In GSH 10 mm environment), which verified that the IR‐FEP‐RGD‐S‐S‐S‐Fc has potential for bioimaging (Figure 2e). The fluorescence activation performance of IR‐FEP‐RGD‐S‐S‐S‐Fc by GSH was investigated. The results indicated that the fluorescence of IR‐FEP‐RGD‐S‐S‐S‐Fc gradually recovered by GSH (10 mm) within 70 min (Figure 2f). Furthermore, the fluorescence intensity of IR‐FEP‐RGD‐S‐S‐S‐Fc increased with the addition of GSH incubation concentration and finally plateaued at 4 mm. (Figure 2g,h). Notably, benefiting from the sensitivity of trisulfide bonds to GSH, the fluorescent intensity at 1030 nm presented

a line correlation with GSH concentration within 0–40 µm (Figure 2i), which could be used for the quantitative detection of GSH. Based on the fitted curve, the limit of detection (LOD) was calculated to be 0.053 µm (KS_b_/m, where K is 3, S_B_ is the standard deviation of the blank sample, and m is the slope of the linear regression equation), surpassing some other reported similar sensing platforms (Table S2, Supporting Information).^[^ ^37^ ^]^

The selectivity of IR‐FEP‐RGD‐S‐S‐S‐Fc in response to GSH is the most significant element for GSH‐responsive applications in complex bioenvironments. The effect of other biological interference matter on IR‐FEP‐RGD‐S‐S‐S‐Fc, such as amino acids and abundant metal ions, was investigated to assess the potential of IR‐FEP‐RGD‐S‐S‐S‐Fc in natural biological systems. IR‐FEP‐RGD‐S‐S‐S‐Fc exhibited a significant fluorescence in GSH while no response to a wide range of amino acids and metal ions (Ca^2+^, Na^+^, Mg^2+^), demonstrating the excellent selectivity to GSH for IR‐FEP‐RGD‐S‐S‐S‐Fc in a biological system (Figure S31, Supporting Information). The highly selective fluorescence response to GSH in complicated physiological environments presents enormous potential for specific tumor imaging.

Additionally, the GSH‐triggered trisulfide bond breakage in IR‐FEP‐RGD‐S‐S‐S‐Fc was validated via HRMS (Figure S32, Supporting Information). The fragment of Fc‐SH as the intermediate was found after incubation of IR‐FEP‐RGD‐S‐S‐S‐Fc and GSH. Accompanied by the cleavage of trisulfide bonds by GSH, IR‐FEP‐RGD‐S‐S‐S‐Fc could serve as potential H_2_S donors.^[^ ^29^ ^]^ A classic probe, Washington State Probe 1 (WSP‐1), was used to confirm the production of H_2_S.^[^ ^38^ ^]^ The results exhibited a time‐dependent H_2_S release after GSH incubation. The laser irradiation could enhance the H_2_S release rate (Figure 2j). Significantly, the signal becomes more pronounced constantly upon adding IR‐FEP‐RGD‐S‐S‐S‐Fc with GSH under laser irradiation. As we know, a sustained supply of H_2_S (>1 nm) could inhibit cancer cells.^[^ ^26^ ^]^ Thus, IR‐FEP‐RGD‐S‐S‐S‐Fc was expected to be a superior candidate for cancer gas therapy.

The photothermal performance of IR‐FEP‐RGD‐S‐S‐S‐Fc was further evaluated. The results indicated that IR‐FEP‐RGD‐S‐S‐S‐Fc exhibited concentration‐depended and power density‐depended photothermal properties under 808 nm irradiation (Figure S33, Supporting Information). According to calculations, IR‐FEP‐RGD‐S‐S‐S‐Fc showed a photothermal conversion efficiency (PCE) of 41.7%. (Figure 2k), which is comparable compared with that of several Fe‐based nanoplatforms for combined photothermal therapy (PTT)/CDT (PCE is generally between 22.62% and 55.86%). The photothermal stability of IR‐FEP‐RGD‐S‐S‐S‐Fc was confirmed by the laser on‐off cycle test with almost no change after five cycles (Figure 2l), demonstrating significant photothermal stability under NIR irritation.

The X‐ray photoelectron spectroscopy (XPS) analysis showed that the characteristic peaks in 720.18 and 707.68 eV were assigned to 2p^1^/2 and 2p^3^/2 of Fe^2+^ (Figure 2m), confirming the excellent stability of IR‐FEP‐RGD‐S‐S‐S‐Fc. Hydroxyl radical (•OH) during the Fenton reaction was first detected by the 3,3′,5,5′‐tetramethyl‐benzidine (TMB) probe.^[^ ^39^ ^]^ As shown in Figure 2n, the generation of •OH was confirmed by the absorption peak at 652 nm obtained in the group of IR‐FEP‐RGD‐S‐S‐S‐Fc + H_2_O_2_. Moreover, the absorption peak increased after the addition of GSH due to the promoted Fenton reaction by the yielded H_2_S. Remarkably, the peak intensity at 652 nm was further increased when IR‐FEP‐RGD‐S‐S‐S‐Fc + H_2_O_2_ was exposed to laser irradiation, meaning laser irradiation can boost the generation of •OH (Figure 2n,o).

Meanwhile, a similar hydroxyl radical test experiment was conducted using a TMB probe after five laser on‐off cycles of laser (808 nm) irradiation of IR‐FEP‐RGD‐S‐S‐S‐Fc (Figure S34, Supporting Information). The result showed similar to those without laser irradiation (Figure 2n,o), which proved the excellent laser catalytic stability of IR‐FEP‐RGD‐S‐S‐S‐Fc. In addition, electron spin resonance (ESR) spectra of IR‐FEP‐RGD‐S‐S‐S‐Fc + H_2_O_2_ through detecting 5, 5‐dimethyl‐1‐pyrroline N‐oxide (DMPO)‐•OH spin adduct exhibited typical signal peaks, and its signal intensity was also enhanced under the laser irradiation (Figure 2p), meaning higher generation efficiency of •OH.

### 2.4 Assessment of Tumor Targeting Ability and Intracellular Active Substances

**Figure 3:**
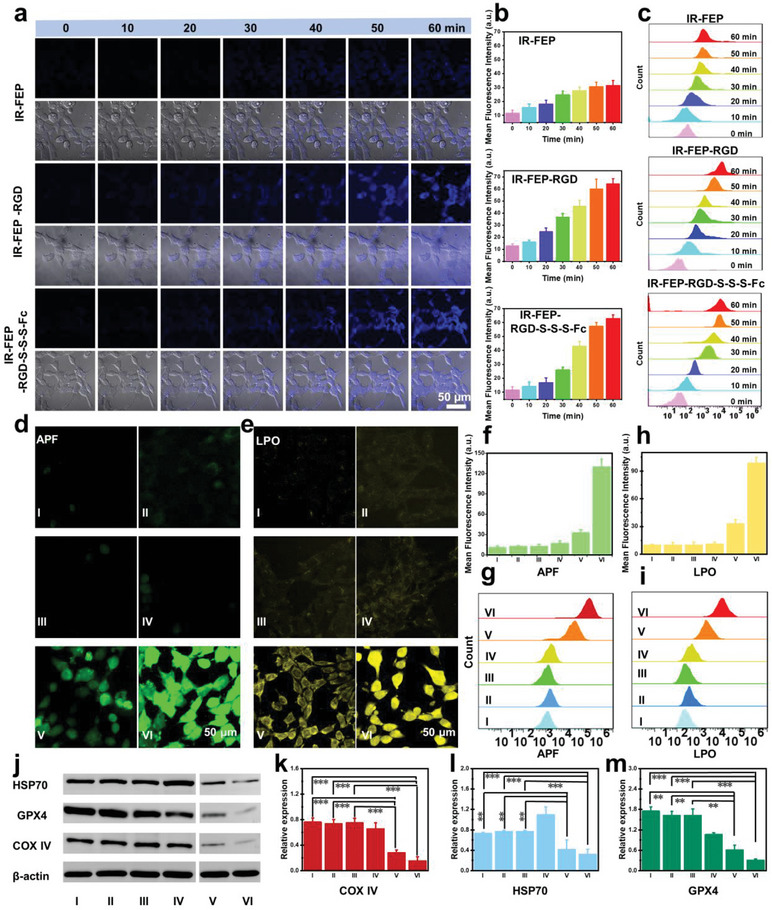
Assessment of the cellular •OH generation (APF), LPO, H_2_S level, and tumor targeting ability. a) CLSM images of 4T1 cells incubated with IR‐FEP, IR‐FEP‐RGD, and IR‐FEP‐RGD‐S‐S‐S‐Fc for 0–1 h. b) The MFI data of Figure 3a. c) Flow cytometric analysis of 4T1 cells incubated with IR‐FEP, IR‐FEP‐RGD, and IR‐FEP‐RGD‐S‐S‐S‐Fc for 0–1 h. d) 4T1 cells were incubated with different treatments. The green colors denote APF. e) 4T1 cells were incubated with different treatments. The yellow colors denote Liperfluo. f) The MFI data of Figure 3d. g) Flow cytometric analysis of the cellular •OH generation in 4T1 cells with different treatments. h) The MFI data of Figure 3e. i) Flow cytometric analysis of the LPO in 4T1 cells with different treatments. j) Western blot analysis on the expression levels of COX IV, HSP70, and GPX4 of 4T1 cells incubated with different treatments. Quantitative western blot analysis on the expression levels of k) COX IV, l) HSP70, and m) GPX4 of 4T1 cells in Figure 3j (original blots are available in Figure S38, Supporting Information). The treatments methods of different groups in the figure are as follows: I: PBS − L, II: PBS + L, III: IR‐FEP‐RGD − L, IV: IR‐FEP‐RGD + L, V: IR‐FEP‐RGD‐S‐S‐S‐Fc − L, VI: IR‐FEP‐RGD‐S‐S‐S‐Fc + L. + L, and − L are present as 808 nm laser/non‐laser irradiation. The significance of the difference of more than two groups was determined via ANOVA‐LSD *post hoc* test. ^*^ *P* < 0.05, ^**^ *P* < 0.01, and ^***^ *P* < 0.001. Error bars: mean ± SD (*n* = 4).

The tumor cell targeting ability of IR‐FEP‐RGD‐S‐S‐S‐Fc was demonstrated by incubating 4T1 (mouse breast cancer cells) with IR‐FEP, IR‐FEP‐RGD, and IR‐FEP‐RGD‐S‐S‐S‐Fc, respectively. The visualized cell targeting and uptake every 10 min were presented by confocal laser‐scanning microscope (CLSM) (*λ*_ex_/*λ*_em_ = 405/450 nm) (As shown in Figure S35, Supporting Information IR‐FEP‐RGD‐S‐S‐S‐Fc has a strong absorption at 350–405 nm and emits fluorescence at ≈470 nm, which can be directly used for CLSM imaging). Compared with the IR‐FEP group, the IR‐FEP‐RGD and IR‐FEP‐RGD‐S‐S‐S‐Fc group exhibited stronger fluorescence over time (**Figure** **3**a), which indicated a more efficient internalization of IR‐FEP‐RGD and IR‐FEP‐RGD‐S‐S‐S‐Fc for tumor cells owing to the active targeting ability of cRGDfk peptide. Moreover, the mean fluorescence intensity of the IR‐FEP‐RGD group and IR‐FEP‐RGD‐S‐S‐S‐Fc group have a sevenfold increase approximately (the IR‐FEP group is threefold) compared to their initial intensity (Figure 3b). According to the confocal laser‐scanning microscope (CLSM) of tumor targeting ability, the quantitative comparison indicated that the mean fluorescence intensity (MFI) of the cRGDfk‐modified group presents almost two times higher than the control group. Also, the isolated 4T1 cells were collected and analyzed by flow cytometry (*λ*_ex_ = 405 nm). The results were also consistent with the CLSM results (Figure 3c). The flow cytometry analysis also showed that the targeting efficiency of the cells was enhanced by ≈2.5 fold after coupling cRGDfk in 60 min (Figure S36, Supporting Information). In contrast, when HC11 mammary epithelium (HC11 cells, as a normal cell analogy) substituted 4T1 cells, the fluorescence intensity of IR‐FEP‐RGD‐S‐S‐S‐Fc after cell incubation was extremely low and almost unchanged (Figure S37a,b, Supporting Information). These results suggested that the modification of the cRGDfk peptide dramatically improved the accumulation of IR‐FEP‐RGD‐S‐S‐S‐Fc in the tumor cells. In addition, cRGD pre‐blocking experiment has also been carried out on 4T1 cells to prove this point. As shown in Figure S37c,d (Supporting Information), after blocking integrin αvβ3 by 10 × excess free cRGDfk peptide, the fluorescence intensity of IR‐FEP‐RGD‐S‐S‐S‐Fc in tumor cells was significantly reduced. Thus, this result proves that molecular targeting was enhanced after cRGDfk peptide modification.

The above experimental results demonstrated the efficient active targeting ability of IR‐FEP‐RGD‐S‐S‐S‐Fc to tumor cells due to cRGDfk peptide modifications. Notably, the IR‐FEP‐RGD‐S‐S‐S‐Fc presented a slower fluorescence enhancement than the IR‐FEP‐RGD, which may be attributed to the gradual removal of short‐wave fluorescence quenching by GSH under the PET mechanism. Furthermore, the NIR‐II fluorescence images indicated that 4T1 cells in the IR‐FEP‐RGD‐S‐S‐S‐Fc group obtained fluorescence lighting after 1 h, while not obtained in HC11 cells. In addition, N‐ethylmaleimide (NEM, a known expression inhibitor for GSH, 1.0 mm) or 10 × excess free RGD (block the specific binding of RGD and integrin αvβ3) could cause a dramatic decrease of fluorescence (Figure S37e, Supporting Information). These results demonstrated that IR‐FEP‐RGD‐S‐S‐S‐Fc achieved active molecular targeting ability to tumor cells through cRGDfk peptide, resulting in fluorescence activation in the tumor region owing to the elimination of PET quenching process by breakage of trisulfide bonds caused by the high concentration of GSH. However, low GSH concentrations in normal cells fail to achieve cleavage, causing persistent quenched fluorescence.

To evaluate the intracellular •OH level generated by IR‐FEP‐RGD‐S‐S‐S‐Fc, a fluorescent turn‐on probe, Aminophenyl fluorescein (APF),^[^ ^40^ ^]^ was employed. Confocal laser images of 4T1 cells incubated with IR‐FEP‐RGD‐S‐S‐S‐Fc indicated that the green fluorescence of IR‐FEP‐RGD‐S‐S‐S‐Fc + L group enhanced significantly compared with that of the IR‐FEP‐RGD‐S‐S‐S‐Fc − L group (Figure 3d), and the Mean fluorescence intensity (MFI) of the former was 3.97 times than that of the latter (Figure 3f), demonstrating elevated •OH levels induced by the photothermal effect. The intracellular •OH levels were further investigated by the flow cytometry analysis. The results revealed that the APF fluorescence intensity was significantly enhanced in the IR‐FEP‐RGD‐S‐S‐S‐Fc + L group (Figure 3g) compared with IR‐FEP‐RGD and PBS groups, which further validated that the laser irradiation enhances the production of hydroxyl radicals in the cells.

Cellular Lipid Peroxidation Assay Kit (Liperfluo)^[^ ^39^ ^]^ was used to measure the intracellular lipid peroxide (LPO). According to Figure 3e, the IR‐FEP‐RGD‐S‐S‐S‐Fc + L group displayed enhanced fluorescence (3.06 times by MFI) compared with IR‐FEP‐RGD‐S‐S‐S‐Fc − L group (marked as yellow) (Figure 3h), presenting the increased LPO owing to the elevation of intracellular •OH levels under laser irradiation. Flow cytometry analysis also revealed a similar trend (Figure 3i). These results demonstrated that the generation of LPO accompanied by the •OH levels induced by IR‐FEP‐RGD‐S‐S‐S‐Fc in tumor cells also appeared as an effect of laser activation.

The intracellular release of H_2_S was evaluated by an H_2_S‐specific fluorescence probe WSP‐1 (marked as orange). As presented in Figure S38 (Supporting Information), apparent fluorescence in the cells can be observed after incubation with IR‐FEP‐RGD‐S‐S‐S‐Fc (50 µm), while no H_2_S signal revealed in other control groups. Under the 808 nm laser irradiation, enhanced fluorescence indicated a higher concentration of H_2_S intracellularly. The results presented that IR‐FEP‐RGD‐S‐S‐S‐Fc internalized by cancer cells could generate an amount of H_2_S under laser irradiation owing to the cleavage of trisulfide bonds by GSH.

The mitochondria are the central location site of cellular respiration and energy metabolism. In mitochondria, adenosine triphosphate (ATP) is the primary energy source. It has been reported that H_2_S would engender acute toxicity by suppressing mitochondrial cytochrome c oxidase (COX IV), thus decreasing the production capacity of ATP.^[^ ^21^ ^]^ Meanwhile, H_2_S downregulates the expression of HSP70 and reverses the tumor heat resistance. Therefore, the effect of IR‐FEP‐RGD‐S‐S‐S‐Fc on the activity of COX IV and HSP70 was investigated. As shown in Figure 3j–l immunofluorescent staining and western blot analysis revealed the cellular COX IV and HSP70 levels with different treatments (original blots are available in Figure S38, Supporting Information). The results indicated that the expressions of COX IV and HSP70 in the IR‐FEP‐RGD‐S‐S‐S‐Fc groups were lower than those of other groups. Moreover, the IR‐FEP‐RGD‐S‐S‐S‐Fc + L group showed the lowest fluorescence intensity and gray value, demonstrating that the H_2_S gas could suppress COX IV and HSP70 expressions and cause the improvement of the HPTT efficiency. The ATP level of mitochondria was measured by ATP Assay Kit with absorption at 340 nm after incubation with 4T1 tumor cells. In contrast with the PBS group, the IR‐FEP‐RGD‐S‐S‐S‐Fc + L group reveals significantly inhibited ATP production (Figure S39c, Supporting Information), demonstrating that respiration was impaired.

The extracellular GSSH levels were measured via a DTNB (5,5′‐dithiobis‐(2‐nitrobenzoic acid)) assay kit. After adding the IR‐FEP‐RGD‐S‐S‐S‐Fc, there was a distinct depletion of GSH compared to the PBS group (Figure S39a, Supporting Information). Meanwhile, 4T1 cells were continuously cultivated for 8 h with different treatments to verify the intracellular GSH level changes. The highest consumption emerged in the IR‐FEP‐RGD‐S‐S‐S‐Fc + L group. The depletion level of intracellular GSH in the IR‐FEP‐RGD‐S‐S‐S‐Fc − L group was less significant than the IR‐FEP‐RGD‐S‐S‐S‐Fc + L group, which means the trimodal synergistic therapy resulted in a higher degree of GSH depletion (Figure S39b, Supporting Information). Correspondingly, As shown in Figure 3j,m, immunofluorescent staining and Western blot analysis revealed the cellular GPX4 levels with different treatments (original blots are available in Figure S38, Supporting Information). The control group's GPX4 concentration maintained a higher level than other groups, demonstrating that GPX4 retained normal activity in PBS. Either fluorescence intensity or relative expression in western blot analysis for the IR‐FEP‐RGD + L group, the GPX4 level was lower, demonstrating that the photothermal effect reduced the expression of GPX4. Moreover, the IR‐FEP‐RGD‐S‐S‐S‐Fc + L group showed the lowest expression of GPX4 by fluorescence intensity and quantitative western blot analysis, meaning that IR‐FEP‐RGD‐S‐S‐S‐Fc combined with laser irradiation could cause ferroptosis through the GPX4 pathway.

### 2.5 Cytotoxicity and Cell Apoptosis of IR‐FEP‐RGD‐S‐S‐S‐Fc

**Figure 4:**
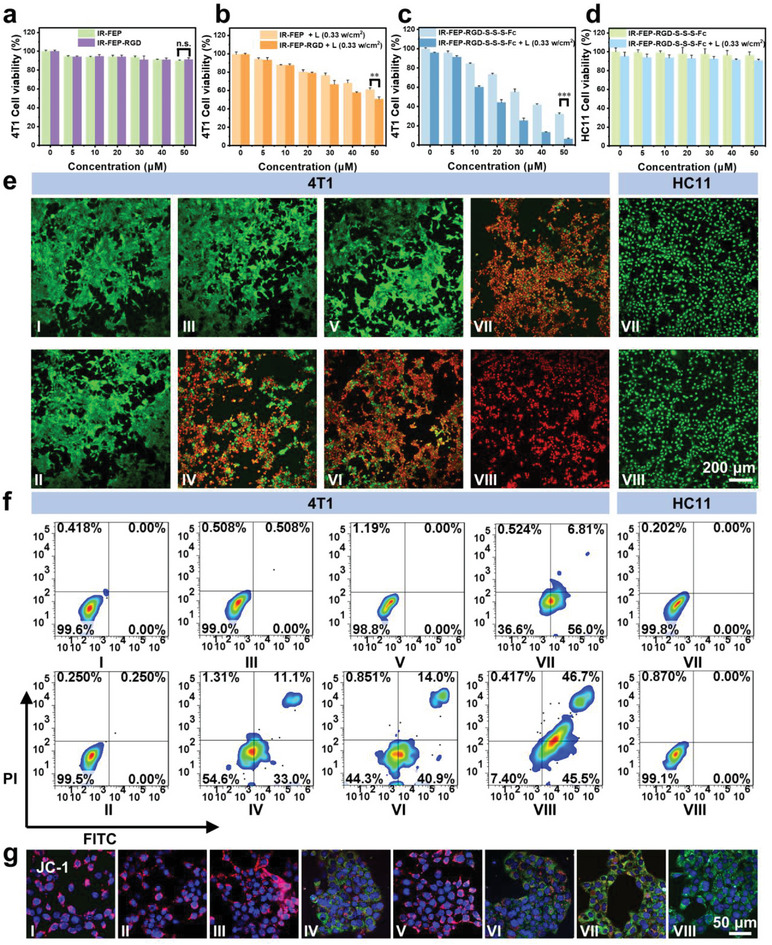
Assessment of cytotoxicity and apoptosis. Cell viability of 4T1 cells treated with a) IR‐FEP and IR‐FEP‐RGD, b) IR‐FEP +L and IR‐FEP‐RGD +L, and c) IR‐FEP‐RGD‐S‐S‐S‐Fc at various concentrations. d) Cell viability of HC11 cells treated with IR‐FEP‐RGD‐S‐S‐S‐Fc at various concentrations and different treatments (+ L/− L, 808 nm, 0.33 W cm^−2^). e) Calcein‐AM (green)/PI (red) staining with different treatments f) Flow cytometric analysis of apoptosis levels with different treatments. g) CLSM observation on the changes in mitochondrial membrane potential of 4T1 cells (JC‐1 stained) after incubation with different treatments. The treatment methods of different groups in the figure are as follows: I: PBS − L, II: PBS + L, III: IR‐FEP − L, IV: IR‐FEP + L, V: IR‐FEP‐RGD − L, VI: IR‐FEP‐RGD + L, VII: IR‐FEP‐RGD‐S‐S‐S‐Fc − L, VIII: IR‐FEP‐RGD‐S‐S‐S‐Fc + L. + L, and − L are present as 808 nm laser/non‐laser irradiation. The concentration of samples in each group is 50 µm. The significance of the difference between two groups was determined via Student's *t*‐test. ^*^ *P* < 0.05, ^**^ *P* < 0.01, and ^***^ *P* < 0.001. Error bars: mean ± SD (*n* = 4).

The cytotoxicity of IR‐FEP‐RGD‐S‐S‐S‐Fc was evaluated with 4T1 and HC11 cells by the Cell Counting Kit‐8 (CCK8) assay. As shown in **Figure** **4**a–c, the viabilities of 4T1 cells exhibited a stepwise decrease with the increased IR‐FEP‐RGD‐S‐S‐S‐Fc. A low survival rate (8%) at 50 µm with a 0.33 w cm^−2^ laser irradiation was acquired. In contrast, the viability in the IR‐FEP and IR‐FEP‐RGD groups without laser irradiation was over 90.04%, and IR‐FEP/IR‐FEP‐RGD with laser irradiation group, the survival rate was also over 50.6% for tumor cells. On the contrary, the HC11 cell viability was over 90% at 50 µm of the IR‐FEP‐RGD‐S‐S‐S‐Fc with laser irradiation (Figure 4d), presenting its low toxicity to normal cells. As illustrated in Figure 4e,f, similar results could also be seen in live/dead cell staining and flow cytometry analyses, confirming that IR‐FEP‐RGD‐S‐S‐S‐Fc has precise damage ability to tumor cells and an outstanding therapeutic effect of combined CDT/HPTT/GT.

The mitochondrial membrane potential could be imaged by the JC‐1 staining assay (Mitochondrial Membrane Potential Assay Kit) (Figure 4g). The results indicated that the mitochondrial membrane potential in the IR‐FEP‐RGD‐S‐S‐S‐Fc + L group has significantly decreased, indicating mitochondrial dysfunction caused by CDT/HPTT/GT.^[^ ^21^, ^25^, ^40^, ^41^ ^]^ Taken together, IR‐FEP‐RGD‐S‐S‐S‐Fc could selectively active target tumor cells instead of normal cells by cRGDfk peptide and mediate light‐induced H_2_S‐mediated GT, and HPTT enhanced CDT to exhibit excellent antitumor cell performance in vitro with mitochondrial dysfunction.

### 2.6 In Vivo Imaging and Pharmacokinetics of IR‐FEP‐RGD‐S‐S‐S‐Fc

**Figure 5:**
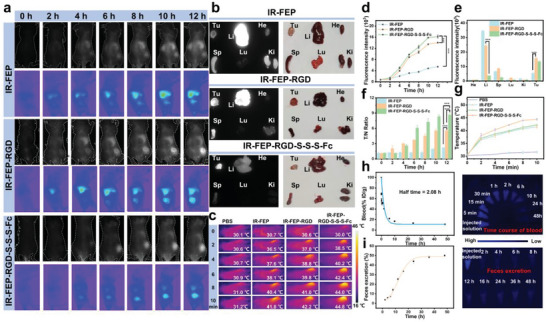
In vivo imaging and pharmacokinetics. a) NIR‐II fluorescence images (prone + lateral) of the 4T1 tumor‐bearing mice (different time) post‐injection of IR‐FEP, IR‐FEP‐RGD, IR‐FEP‐RGD‐S‐S‐S‐Fc. b) Fluorescent images of tumors and primary organs in mice (collected at 12 h) (*λ*_ex_: 808 nm laser, filters: 900Lp, exposure time: 120 ms). c) Photothermal imaging of 4T1 tumor‐bearing mice post‐injection with PBS, IR‐FEP, IR‐FEP‐RGD, IR‐FEP‐RGD‐S‐S‐S‐Fc for 12 h and then treated with *λ*_ex_: 808 nm laser (0.33 W cm^−2^, 10 min). The fluorescence intensity of d) tumors and e) major organs. f) The tumor to normal tissue (T/N) ratio in tumor‐bearing mice was probed at different times after injection. g) The corresponding temperature of (c). h) Time course of blood concentration of IR‐FEP‐RGD‐S‐S‐S‐Fc ‐treated mic in 48 h and corresponding fluorescence images of blood samples (In GSH 10 mm environment). i) Cumulative feces excretion and corresponding fluorescence imaging of IR‐FEP‐RGD‐S‐S‐S‐Fc ‐treated mice at 2–48 h after intravenous injection (In GSH 10 mm environment). The dose of each group of samples in the figure is 100 µL (50 µm). Three Balb/c mice for each group were used in all experiments. The significance of the difference between two groups was determined via Student's *t*‐test. The significance of the difference of more than two groups was determined via ANOVA‐LSD *post hoc* test. ^*^ *P* < 0.05, ^**^ *P* < 0.01, and ^***^ *P* < 0.001. Error bars: mean ± SD (*n* = 3).

Based on the excellent selectivity of tumor cells in vitro, the in vivo NIR‐II fluorescence imaging performance of IR‐FEP‐RGD‐S‐S‐S‐Fc was investigated based on the 4T1 tumor‐bearing mice model. As presented in **Figure** **5**a,d, the tumor signal increased considerably over time after intravenous injection of IR‐FEP, IR‐FEP‐RGD‐S‐S‐S‐Fc, and IR‐FEP‐RGD. A plateau was eventually reached at 12 h. After being euthanized and dissected, the biodistribution of injected IR‐FEP‐RGD‐S‐S‐S‐Fc in the mice was evaluated by the fluorescence images of the ex vivo organ. As shown in Figure 5b,e, IR‐FEP the IR‐FEP‐RGD‐S‐S‐S‐Fc and IR‐FEP‐RGD groups showed extremely high tumor aggregation compared to the IR‐FEP group, confirming the excellent targeting effect of the RGD‐modified fluorescent molecules in vivo. The IR‐FEP‐RGD‐S‐S‐S‐Fc showed slightly lower tumor accumulation than that of IR‐FEP‐RGD, which could be ascribed to tough recovery from PET‐based fluorescence quenching after GSH cleavage completely. Mononuclear phagocyte system (MPS) refers to diverse cell populations, such as bone marrow progenitors, blood monocytes, and tissue macrophages, that are activated by non‐endogenous species and participate in the clearance of these foreign nanoparticles.^[^ ^28^ ^]^ However, as the spleen and liver are major organs of the MPS system, the accumulation of IR‐FEP and IR‐FEP‐RGD was increased in the liver and spleen.^[^ ^32^, ^42^ ^]^ Interestingly and surprisingly, the signal backgrounds of the liver and spleen were indeed lower in the IR‐FEP‐RGD‐S‐S‐S‐Fc group compared to the IR‐FEP and IR‐FEP‐RGD groups (by the lateral view and organ fluorescence analysis). These results indicated that the fluorescence molecule IR‐FEP‐RGD‐S‐S‐S‐Fc was concentrated in the liver, and the fluorescence was quenched due to the presence of the PET mechanism, which effectively reduces the background. However, with the extension of time, some of the tumor tissue‐activated probes were also enriched in the liver with the blood flow, resulting in a faint signal in the liver and spleen. Additionally, quantitative ROI analysis further assessed the ratio of tumor to normal tissue (T/N) signal (Figure 5f). The results showed that IR‐FEP‐RGD‐S‐S‐S‐Fc demonstrated a T/N ratio of up to 8.60 at 12 h with a lower dose (50 µm, 100 µL), superior to our previous work (200 µL of 120 µm IR‐FEP to achieve a T/N of 5.45 after 24 h).^[^ ^32^ ^]^ The above results indicated that the IR‐FEP‐RGD‐S‐S‐S‐Fc showed great NIR‐II fluorescence imaging performances with high precision and contrast, thus realizing specific localization of tumor region.

The in vivo photothermal effect of the NIR‐II fluorescence imaging‐guided molecular phototheranostic platform was investigated after 12 h post‐injection of IR‐FEP‐RGD‐S‐S‐S‐Fc (808 nm laser irradiation, 10 min, 0.33 W cm^−2^). The temperature of the tumor region of IR‐FEP‐RGD‐S‐S‐S‐Fc treated mice increased to 44.8 °C. The tumor temperature in mice with PBS, IR‐FEP, and IR‐FEP‐RGD treated raised to ≈31.2, 41.8, and 42.2 °C (Figure 5c,g). These results suggested that IR‐FEP‐RGD‐S‐S‐S‐Fc has good hypothermal photothermal therapy (HPTT) in vivo.

To investigate the in vivo pharmacokinetics of IR‐FEP‐RGD‐S‐S‐S‐Fc, BALB/c mice (*n* = 3) were intravenously injected with IR‐FEP‐RGD‐S‐S‐S‐Fc, and the extraction of blood and feces samples were monitored at different times after treated with GSH. As shown in Figure 5h,i, the blood circulation and feces excretion of IR‐FEP‐RGD‐S‐S‐S‐Fc were noted during the 48 h that followed intravenous injection. The half‐time of circulation for IR‐FEP‐RGD‐S‐S‐Fc was ≈2.08 h. In addition, the feces excretion data showed that ≈49.8% IR‐FEP‐RGD‐S‐S‐S‐Fc was excreted via feces within 48 h post intravenous injection, demonstrating slow processing of hepatic excretion.^[^ ^32^, ^43^ ^]^

### 2.7 In Vivo Assessment of Antitumor Efficiency

**Figure 6:**
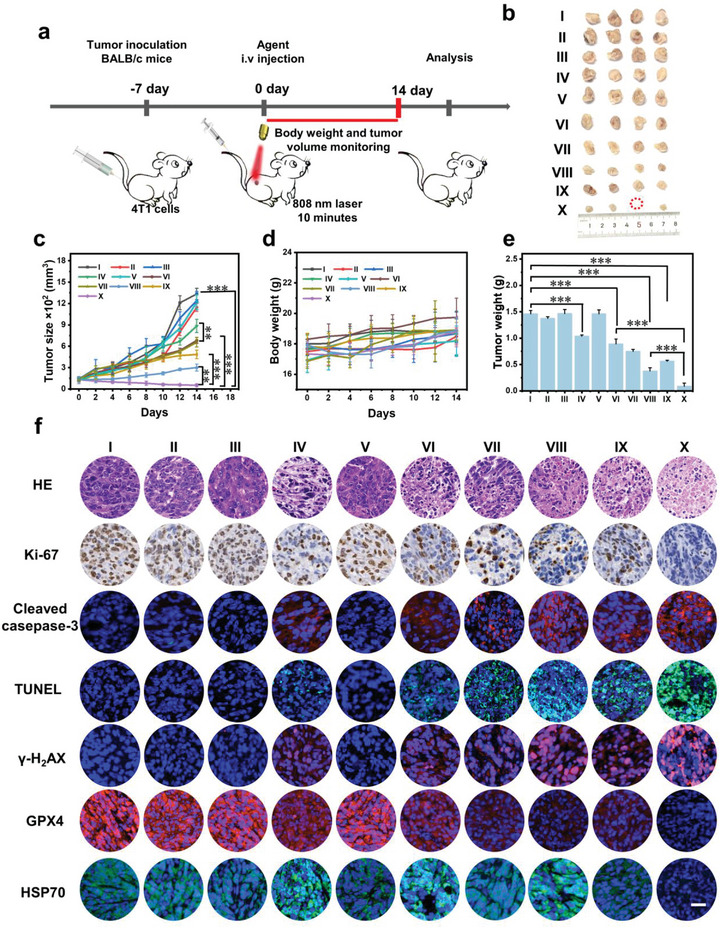
In vivo assessment of antitumor efficiency. a) Schematic of IR‐FEP‐RGD‐S‐S‐S‐Fc based CDT/HPTT/GT combined therapy to inhibit 4T1 subcutaneous tumor model. b) Pictures of tumors in different treatment groups of mice after 14 days. c) Relative tumor volumes and d) Relative body weight of different treatment groups. e) The average tumor weight and f) Immunofluorescence images of tumor tissue after 14 days of treatments, Scale bar = 40 µm. The treatments methods of different groups in the figure are as follows: I: PBS − L, II: PBS + L, III: IR‐FEP − L, IV: IR‐FEP + L, V: IR‐FEP‐RGD − L, VI: IR‐FEP‐RGD + L, VII: IR‐FEP‐RGD‐Fc − L, VIII: IR‐FEP‐RGD‐Fc + L.IX: IR‐FEP‐RGD‐S‐S‐S‐Fc − L, X: IR‐FEP‐RGD‐S‐S‐S‐Fc + L. + L, and − L are present as 808 nm laser/non‐laser irradiation. The significance of the difference between two groups was determined via Student's *t*‐test. The significance of the difference of more than two groups was determined via ANOVA‐LSD *post hoc* test. ^*^ *P* < 0.05, ^**^ *P* < 0.01, and ^***^ *P* < 0.001. Error bars: mean ± SD (*n* = 4). The dose of each group of samples in the figure is 100 µL (50 µm).

The above results encouraged us to explore in vivo therapy on 4T1 tumor‐bearing mice. The tumor volumes and body weights of the mice were recorded every 2 days up to day 14 post‐injection (**Figure** **6**a). The results indicated that tumor sizes of the IR‐FEP − L and IR‐FEP‐RGD − L groups were almost in the same range as the PBS group, suggesting the IR‐FEP‐RGD − L group nearly has no treatment effect (Figure 6b,c). The IR‐FEP‐RGD‐S‐S‐S‐Fc − L group presented a low tumor inhibition effect than the IR‐FEP‐RGD‐S‐S‐S‐Fc + L group, demonstrating the superiority of the photothermal‐enhanced therapeutic process. The IR‐FEP‐RGD‐S‐S‐S‐Fc + L group also displayed a better tumor inhibition effect than the IR‐FEP‐RGD + L group, confirming that the combined CDT and GT enhanced the therapeutic effect. The tumor inhibition rate of the IR‐FEP+L (HPTT) group was 28.33%. In comparison, the tumor inhibition rate of the IR‐FEP‐RGD+L group was slightly increased to 40.36%, which proved that the introduction of targeting cRGD has a weak promotion in therapeutic effect. However, the inefficiency of the single HPTT can not obtain a satisfactory treatment. Therefore, multiple treatment strategies were employed to enhance the therapeutic effect. The tumor inhibition rate of the IR‐FEP‐RGD‐Fc group (CDT) increased to 50.56%. The tumor inhibition rate of the IR‐FEP‐RGD‐Fc+L group (CDT+HPTT) increased to 75.33%, which indicated an effective tumor inhibition with the synergistic effect of targeted CDT/HPTT. The tumor inhibition rate of the IR‐FEP‐RGD‐S‐S‐S‐Fc group (CDT+ GT) slightly increased to 59.85%, which indicated that in the absence of thermal intensification, the therapeutic effect of CDT/GT was limited and did not achieve a very good therapeutic effect. Finally, the tumor inhibition rate of the IR‐FEP‐RGD‐S‐S‐S‐Fc+L group (CDT +HPTT+GT) achieved a tumor inhibition rate of 96.58%, which proved that the effects of CDT and GT were extremely enhanced under the NIR irradiation reinforcement (Figure S40, Supporting Information). The monitoring of tumor weight also presented a similar tendency (Figure 6e). The body weights of all mice remained stable throughout the treatments, indicating that the prepared molecular phototheranostic agents were non‐toxicity (Figure 6d).

To study the molecular mechanism of the molecular phototheranostic platform, tumor tissues were collected and subjected to histological examination. The results demonstrated that the IR‐FEP‐RGD‐S‐S‐S‐Fc + L group showed an enhanced therapeutic effect than the other groups by the number of tumor cell damage with nuclear membrane fragmentation and nuclei shrinkage in H&E staining tissues. In addition, the tumor sections showed more apoptosis through TdT‐mediated dUTP‐biotin nick end labeling (TUNEL) and cleaved caspase‐3 (caspase‐3). The proliferation marker Ki‐67 was significantly downregulated, indicating a significant inhibiting of tumor cell proliferation and more severe apoptosis (Figure 6f).

Generally, the heat endurance of tumors is primarily owed to HSPs, the expression of which was determined by the energy supply of ATP.^[^ ^27^ ^]^ Compared with the PBS + L group, IR‐FEP + L and IR‐FEP‐RGD + L groups showed slightly higher expression of HSP70 due to the temperature increase. In contrast, lower HSP70 expression was detected in PBS − L group, IR‐FEP − L, IR‐FEP‐RGD − L, and IR‐FEP‐RGD‐S‐S‐S‐Fc − L groups. It is worth noting that IR‐FEP‐RGD‐S‐S‐S‐Fc + L groups demonstrated deficient HSP70 expression, which could be attributed to the low expression of COX IV and mitochondrial dysfunction induced by the H_2_S produced by IR‐FEP‐RGD‐S‐S‐S‐Fc and GSH (Figure 6f). The lower expression of COX IV and mitochondrial dysfunction would decrease ATP production, thus downregulating the expression of HSP70 and reversing the tumor heat resistance, synergistically enhancing the HPTT effect.^[^ ^24a^ ^]^ Meanwhile, phosphorylated H2A histone family member X (γ‐H2AX) was upregulated after laser radiation, demonstrating that the HPTT/CDT/GT combined therapy could activate ferroptosis to kill tumor cells through DNA damage. As anticipated, the expression of GPX4 was down‐regulated after laser radiation, demonstrating the presented therapeutic mechanism of CDT/HPTT/GT ‐activated ferroptosis.^[^ ^20^, ^28^, ^37^ ^]^

Collectively, in vivo and in vitro tumor suppression experiments confirmed that the molecular phototherapy platform IR‐FEP‐RGD‐S‐S‐S‐Fc can initiate tumor CDT/HPTT/GT combined therapy through high concentrations of intratumoral GSH and effectively enhance it in vivo by local NIR irradiation, thus achieving excellent tumor suppression effects. Notably, IR‐FEP‐RGD‐S‐S‐S‐Fc exhibits good biocompatibility based on the H&E‐stained images and biochemical analysis of blood (Figure S41, Supporting Information). Hemolysis experiments showed no evident hemolysis was detected even at the high concentration of 400 µm. (Figure S42, Supporting Information). These results demonstrated that IR‐FEP‐RGD‐S‐S‐S‐Fc could be a precise and efficient CDT/HPTT/GT combination therapy platform.

## 3 Conclusion

In conclusion, a TME activatable NIR‐II fluorescence imaging‐guided phototheranostic nanoplatform (IR‐FEP‐RGD‐S‐S‐S‐Fc) was rationally designed and developed by multiple molecular synthesis designs. We have explored the relationship between PET efficiency and distances of the fluorophore and quenched agent and found that the shorter the length, the stronger the quenching. The as‐synthesized IR‐FEP‐RGD‐S‐S‐S‐Fc achieved active tumor targeting via the cRGDfk peptide. Through GSH shearing to the trisulfide bond, the IR‐FEP‐RGD‐S‐S‐S‐Fc achieves tumor site cascade‐specific illumination. Importantly, IR‐FEP‐RGD‐S‐S‐S‐Fc obtains CDT/HPTT/GT trimodal synergistic enhancement with laser irradiation, which can further enhance the GSH depletion and •OH generation. The released H_2_S amplifies oxidative stress and inhibits COX IV activity. Low expression of COX IV would diminish intracellular ATP supply and decrease the expression of ATP‐dependent HSPs, which remarkably reverse tumor heat resistance and enhance hypothermal photothermal performance. Both in vitro and in vivo experiments confirmed the remarkable therapeutic effects of IR‐FEP‐RGD‐S‐S‐S‐Fc on 4T1 cells and 4T1 tumor‐bearing mice. Hence, the IR‐FEP‐RGD‐S‐S‐S‐Fc with tumor‐specific light‐up NIR‐II fluorescence imaging and CDT/HPTT/GT co‐enhanced therapy are promising candidates for activatable tumor diagnosis and treatment.

## 4 Experimental Section

The experimental section is available in the Supporting Information. All animal experiments were approved by the University of South China Animal Experiment Ethics Review and the Health Guide for the Care and Use of Laboratory Animals of National Institutes. The assigned accreditation number of the laboratory is 2023027.

### Statistical Analysis

The results were expressed as mean ± standard deviation (SD). Analysis for two groups were calculated using an unpaired two‐tailed Student's *t*‐test; comparisons of more than two groups were determined via ANOVA‐LSD *post hoc* test. The significance was indicated by ^*^ *P* < 0.05, ^**^ *P* < 0.01, and ^***^ *P* < 0.001.

## Conflict of Interest

The authors declare no conflict of interest.

## Supporting information

Supporting Information

## Data Availability

The data that support the findings of this study are available in the supplementary material of this article.
