# Supplementary material for: Trisulfide Bond‐Mediated Molecular Phototheranostic Platform for “Activatable” NIR‐II Imaging‐Guided Enhanced Gas/Chemo‐Hypothermal Photothermal Therapy
Source: Adv Sci (Weinh). 2023 Nov 20;10(36):2304104. doi: 10.1002/advs.202304104 (PMC10754146; doi:10.1002/advs.202304104)
Supplement: Supplementary file 1 — Supporting Information [file ADVS-10-2304104-s001.pdf]

ADVANCED SCIENCE
Open Access

Supporting Information

for *Adv. Sci.*, DOI 10.1002/advs.202304104

Trisulfide Bond-Mediated Molecular Phototheranostic Platform for "Activatable" NIR-II Imaging-Guided Enhanced Gas/Chemo-Hypothermal Photothermal Therapy

Gui-long Wu, Fen Liu, Na Li, Qian Fu, Cheng-kun Wang, Sha Yang, Hao Xiao, Li Tang, Feirong Wang, Wei Zhou, Wenjie Wang, Qiang Kang, Zelong Li, Nanyun Lin, Yinyin Wu, Guodong Chen, Xiaofeng Tan* and Qinglai Yang**

Supporting Information

Trisulfide bond-mediated Molecular Phototheranostic Platform for “activatable” NIR-II Imaging-Guided Enhanced Gas/Chemo- Hypothermal Photothermal Therapy

Gui-long Wu[1], Fen Liu[1], Na Li[1], Qian Fu[1], Cheng-kun Wang[1], Sha Yang[1], Hao Xiao[1], Li Tan [1,4], Feirong Wang[1], Wei Zhou[1], Wenjie Wang[1], Qiang Kang[1], Zelong Li[1], Nanyun Lin[1], Yinyin Wu[1], Guodong Chen[2], Xiaofeng Tan [1,3,5]*, Qinglai Yang[1,2,3,5]**

[1]Center for Molecular Imaging Probe, Hunan Province Key Laboratory of Tumor Cellular and Molecular Pathology, Cancer Research Institute, Hengyang Medical School, University of South China, Hengyang, Hunan 421001, China.

[2]Department of Hepatopancreatobiliary Surgery, The First Affiliated Hospital, Hengyang Medical School, University of South China, Hengyang, Hunan, 421001, China.

[3]National Health Commission Key Laboratory of Birth Defect Research and Prevention, Hunan Provincial Maternal and Child Health Care Hospital, Changsha 410008, Hunan, China.

[4]Key Laboratory of Tropical Medicinal Plant Chemistry of Ministry of Education, College of Chemistry and Chemical Engineering, Hainan Normal University, Haikou, Hainan 571158, China.

[5]MOE Key Lab of Rare Pediatric Diseases, Hengyang Medical School, University of South China, Hengyang, Hunan, 421001, China.

Correspondence should be addressed to:

chenguodong@usc.edu.cn, tanxiaofeng@usc.edu.cn, qingyu513@usc.edu.cn

Table of Contents

1. Experimental section 4
1.1 Materials and reagents 4
1.2 General measurements.. 5
1.3 Synthesis and characterization of the molecular fluorophores 6
1.4 Photothermal conversion performance of IR-FEP-RGD-S-S-S-Fc 20
1.5 Measurement of quantum yield.. 21
1.6 Chemodynamic activity of IR-FEP-RGD-S-S-S-Fc. 22
1.7 The generation of intracellular •OH and lipid peroxide (LPO)........................... 23
1.8 Controlled release of H_2S and intracellular H_2S detection 23
1.9 Tumor cell targeting assays.. 24
1.10 Evaluation of cytochrome c oxidase (COX IV) 24
1.11 Intracellular and extracellular GSH quantification... 25
1.12 Investigation on the mitochondrial membrane potential 25
1.13 Cell viability assay... 26
1.14 Animal tumor models 26
1.15 *In vivo* NIR-II fluorescence and thermal imaging.. 27
1.16 Pharmacokinetics, biodistribution, and excretion of IR-FEP-RGD-S-S-S-Fc.... 27
1.17 Antitumor therapy *in vivo* 28
1.18 Biochemistry analysis of IR-FEP-RGD-S-S-S-Fc 28
Figure S1. ^{1}H NMR of compound 2... 29
Figure S2. ^{13}C NMR of compound 2.. 30
Figure S3. HMRS of compound 2.. 30
Figure S4. ^{1}H NMR of compound 5.. 31
Figure S5. ^{13}C NMR of compound 5.. 31
Figure S6. HMRS of compound 5.. 32
Figure S8. ^{13}C NMR of compound IR-FE (C_{12}) 33
Figure S9. HRMS of compound IR-FE (C_{12}). 33
Figure S10. ^{1}H NMR of compound IR-FE-Fc (C_{12}) 34
Figure S11. ^{13}C NMR of compound IR-FE (C_{12}).. 35
Figure S12. HRMS of compound IR-FE-Fc (C_{12}) 35
Figure S13. ^{1}H NMR of compound IR-FEP-Fc.. 36
Figure S14. ^{13}C NMR of compound IR-FEP-Fc... 36
Figure S15. ^{1}H NMR of compound IR-FEP 37
Figure S16. ^{13}C NMR of compound IR-FEP 38
Figure S17. ^{1}H NMR of compound IR-FEP-RGD -Fc... 38
Figure S18. ^{1}C NMR of compound IR-FEP-RGD-Fc... 39
Figure S19. ^{1}H NMR of compound 7.. 39
Figure S20. ^{13}C NMR of compound 7... 40
Figure S21. HRMS of compound 7... 40
Figure S22. ^{1}H NMR of compound Fc-S-S-S-COOH... 41
Figure S23. ^{13}C NMR of compound Fc-S-S-S-COOH.. 41
Figure S24. HRMS of compound Fc-S-S-S-COOH... 42

Figure S25. ^{1}H NMR of compound IR-FEP-RGD.. 42
Figure S26. ^{13}C NMR of compound IR-FEP-RGD... 43
Figure S27. ^{1}H NMR of compound IR-FEP-RGD-S-S-S-Fc 44
Figure S28. ^{13}C NMR of compound IR-FEP-RGD-S-S-S-Fc 45
Figure S29. ^{1}H NMR spectra of IR-FEP, IR-FEP-Fc, IR-FEP-RGD and IR-FEP-RGD-S-S-S-Fc in $CDCl_3$ 45
Figure S30. SEC elution traces of IR-FEP, IR-FEP-Fc, IR-FEP-RGD, and IR-FEP-RGD-S-S-S-Fc. 46
Figure S31. Relative fluorescence intensity ratios of IR-FEP-RGD-S-S-S-Fc (50 μM) with the addition of 10 mM of various amino acids. 47
Figure S32. GSH-triggered breakage of trisulfide bonds (a) and (b)HRMS spectra of IR-FEP-RGD-S-S-S-Fc after incubation with 10 mM GSH-containing release media. 48
Figure S33. (a) The temperature changes of different concentrations of IR-FEP-RGD-S-S-S-Fc (50 μM) under 808 nm laser irradiation (0.33 W/cm^2). (b) Heating curve of IR-FEP-RGD-S-S-S-Fc (50 μM) under 808 nm laser irradiation of different power densities. c) Infrared thermal images of IR-FEP-RGD-S-S-S-Fc of different concentrations. 49
Figure S34. (a) The absorption spectra of TMB aqueous solution with different treatments (I TMB + IR-FEP-RGD-S-S-S-Fc − L, II TMB + H_2O_2 + IR-FEP-RGD-S-S-S-Fc − L, III: TMB + H_2O_2 + IR-FEP-RGD-S-S-S-Fc + GSH, IV: TMB + H_2O_2 + IR-FEP-RGD-S-S-S-Fc +GSH + L, + L and − L are present as 808 nm laser/non-laser irradiation). (o) A652 TMB oxidation intensity of Figure S31a, Inset: images of relevant color variations). Error bars: mean ± SD (n = 4)......................... 50
Figure S35. (a) Normalized NIR absorption of IR-FEP-RGD-S-S-S-Fc in aqueous solution. (b) The fluorescence emission spectra of IR-FEP-RGD-S-S-S-Fc excited at 405 nm. 51
Figure S36. The MFI data of Figure 3c. 52
Figure S37. (a) CLSM images of HC11 cells incubated with IR-FEP-RGD-S-S-S-Fc 50 μM for 0-1 h. (b) Mean fluorescence intensity (MFI) of cells, which was quantified from (Figure S5a). Error bars: mean ± SD (n = 3). (c) CLSM images of 4T1 cells incubated with IR-FEP-RGD-S-S-S-Fc 50 μM with 500 μM RGD used for blocking integrin αvβ3 0-1 h. (d) Mean fluorescence intensity (MFI) of cells, which was quantified from (Figure S5c). Error bars: mean ± SD (n = 3). (e)The NIR-II fluorescence images and Semi-quantification of the fluorescence intensity of IR-FEP-RGD-S-S-S-Fc (50 μM) incubated with I: 4T1 cells, II: HC11 cells. III: 4T1 cells (+ 1 mM NEM)， and IV: 4T1 cells with 500 μM RGD used for blocking integrin αvβ3 after 1 h. 53
Figure S38. CLSM images of Intracellular H_2S detection using WSP-1 (marked as orange) as a probe and Immunofluorescent stained images of COX IV, HSP70 and GPX4 of 4T1 cells after incubation with different treatments................................. 54
Figure S39. (a) TNB intensity at A_{412} within 60 min in the environment of GSH 10 mM. Error bars: mean ± SD (n = 4). (b) The GSH level of 4T1 cells after incubation with I: PBS − L, II: PBS + L, III: IR-FEP-RGD − L, IV: IR-FEP-RGD + L, V:

IR-FEP-RGD-S-S-S-Fc − L, VI: IR-FEP-RGD-S-S-S-Fc + L. Error bars: mean ± SD (n = 4). (c) The ATP levels of the cell after treatment I: PBS − L, II: PBS + L, III: IR-FEP-RGD-S-S-S-Fc − L, IV: IR-FEP-RGD-S-S-S-Fc + L. Error bars: mean ± SD (n = 4). * $P < 0.05$, ** $P < 0.01$, and ***$P < 0.001$. 55
Figure S40. Contribution of different therapeutic modalities concluded from Figure 6b and 6c. Error bars: mean ± SD (n = 4). 56
Figure S41.. Hematological data and H&Estained images 56
Figure S42. Hemolysis test of IR-FEP-RGD-S-S-S-Fc. Error bars: mean ± SD (n = 4). 58
Figure S43. Western blot analysis on the expression levels of αvβ3 in 4T1 and HC11 cells Error bars: mean ± SD (n = 3). 59
Table S1. Molecule weight determined by SEC-MALLS. 60
Table S2. Comparison of detection parameters of IR-FEP-RGD-S-S-S-Fc with other GSH fluorescence response platforms.. 61
Reference 62

1. Experimental section

1.1 Materials and reagents

Aminophenyl fluorescein (APF), Calcein-AM/PI double stain kit, Annexin V-FITC assay kit, Mitochondria Staining Kit (JC-1), Adenosine triphosphate (ATP) assay kit and Reduced glutathione (GSH) assay kit were purchased from Sigma-Aldrich Co. Adenosine triphosphate (ATP) assay kit were purchased from Abbkine (KTB1810, Abbkine, Wuhan, China). Cellular Lipid Peroxidation Assay Kit (Liperfluo) was purchased from Shanghai Dongren Chemical Technology Co. All the used antibodies were purchased from Biolegend Co., Ltd. 4T1 cell (mouse mammary carcinoma cell) and HC11 (mouse mammary epithelial cell) were bought from American Type Culture Collection (ATCC) and cultured in Dulbecco's modified Eagle's medium (DMEM, Shanghai Zhong Qiao Xin Zhou Biotechnology Co.Ltd. China) with 10% fetal bovine serum (FBS, OriCell®FBSST-01033-500, from Cyagen Biosciences (Guangzhou) Inc,

China) at 37 °C and in a 5% CO_2 humidified air environment. Alkyne-PEG_{1000}-Fc, Alkyne-PEG_{1000}, and Alkyne-PEG_{1000}-RGD were purchased from Xi'an RuixiBiotech Co. Chemicals for organic synthesis were ordered from Shanghai Titan Scientific Co. Tetrahydrofuran (THF), and Dimethyl formamide (DMF) used for reactions were purified by solvent purification system (Innovative Technology, Inc.) before using. All air and moisture-sensitive reactions were carried out in flame-dried glassware under a nitrogen atmosphere.

1.2 General measurements

Nuclear magnetic resonance (NMR) spectra were recorded on an AVANCE NEO 500 spectrometer (Bruker, Germany) using $CDCl_3$ as the internal reference. High-resolution mass spectra (HRMS) were obtained on a Thermo Scientific Q Exactive Combined quadrupole Orbitrap mass spectrometer (Thermo Fisher Scientific Co, USA) in a Positive ion mode. Confocal laser scanning microscopy (CLSM) images were collected on Zeiss LSM880 (Zeiss, Germany). Transmission electron microscopy (TEM) images were performed on an HT7800 transmission electron microscope (Hitachi Electronics, accelerating voltage 80 kV). The size distributions of the NPs were obtained from Nano-ZS90 (Malvern, China). The size distribution of IR-FEP-RGD-S-S-S-Fc was measured by a nanometer particle size potentiometer (Malvern Nano-ZS90). The X-ray photoelectron spectroscopy (XPS) analysis was performed on K-Alpha (Thermo Fisher Scientific Co, USA). Electron spin resonance

(ESR/EPR) spectra were conducted on an EMXnano spectrometer (Bruker, Germany). Fluorescence spectra were measured on a Thermo Scientific Lumina fluorescence spectrometer (Thermo Fisher Scientific Co, USA). NIR-II fluorescence imaging was performed on AniView 30F (Guangzhou Biolight Biotechnology Co, China). Flow cytometric were conducted on a BD LSRFortessa (BD, USA). Ultra violet-visible-near infrared (UV-Vis-NIR) absorption spectra were measured by a UA-3200S spectrometer (MAPADA, China). Temperature evolution curves were tested by an infrared thermal imaging camera (Fotric 225s, China) upon irradiation with an 808 nm near-infrared (NIR) laser (0.33 W/cm^2, MDL-XF-808nm/10W, Changchun New Industries Optoelectronics Technology Co, Ltd.). The pathological sections were observed *via* Pannoramic DESK (3D HISTECH, HUN). The Near-infrared II fluorescence spectroscopy was measured *via* a Fiber optic spectrometer (NIR-17S, Shanghai ideaoptics Co, China)

1.3 Synthesis and characterization of the molecular fluorophores

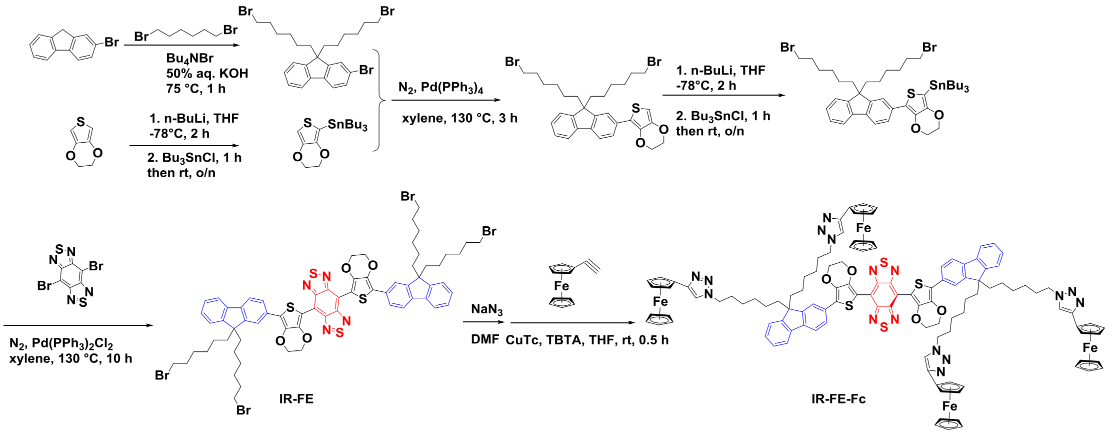

Scheme S1. Synthetic route of IR-FE and IR-FE-Fc

IR-FE and IR-FE-Fc were synthesized according to the reference.[1, 2]

IR-FE： Optical parameters in Toluene: λ_{ex} = 750 nm, λ_{em} = 995 nm, quantum yield is 31.0% (with 808 nm excitation), in CH_2Cl_2: λ_{ex} = 744 nm, λ_{em} = 1005 nm, quantum yield is 15.1% (with 808 nm excitation), in DMSO: λ_{ex} = 728 nm, λ_{em} = 1029 nm, quantum yield is 4.7% (with 808 nm excitation)

IR-FE-Fc： Optical parameters in Toluene: λ_{ex} = 760 nm, λ_{em} = 1002 nm, quantum yield is 6.52% (with 808 nm excitation), in CH_2Cl_2: λ_{ex} = 743 nm, λ_{em} = 1002 nm, quantum yield is 3.29% (with 808 nm excitation), in DMSO: λ_{ex} = 735 nm, λ_{em} = 1025 nm, quantum yield is 1.11% (with 808 nm excitation)

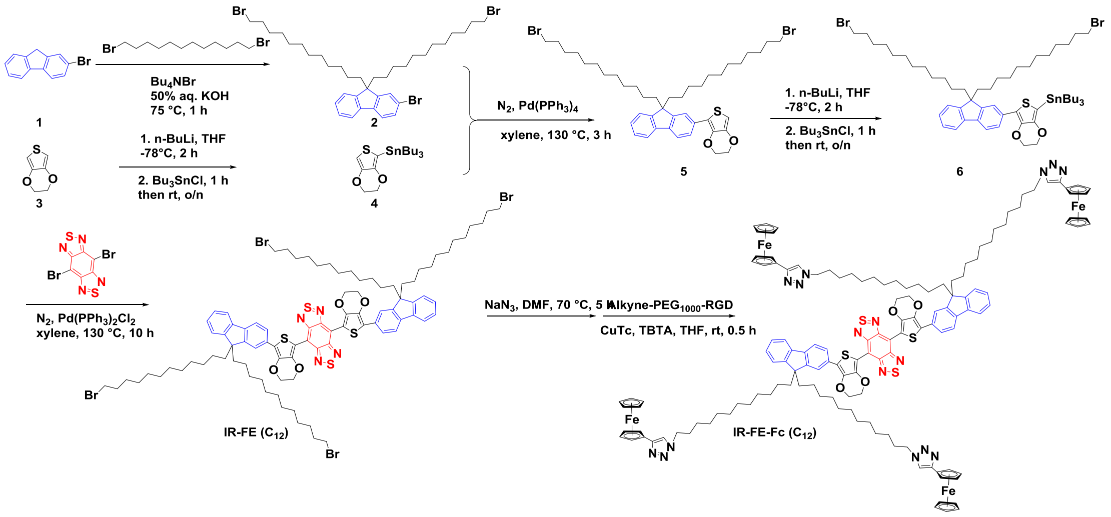

Scheme S2. Synthetic route of IR-FE-Fc (C_{12})

2-bromo-9,9-bis(12-bromododecyl)-9H-fluorene (2): To 150 ml of 45% aqueous potassium hydroxide was added 6.0 g (24.4 mmol) 2-bromofluorene, 80.3 g (0.244 mol) 1,12-dibromododecane and 0.78 g (2.4 mmol) tetrabutylammonium bromide at 75°C. The mixture was stirred for 1 h and then cooled to room temperature. The aqueous layer was extracted with dichloromethane. The organic layer was washed with 1.0 M aqueous HCl, then brine and water, and dried over anhydrous magnesium

sulfate. After removal of the solvent and the excess 1,12-dibromododecane under reduced pressure, the residue was purified by column chromatography on silica gel (eluent petroleum ether) to afford yellow, which was further purified by recrystallization by petroleum ether under -20 °C to give white solid product 13.03 g (72%). ^{1}H NMR (500 MHz, $CDCl_3$) δ 7.58 (d, *J* = 4.2 Hz, 1H), 7.47 (d, *J* = 7.9 Hz, 1H), 7.37 (d, *J* = 8.1 Hz, 2H), 7.24 (s, 3H), 3.32 (t, *J* = 6.9 Hz, 4H), 1.85 (tt, *J* = 13.7, 6.7 Hz, 4H), 1.79 – 1.70 (m, 4H), 1.34 – 1.29 (m, 4H), 1.22 – 1.07 (m, 16H), 1.01 (m,16H). ^{13}C NMR (500 MHz, $CDCl_3$). δ (ppm): 152.59, 149.93, 140.16, 140.03, 130.05, 127.59, 127.10, 126.06, 122.80, 121.11, 121.05, 119.84, 55.24, 40.11, 33.86, 32.60, 28.98, 27.74, 23.46. HRMS (ESI) calcd for $C_{37}H_{55}Br_3^{+}$, ([M $^{+}$]) 738.1827, Found 738.1874.

Tributyl(2,3-dihydrothieno[3,4-*b*][1,4]dioxin-5-yl)stannane (4): To a solution of compound **3** (2.13 g, 15.00 mmol) in THF (25.00 ml) at - 78 °C under nitrogen, *n*-BuLi solution (2.50 M in hexane, 7.20 ml, 18.00 mmol) was added dropwise. After the mixture was stirred at this temperature for another 2.0 h, tributyltin chloride (5.86 g, 18.00 mmol) was added to the solution. The reaction mixture was then slowly warmed to room temperature and stirred overnight. After that, the mixture was poured into water and extracted twice with ethyl acetate, the combined organic phase was dried with $MgSO_4$ and evaporated *in vacuo* without further purification.

5-(9,9-bis(1,2-bromohexyl)-9H-fluoren-2-yl)-2,3-dihydrothieno[3,4-*b*][1,4]dioxine (5): To a solution of compound **2** (7.39 g, 10.00 mmol) and the crude product from the previous step in xylene (100 mL) under nitrogen, $Pd(PPh_3)_4$ (1.16 g) was added. The

mixture was stirred at 130 °C for 3 h. After cooling to room temperature, the mixture was poured into water, extracted twice with ethyl acetate, dried with $MgSO_4$, and evaporated *in vacuo*. The crude product was subjected to column chromatography on silica gel to afford compound **5** as a light yellow oil (5.37 g, 67.1%). ^{1}H NMR (500 MHz, $CDCl_3$) δ 7.66 (d, *J* = 8.0 Hz, 1H), 7.59 (dd, *J* = 16.9, 9.1 Hz, 3H), 7.30 – 7.18 (m, 4H), 6.22 (d, *J* = 4.0 Hz, 1H), 4.25 (t, *J* = 8.4 Hz, 2H), 4.18 (dd, *J* = 13.2, 3.8 Hz, 2H), 3.53 – 3.32 (m, 2H), 3.30 (d, *J* = 6.9 Hz, 2H), 1.91 – 1.85 (m, 4H), 1.75 – 1.70 (m, 2H), 1.20 (m, 20H), 0.97 (m, 10H), 0.79 (m, 8H). ^{13}C NMR (500 MHz, $CDCl_3$) δ 151.13, 150.93, 142.37, 140.87, 139.73, 139.28, 137.97, 131.96, 128.83, 128.61, 126.88, 126.74, 126.04, 124.87, 124.49, 124.01, 122.84, 120.33, 119.75, 119.60, 118.39, 114.08, 97.22, 77.30, 77.05, 76.79, 64.83, 64.52, 55.12, 45.20, 40.35, 38.76, 35.01, 34.90, 34.55, 34.47, 34.06, 33.82, 32.86, 32.67, 31.96, 31.54, 31.46, 30.40, 30.22, 30.17, 30.04, 29.74, 29.70, 29.52, 29.50, 29.46, 29.43, 29.40, 29.24, 29.14, 28.94, 28.88, 28.76, 28.18, 26.89, 23.76, 23.02, 22.73, 14.16, 14.08, 13.63. HRMS (ESI) calcd for $C_{43}H_{61}O_2Br_2S^+$, ([M+H$^+$]) 801.2733, Found 801.2727.

(7-(9,9-bis(1,2-bromohexyl)-9H-fluoren-2-yl)-2,3-dihydrothieno[3,4-*b*][1,4]dioxin-5-yl) tributyl stannane (6): To a solution of compound **5** (2.40 g, 3.00 mmol) in THF (15.00 ml) at - 78 °C under nitrogen, *n*-BuLi solution (2.50 M in hexane, 1.80 mL, 4.50 mmol) was added dropwise. After the mixture was stirred at this temperature for another 2.0 h, tributyltin chloride (1.46 g, 4.50 mmol) was added to the solution. The reaction mixture was then slowly warmed to room temperature and stirred for 1.0 h. After that, the mixture was poured into water and extracted twice with ethyl acetate,

the combined organic phase was dried with $MgSO_4$ and evaporated *in vacuo* without further purification.

IR-FE (C_{12}): To a solution of compound 4,7-dibroMobenzo[1,2-c:4,5-c']bis([1,2,5]thiadiazole) BBTD (352 mg, 1.00 mmol) and the crude product from the previous step in xylene (20 ml) under nitrogen, Pd $(PPh_3)_2Cl_2$ (70 mg) was added. The mixture was stirred at 130 °C for 10 h. After cooling to room temperature, the mixture was poured into water, extracted twice with ethyl acetate, dried with $MgSO_4$, and evaporated *in vacuo*. The crude product was subjected to column chromatography on silica gel to afford IR-FE (C_{12}) as a dark green solid (950 mg, 53.0%). ^{1}H NMR (500 MHz, $CDCl_3$) δ 7.83 (s, 2H), 7.67 (s, 2H), 7.64 (s, 2H), 7.26 (t, *J* = 6.8 Hz, 8H), 4.45 (s, 4H), 4.32 (s, 4H), 3.22 (t, *J* = 6.8 Hz, 8H), 1.57 (dd, *J* = 15.6, 7.6 Hz, 22H), 1.27 (d, *J* = 7.8 Hz, 22H), 1.01 (dd, *J* = 17.3, 9.0 Hz, 22H), 0.84 (d, *J* = 7.3 Hz, 22H).^{13}C NMR (500 MHz, $CDCl_3$) δ 151.13, 150.92, 142.36, 139.72, 131.93, 126.87, 126.73, 124.85, 122.83, 120.33, 119.74, 119.58, 97.22, 77.28, 77.03, 76.77, 64.83, 64.52, 55.11, 40.34, 34.08, 32.84, 30.02, 29.72, 29.50, 29.48, 29.44, 29.38, 29.22, 28.74, 28.17, 23.75. HRMS (ESI) calcd for $C_{92}H_{118}Br_4O_4N_4S_4^+$, ([M+H$^+$]) 1789.4821, Found 1789.4830. Optical parameters in Toluene: λ_{ex} = 760 nm, λ_{em} = 985 nm, quantum yield is 29.06% (with 808 nm excitation), in CH_2Cl_2: λ_{ex} = 747 nm, λ_{em} = 1002 nm, quantum yield is 14.4% (with 808 nm excitation), in DMSO: λ_{ex} = 749 nm, λ_{em} = 1027 nm, quantum yield is 4.04% (with 808 nm excitation)

IR-FE-Fc (C_{12}): Compound IR-FE(C_{12}) (123 mg, 0.069 mmol) and sodium azide (47 mg, 0.72 mmol) were dissolved in DMF (10 mL) and heated for 3 h at 70°C. After that, a large amount of water was added and stirred until all solids dissolved. Then it was extracted twice with ethyl acetate, and the combined organic phase was dried with $MgSO_4$ and evaporated *in vacuo*. The crude product was subjected to flash column chromatography (PE/EA = 2) on silica gel to afford a dark green solid (93 mg, 0.071 mmol). The dark green solid was dissolved in 5 mL THF and copper(I) thiophene-2-carboxylate (CuTc) (10 mg), ethynylferrocene (62 mg), and tris[(1-benzyl-1H-1,2,3-triazol-4-yl) methyl] amine (TBTA) (5 mg) was added. The system was stirred at RT for 0.5 h, then filtered with diatomite. The solvent was evaporated *in vacuo*. The crude product was subjected to column chromatography on silica gel with (PE/EA = 2) and recrystallized by methyl tert-butyl ether to afford IR-FE-Fc (C_{12}) as a dark green solid (88.6 mg, 51.80%). ^1H NMR (500 MHz, $CDCl_3$) δ 7.91 (d, *J* = 7.9 Hz, 2H), 7.75 – 7.68 (m, 6H), 7.34 – 7.28 (m, 10H), 4.67 (s, 8H), 4.49 (s, 4H), 4.36 (s, 4H), 4.22 (dd, *J* = 18.3, 11.2 Hz, 16H), 4.03 (s, 20H), 2.34 (t, *J* = 7.1 Hz, 20H), 1.64 (dd, *J* = 15.0, 7.4 Hz, 24H), 1.54 – 1.39 (m, 24H), 0.95 (d, *J* = 7.3 Hz, 20H). ^{13}C NMR (500 MHz, $CDCl_3$) δ 155.07, 152.63, 151.68, 150.11, 148.12, 147.65, 147.33, 147.11, 146.32, 141.94, 141.26, 140.79, 140.00, 138.55, 137.29, 136.11, 132.87, 132.25, 131.32, 127.59, 127.37, 127.20, 126.52, 126.12, 125.82, 124.76, 124.50, 124.02, 122.86, 121.05, 120.78, 120.23, 120.05, 119.91, 119.12, 117.47, 108.91, 87.24, 77.34, 77.08, 76.83, 72.42, 64.76, 64.61, 59.51, 54.68, 54.28, 54.14, 50.70, 45.47, 43.47, 40.54, 38.78, 36.18, 34.91, 34.64, 34.56, 34.40, 33.05,

31.97, 31.81, 31.55, 31.48, 30.86, 30.36, 30.23, 29.99, 29.75, 29.41, 28.99, 27.37, 27.23, 24.12, 23.77, 22.74, 22.61, 18.54, 14.19, 14.11, 1.24. HRMS (ESI) calcd for $C_{140}H_{159}Fe_4N_{16}O_4S_4^+$, ([M+H$^+$]) 2479.9005, Found 2479.9040. Optical parameters in Toluene: λ_{ex} = 758 nm, λ_{em} = 999 nm, quantum yield is 16.9% (with 808 nm excitation), in CH_2Cl_2: λ_{ex} = 743 nm, λ_{em} = 1002 nm, quantum yield is 6.81% (with 808 nm excitation), in DMSO: λ_{ex} = 750 nm, λ_{em} = 1029 nm, quantum yield is 2.98% (with 808 nm excitation)

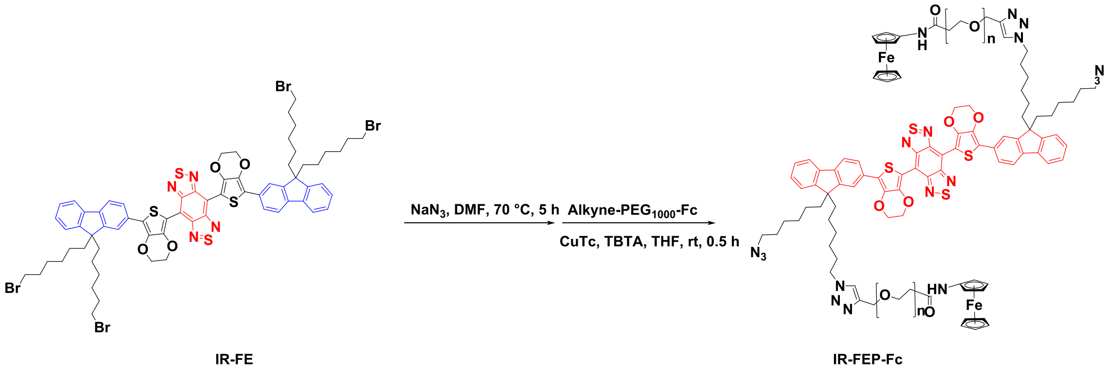

Scheme S3. Synthetic route of IR-FEP-Fc

IR-FEP-Fc: Compound IR-FE (100 mg, 0.069 mmol) and sodium azide (47 mg, 0.72 mmol) were dissolved in DMF (10 mL) and heated for 5 h at 70°C. After that, a large amount of water was added and stirred until all solids dissolved. Then it was extracted twice with ethyl acetate, and the combined organic phase was dried with $MgSO_4$ and evaporated *in vacuo*. The crude product was subjected to flash column chromatography (PE/EA = 2) on silica gel to afford a dark green solid (93 mg, 0.071 mmol). The dark green solid was dissolved in 5 mL THF and copper(I) thiophene-2-carboxylate (CuTc) (10 mg), alkyne-PEG$_{1000}$-Fc ($Mw \approx$ 1000) (280 mg), and tris[(1-benzyl-1H-1,2,3-triazol-4-yl) methyl] amine (TBTA) (5 mg) were added.

The system was stirred at RT for 0.5 h, then filtered with diatomite. The solvent was evaporated *in vacuo*. The crude product was subjected to column chromatography on silica gel and recrystallized by methyl tert-butyl ether to afford IR-FEP-Fc as a dark green solid (117 mg, 51.80%). ^{1}H NMR (500 MHz, $CDCl_3$) δ 7.84 (d, *J* = 8.0 Hz, 2H), 7.70 – 7.64 (m, 6H), 7.27 (t, *J* = 5.7 Hz, 8H), 4.41 (dd, *J* = 21.4, 17.0 Hz, 12H), 4.18 (s, 10H), 3.90 (dd, *J* = 5.3, 1.9 Hz, 8H), 3.60 (s, 200H), 3.04 (s, 8H), 2.78 (m, *J* = 11.3 Hz, 30H), 1.89 (s, 12H), 1.59 (m, *J* = 14.2, 6.9 Hz, 10H), 1.19 (m, 12H), 0.77 (m, *J* = 3.7 Hz, 10H). ^{13}C NMR (500 MHz, $CDCl_3$) δ 168.57, 151.66, 130.38, 77.29, 77.03, 76.78, 70.88, 70.58, 70.28, 69.49, 68.35, 60.41, 34.05, 32.64, 29.04, 27.77, 25.65, 25.56, 25.48, 20.19, 14.21. Optical parameters in Toluene: λ_{ex} = 758 nm, λ_{em} = 995 nm, quantum yield is 19.94% (with 808 nm excitation), in CH_2Cl_2: λ_{ex} = 743 nm, λ_{em} = 1009 nm, quantum yield is 8.86% (with 808 nm excitation), in DMSO: λ_{ex} = 749 nm, λ_{em} = 1022 nm, quantum yield is 2.44% (with 808 nm excitation)

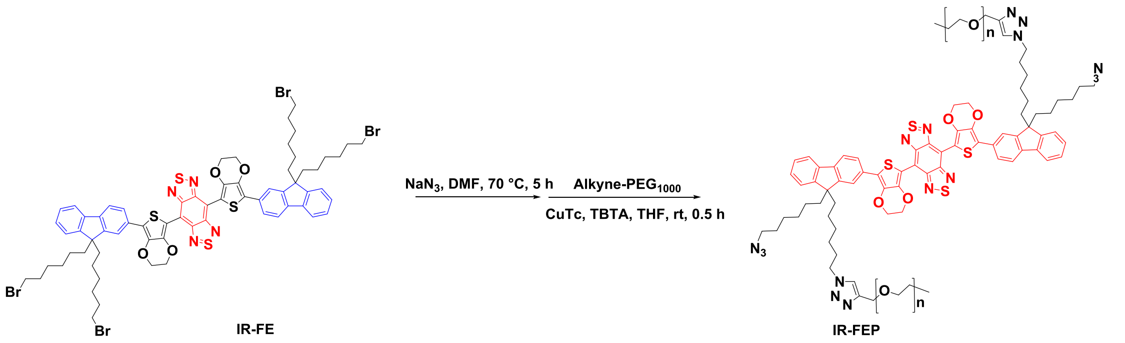

Scheme S4. Synthetic route of IR-FEP

IR-FEP: Compound IR-FE (100 mg, 0.069 mmol) and sodium azide (47 mg, 0.72 mmol) were dissolved in DMF (10 mL) and heated for 5 h at 70°C. After that, a large amount of water was added and stirred until all solids dissolved. Then it was extracted

twice with ethyl acetate, and the combined organic phase was dried with $MgSO_4$ and evaporated *in vacuo*. The crude product was subjected to flash column chromatography (PE/EA = 2) on silica gel to afford a dark green solid (93 mg, 0.071 mmol). The dark green solid was dissolved in 5 mL THF and copper(I) thiophene-2-carboxylate (CuTc) (10 mg), alkyne-PEG_{1000} (171 mg), and tris[(1-benzyl-1H-1,2,3-triazol-4-yl) methyl] amine (TBTA) (5 mg) was added. The system was stirred at RT for 0.5 h, then filtered with diatomite. The solvent was evaporated *in vacuo*. The crude product was subjected to column chromatography on silica gel and recrystallized by methyl tert-butyl ether to afford IR-FEP as a dark green solid (114.6 mg, 60.1%). ^{1}H NMR (500 MHz, $CDCl_3$) δ 7.84 (d, *J* = 7.9 Hz, 2H), 7.66 (dd, *J* = 16.4, 7.9 Hz, 6H), 7.27 (t, *J* = 5.5 Hz, 8H), 4.40 – 4.38 (m, 4H), 4.32 (s, 4H), 3.70 (dd, *J* = 18.2, 14.4 Hz, 8H), 3.58 (s, 179H), 3.03 (s, 4H), 2.82 – 2.74 (m, 18H), 1.76 (s, 12H), 1.59 (dt, *J* = 14.3, 7.2 Hz, 10H), 1.14 (dd, *J* = 15.3, 7.9 Hz, 12H), 0.85 – 0.73 (m, 10H). ^{13}C NMR (500 MHz, $CDCl_3$) δ 152.59, 146.09, 144.91, 144.58, 140.80, 129.12, 128.07, 123.77, 122.43, 77.31, 77.05, 76.80, 72.59, 70.50, 70.41, 70.07, 69.62, 64.60, 61.46, 55.08, 50.23, 31.44, 30.11, 29.70, 29.28, 26.14, 23.54, 14.13, 9.54. Optical parameters in Toluene: λ_{ex} = 757 nm, λ_{em} = 985 nm, quantum yield is 20.56% (with 808 nm excitation), in CH_2Cl_2: λ_{ex} = 743 nm, λ_{em} = 1009 nm, quantum yield is 9.55% (with 808 nm excitation), in DMSO: λ_{ex} = 749 nm, λ_{em} = 1025 nm, quantum yield is 2.54% (with 808 nm excitation), in H_2O: λ_{ex} = 775nm, λ_{em} = 1035 nm, quantum yield is 2.25% (with 808 nm excitation)

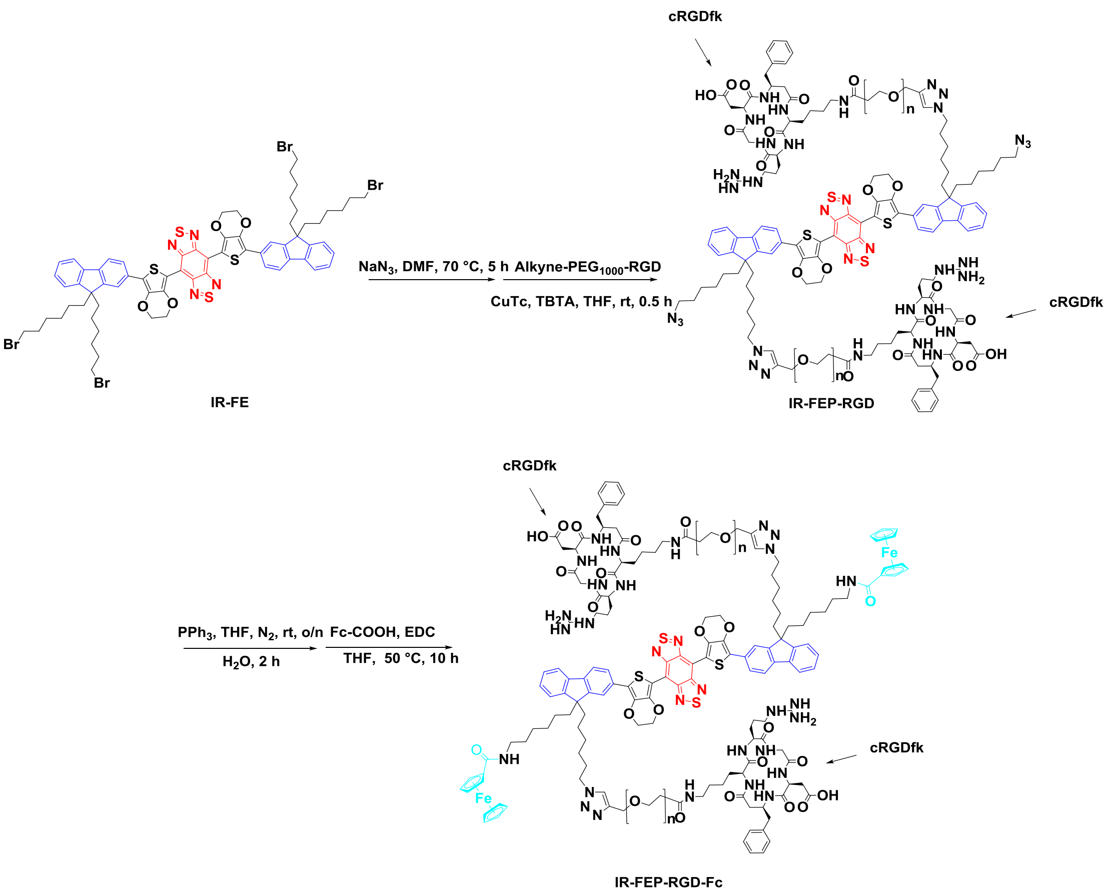

Scheme S5. Synthetic route of IR-FEP-RGD-Fc

IR-FEP-RGD: Compound IR-FE (100 mg, 0.069 mmol) and sodium azide (47 mg, 0.72 mmol) were dissolved in DMF (10 mL) and heated for 5 h at 70°C. After that, a large amount of water was added and stirred until all solids dissolved. Then it was extracted twice with ethyl acetate, and the combined organic phase was dried with $MgSO_4$ and evaporated *in vacuo*. The crude product was subjected to flash column chromatography (PE/EA = 2) on silica gel to afford a dark green solid (93 mg, 0.071 mmol). The dark green solid was dissolved in 5 mL THF and copper(I) thiophene-2-carboxylate (CuTc) (10 mg), alkyne-PEG$_{1000}$-RGD (280 mg), and tris[(1-benzyl-1H-1,2,3-triazol-4-yl) methyl] amine (TBTA) (5 mg) was added. The system was stirred at RT for 0.5 h, then filtered with diatomite. The solvent was

evaporated *in vacuo*. The crude product was subjected to column chromatography on silica gel with (PE/EA = 2) and recrystallized by methyl tert-butyl ether to afford IR-FEP-RGD as a dark green solid (130.2 mg, 57.20%). ^{1}H NMR (500 MHz, $CDCl_3$) δ 7.88 – 7.76 (m, 2H), 7.68 – 7.62 (m, 6H), 7.26 (dd, *J* = 11.5, 7.0 Hz, 8H), 5.21 (d, *J* = 18.9 Hz, 5H), 4.39 (d, *J* = 3.6 Hz, 4H), 4.18 (s, 4H), 3.90 (d, *J* = 3.6 Hz, 10H), 3.71 (d, *J* = 4.1 Hz, 8H), 3.62 – 3.58 (m, 199H), 1.85 (s, 12H), 1.60 – 1.56 (m, 10H), 1.25 – 1.22 (m, 12H), 0.83 (m, *J* = 5.6 Hz, 10H). ^{13}C NMR (500 MHz, $CDCl_3$) δ 167.54, 121.80, 76.26, 76.01, 75.75, 69.87, 69.57, 69.26, 68.47, 67.34, 33.00, 31.62, 28.56, 28.24, 28.02, 26.75, 24.54, 24.47, 22.52, 12.58, 0.00.

IR-FEP-RGD-Fc: A Schlenk bottle was sealed after a magnetic stir bar, IR-FEP-RGD (40 mg) and triphenylphosphine (20 mg) were added, then the gas in the bottle was replaced with inert gas (N_2) three times. The 2 mL degassed THF was injected into the Schlenk bottle, and the reaction was stirred at room temperature overnight, then the bottle was put into the ice-water bath for 5 min, and 0.1 mL of cold water was dropwise to the reaction, then kept reaction at ice-water bath for 2 h. The IR-FEP-RGD-NH_2 (25.7 mg) was obtained after being washed with 30 mL of MTBE three times as a vicious green solid, and a freeze dryer completely removed the residual water, then directly used for the next step without further purification. To a solution of IR-FEP-RGD-NH_2 which was obtained from the previous step in THF (2 mL), then a solution of Fc-COOH (6.5 mg) in THF (5 mL) was dropwise added. And add 1-(3-Dimethylaminopropyl)-3-ethylcarbodiimide (EDC) (10mg) to the reaction. The reaction was stirred for 10 h. The liposoluble organic impurities can be removed by washing with tert-Butyl methyl ether (MTBE) three times, and the water-soluble impurities can be removed by washing with water in Amicon® ultra 15 mL centrifugal filters 10 times. At last, the IR-FEP-RGD-Fc was obtained by freeze-drying the previous aqueous solution as 18.6 mg of green solid. ^{1}H NMR (500 MHz, $CDCl_3$) δ 7.85 (dd, *J* = 7.9, 1.5 Hz, 2H), 7.68 – 7.64 (m, 6H), 7.27 (t, *J* = 5.8 Hz,

8H), 4.44 – 4.32 (m, 16H), 4.15 (s, 8H), 3.58 (s, 207H), 1.72 (s, 20H), 1.37 – 1.32 (m, 10H), 1.07 (d, J = 13.6 Hz, 10H). ^{13}C NMR (126 MHz, $CDCl_3$) δ 152.62, 148.05, 141.96, 102.48, 82.61, 80.35, 77.30, 77.05, 76.80, 75.54, 73.53, 72.35, 71.76, 71.25, 70.55, 70.06, 69.87, 69.05, 68.73, 68.26, 63.84, 62.46, 55.11, 54.03, 51.38, 50.25, 45.70, 45.06, 40.27, 38.33, 31.94, 30.95, 29.71, 29.43, 28.89, 28.68, 27.57, 26.32, 26.08, 23.58, 23.27, 22.71, 19.24, 14.15, 10.55, 8.55, -4.72.

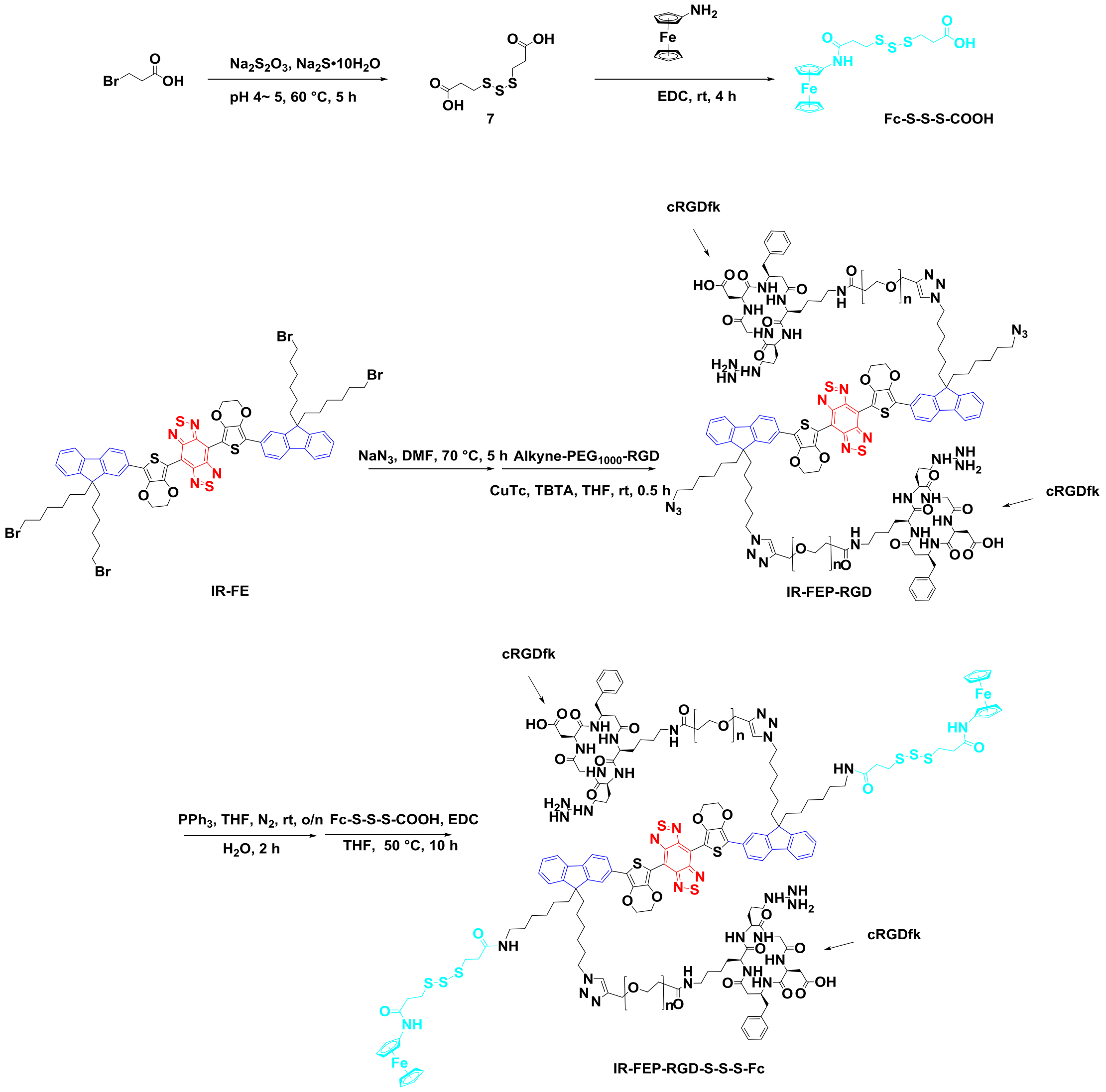

Scheme S6. Synthetic route of IR-FEP-RGD and IR-FEP-RGD-S-S-S-Fc

3,3'-trisulfanediyldipropionic acid (7) was synthesized according to the reference.[3]

^{1}H NMR (500 MHz, DMSO) δ 12.35 (s, 2H), 2.88 (t, J = 6.9 Hz, 4H), 2.62 (t, J = 6.9

Hz, 4H).^{13}C NMR (500 MHz, DMSO) δ 173.12, 40.49, 40.32, 40.15, 39.99, 39.82, 39.65, 39.48, 34.04, 33.48. HRMS (ESI) calcd for $C_6H_9O_4S_3^-$ ([M–H$^+$]) 240.9668, Found 240.9665.

Fc-S-S-S-COOH: after mixing Aminoferrocene (33 mg, 0.164 mmol) and compound **7** (40 mg, 0.165 mmol) in THF (15 mL), a solution of EDC (40 mg) was dropwise added. The reaction was stirred for 4 h. Then the mixture was poured into water, extracted twice with ethyl acetate, dried with $MgSO_4$, and evaporated *in vacuo*. The crude product was subjected to column chromatography on silica gel to afford Fc-S-S-S-COOH as a brownish-yellow solid (41.6mg 59.3%). ^{1}H NMR (500 MHz, $CDCl_3$) δ 7.66 (d, *J* = 44.7 Hz, 1H), 4.65 (s, 1H), 4.22 (d, *J* = 34.1 Hz, 6H), 4.02 (s, 2H), 3.04 – 2.94 (m, 4H), 2.77 – 2.64 (m, 4H). ^{13}C NMR (500 MHz, $CDCl_3$) δ 174.44, 172.74, 169.32, 77.34, 77.09, 76.84, 69.58, 69.43, 67.93, 67.01, 66.75, 66.62, 65.92, 64.67, 62.09, 61.49, 39.98, 39.81, 39.64, 36.33, 34.06, 33.84, 33.66, 32.43, 31.44, 30.71, 30.19. HRMS (ESI) calcd for $C_{16}H_{18}FeNO_3S_3^-$, ([M–H$^+$]) 423.9803, Found 423.9802.

IR-FEP-RGD: Compound IR-FE (100 mg, 0.069 mmol) and sodium azide (47 mg, 0.72 mmol) were dissolved in DMF (10 mL) and heated for 5 h at 70°C. After that, a large amount of water was added and stirred until all solids dissolved. Then it was extracted twice with ethyl acetate, and the combined organic phase was dried with $MgSO_4$ and evaporated *in vacuo*. The crude product was subjected to flash column chromatography (PE/EA = 2) on silica gel to afford a dark green solid (93 mg, 0.071 mmol). The dark green solid was dissolved in 5 mL THF and copper(I)

thiophene-2-carboxylate (CuTc) (10 mg), alkyne-PEG_{1000}-RGD (280 mg), and tris[(1-benzyl-1H-1,2,3-triazol-4-yl) methyl] amine (TBTA) (5 mg) was added. The system was stirred at RT for 0.5 h, then filtered with diatomite. The solvent was evaporated *in vacuo*. The crude product was subjected to column chromatography on silica gel with (PE/EA = 2) and recrystallized by methyl tert-butyl ether to afford IR-FEP-RGD as a dark green solid (130.2 mg, 57.20%). ^{1}H NMR (500 MHz, $CDCl_3$) δ 7.88 – 7.76 (m, 2H), 7.68 – 7.62 (m, 6H), 7.26 (dd, *J* = 11.5, 7.0 Hz, 8H), 5.21 (d, *J* = 18.9 Hz, 5H), 4.39 (d, *J* = 3.6 Hz, 4H), 4.18 (s, 4H), 3.90 (d, *J* = 3.6 Hz, 10H), 3.71 (d, *J* = 4.1 Hz, 8H), 3.62 – 3.58 (m, 199H), 1.85 (s, 12H), 1.60 – 1.56 (m, 10H), 1.25 – 1.22 (m, 12H), 0.83 (m, *J* = 5.6 Hz, 10H). ^{13}C NMR (500 MHz, $CDCl_3$) δ 167.54, 121.80, 76.26, 76.01, 75.75, 69.87, 69.57, 69.26, 68.47, 67.34, 33.00, 31.62, 28.56, 28.24, 28.02, 26.75, 24.54, 24.47, 22.52, 12.58, 0.00.

IR-FEP-RGD-S-S-S-Fc: A Schlenk bottle was sealed after a magnetic stir bar, IR-FEP-RGD (40 mg) and triphenylphosphine (20 mg) were added, then the gas in the bottle was replaced with inert gas (N_2) three times. The 2 mL degassed THF was injected into the Schlenk bottle, and the reaction was stirred at room temperature overnight, then the bottle was put into the ice-water bath for 5 min, and 0.1 mL of cold water was dropwise to the reaction, then kept reaction at ice-water bath for 2 h. The IR-FEP-RGD-NH_2 (25.7 mg) was obtained after being washed with 30 mL of MTBE three times as a vicious green solid, and a freeze dryer completely removed the residual water, then directly used for the next step without further purification. To a solution of IR-FEP-RGD-NH_2 which was obtained from the previous step in THF (2

mL), then a solution of Fc-S-S-S-COOH (12 mg) in THF (5 mL) was dropwise added. And add 1-(3-Dimethylaminopropyl)-3-ethylcarbodiimide (EDC) (10mg) to the reaction. The reaction was stirred for 10 h. The liposoluble organic impurities can be removed by washing with tert-Butyl methyl ether (MTBE) three times, and the water-soluble impurities can be removed by washing with water in Amicon® ultra 15 mL centrifugal filters 10 times. At last, the IR-FEP-RGD-S-S-S-Fc was obtained by freeze-drying the previous aqueous solution as 21.8 mg of green solid. ^{1}H NMR (500 MHz, $CDCl_3$) δ 7.84 (d, *J* = 8.0 Hz, 2H), 7.66 (dd, *J* = 14.9, 6.4 Hz, 6H), 7.30 – 7.23 (m, 8H), 5.23 (s, 6H), 4.39 (t, *J* = 34.9 Hz, 16H), 4.18 (s, 10H), 3.90 (s, 10H), 3.58 (s, 214H), 2.78 (d, *J* = 10.9 Hz, 22H), 1.98 (m, 10H), 1.56 (m, *J* = 7.4 Hz, 10H), 1.26 (m, 10H), 0.85 (m, 10H). ^{13}C NMR (500 MHz, $CDCl_3$) δ 168.58, 128.81, 77.29, 77.03, 76.78, 71.50, 70.88, 70.67, 70.64, 70.57, 70.27, 69.48, 68.35, 64.35, 61.13, 34.04, 32.63, 31.44, 30.75, 30.20, 29.04, 27.77, 26.83, 25.65, 25.56, 25.47, 20.19, 14.21, 13.61. Optical parameters in H_2O: λ_{ex} = 774 nm, λ_{em} = 1029 nm, quantum yield is 0.08% (with 808 nm excitation) after adding in GSH 1 h quantum yield is 1.84% (with 808 nm excitation).

1.4 Photothermal conversion performance of IR-FEP-RGD-S-S-S-Fc

A series of IR-FEP-RGD-S-S-S-Fc aqueous solutions (1 mL) with concentrations from 10~50 μM were subjected to laser irradiation for 10 min, respectively. Then, the temperature was monitored by a thermal imager, respectively. After the laser on/off test, the following equation determined the photothermal conversion efficiency (PCE, η) of IR-FEP-RGD-S-S-S-Fc.

$$\eta = \frac{hS(T_{max} - T_{surr}) - Q_0}{I(1 - 10^{-A_\lambda})} \quad (1)$$

$$hS = \frac{\sum m_i C_{P.i}}{\tau_s} \quad (2)$$

$$\tau_s = \frac{\mathrm{t}}{-\ln\theta} \quad (3)$$

$$\theta = \frac{\mathrm{T} - T_{surr}}{T_{max} - T_{surr}} \quad (4)$$

$$Q_0 = \mathrm{h}(T_{max} - T_{surr}) \quad (5)$$

where h is the heat transfer coefficient, S represents the sample container surface area, A is the surface area of the container, T_{max} represents the steady-state maximum temperature, T_{surr} represents the ambient room temperature, Q_0 is the heat associated with the light absorbance of the solvent, which is measured using pure water, I is the laser power, and A_λ is the absorbance of IR-FEP-RGD-S-S-S-Fc at the wavelength of 808 nm.

1.5 Measurement of quantum yield.

The fluorescence quantum yields of the fluorophores were measured according to the reference.[1, 4] A Fiber optic spectrometer (NIR-17S, one 900-nm long-pass filter (Thorlabs) was used as emission filter) was used to measure the fluorescence spectra, which under an 808 nm laser excitation (MDL-XF-808nm, 160 mW) in the region of 900-1800 nm. The fluorescence quantum yield was determined against the reference fluorophore IR-FE (QY=31% in toluene solutions) and IR-FEP (QY=2.0% in aqueous solution) [1]. Reference fluorophore IR-FE and IR-FEP were used with a concentration of optical density (OD) of 0.08 at 808 nm. Their NIR-II fluorescence emission

intensities were measured under the same 808 nm excitation. Using the measured optical density (OD) at 808 nm and spectrally integrated fluorescence intensity (F), the quantum yield of the test sample can be calculated according to the following equation:

$$\Phi_x(\lambda) = \Phi_{st}(\lambda) \times \frac{F_x}{F_{st}} \times \frac{A_{st}(\lambda)}{A_x(\lambda)}$$
$$= \Phi_{st}(\lambda) \times \frac{F_x}{F_{st}} \times \frac{1 - 10^{-OD_{st}(\lambda)}}{1 - 10^{-OD_x(\lambda)}}$$

1.6 Chemodynamic activity of IR-FEP-RGD-S-S-S-Fc.

The •OH generation was determined by measuring the TMB oxidation by IR-FEP-RGD-S-S-S-Fc and H_2O_2 with an absorption peak at 652 nm. Typically, a mixture of 3,3',5,5'-tetramethyl-benzidine (TMB) (1 μL, 0.1 M) and IR-FEP-RGD-S-S-S-Fc aqueous solution (150 μL, 50 μM) was kept still in the dark for 1 h. Next, H_2O_2 (5 μL, 30%) solution was introduced (the final volume is 2.0 mL). In addition, the effects of GSH and laser irradiation on the •OH generation were also explored. After adding GSH (10 mM), the above solution was determined or irradiated by an 808 nm laser for 10 min.

The ESR tests were conducted as follows: 90 μL of H_2O_2 (30%) and 100 μL of IR-FEP-RGD-S-S-S-Fc (100 μM) were mixed with 10 μL of 5, 5-dimethyl-1-pyrroline N-oxide (DMPO). The ESR spectra were recorded after 10

min. The NIR irradiation for 10 min was attached to investigate its effect on the generation of •OH.

1.7 The generation of intracellular •OH and lipid peroxide (LPO)

Intracellular •OH generation and lipid peroxide (LPO) were measured *via* APF and Liperfluo, respectively. The cells were incubated at 37 °C with 5% CO_2 in DMEM containing 10% fetal bovine serum and 1% penicillin-streptomycin under a humidified atmosphere. The cells were divided into six experimental groups: PBS, PBS + L, IR-FEP-RGD (50 μM), IR-FEP-RGD + L, IR-FEP-RGD-S-S-S-Fc (50 μM), IR-FEP-RGD-S-S-S-Fc + L. After different treatments, APF or Liperfluo was added into 4T1 cells for 20 min. After that, the cells were transferred to confocal laser scanning microscopy or flow cytometry to obtain the intracellular •OH and LPO levels.

1.8 Controlled release of H_2S and intracellular H_2S detection

The release of H_2S from IR-FEP-RGD-S-S-S-Fc (50 μM) dispersion was measured at different time points (0, 2, 4, 8, 12, and 24 min) after treating with/without laser irradiation or GSH (5 mM) incubation via Washington State Probe 1 (WSP-1), and quantitated using a microplate reader (λ_{ex} = 485/20 nm, λ_{em} = 528/20 nm).

Before intracellular H_2S were measured, the 4T1 cells were divided into six groups: PBS, PBS + L, IR-FEP-RGD (50 μM), IR-FEP-RGD + L, IR-FEP-RGD-S-S-S-Fc (50 μM), IR-FEP-RGD-S-S-S-Fc + L. After different treatments, WSP-1 was added into 4T1 cells for incubation of 20 min. Then, the fluorescence images of all groups were captured with confocal laser scanning microscopy.

1.9 Tumor cell targeting assays

The IR-FEP, IR-FEP-RGD, and IR-FEP-RGD-S-S-S-Fc PBS solution (50 μM, 2mL) was co-incubation with 4T1 cells (Cyclic-RGDfK (cRGDfk) peptide was added as a blocking group and HC11 cells were used as normal cells for control). After 1 h, the cells were washed thoroughly with PBS and analyzed by confocal laser scanning microscopy.

The IR-FEP, IR-FEP-RGD, and IR-FEP-RGD-S-S-S-Fc (50 μM, 2mL) were co-incubated with 4T1 or HC11 cells for flow cytometry analysis. After PBS washing, the cells were digested with trypsin−ethylenediaminetetraacetate (0.05%) for 3 min. Then the cells were centrifuged and washed with PBS carefully. After that, cells were analyzed by flow cytometry (BD LSRFortessa, USA).

Alternatively, the NIR-II fluorescence imaging system was used to verify cell targeting. IR-FEP-RGD-S-S-S-Fc (50 μM) was first incubated with 4T1 cells, HC11 cells, 4T1 cells+1 mM NEM, and 4T1 cells+500 μM cRGDfk (used for blocking αvβ3), and was then subjected to fluorescence semi-quantitative analysis.

1.10 Evaluation of cytochrome c oxidase (COX IV)

The COX IV expression in 4T1 cells after different treatments was evaluated by the immunofluorescence staining. Typically, the cells were incubated with our samples (50 μM) for 12 h. After that, the cells were fixed with 4% PFA at 4 °C. After 20 min, the cells were treated with 0.2% Triton X-100-PBS for 5 min. Then, BSA-PBS was added to block the cells for 1 h. Finally, the cells were stained with COX IV-related

primary antibody, secondary antibody, and Hoecht 33258. for fluorescence imaging (Ex = 488 nm).

1.11 Intracellular and extracellular GSH quantification

The 4T1 cells seeded in a 6-well plate (1×10^6 cells/ well) were cultured in RPMI1640 supplemented with 10% fetal bovine serum (FBS, Gibco) under a sterile environment (5% CO_2, 37°C, penicillin (100 U/mL), and 100 μg/mL of streptomycin) for 24 h. The incubation media were replaced with fresh ones before further treatments. The cells were treated with different conditions (PBS, PBS + L, IR-FEP-RGD (50 μM), IR-FEP-RGD + L, IR-FEP-RGD-S-S-S-Fc (50 μM), IR-FEP-RGD-S-S-S-Fc + L). After 8 h, the cells were collected by centrifugation at 1500 rpm for 15 min at 4 °C. Then, the redispersed cells were frozen in liquid nitrogen and thawed at 37 °C. The as-obtained samples were centrifuged at 10000 rpm for 10 min at 4 °C. The GSH and GSSH were finally detected quantitatively by the DTNB assay kit with a UV-vis spectrophotometer.[5]

For extracellular GSH quantification, IR-FEP-RGD-S-S-S-Fc (50 μM) was incubated with GSH (10 mM), and the total volume of the system was 200 μL in a shaken mode at 37 °C for 0, 10, 20, 30, 40, 50, and 60 min under the dark. After that, DTNB (5,5'-dithiobis-(2-nitrobenzoic acid)) was introduced into the above solution. The absorption at 412 nm was monitored with a UV-vis spectrophotometer.

1.12 Investigation of the mitochondrial membrane potential

The 4T1 cells ($1*10^5$/well) were cultured RPMI1640 supplemented with 10% fetal bovine serum (FBS, Gibco) under a sterile environment (5% CO_2, 37°C, penicillin

(100 U/mL), and 100 μg/mL of streptomycin) for overnight. The incubation media were replaced with fresh ones before further treatments. The cells were treated with different conditions (PBS, PBS + L, IR-FEP, IR-FEP + L, IR-FEP-RGD, IR-FEP-RGD + L, IR-FEP-RGD-S-S-S-Fc, IR-FEP-RGD-S-S-S-Fc + L, Concentration: 50 μM). After 4 h, JC-1 dye was incubated with the cells for mitochondrial staining by CLSM (Zeiss LSM880, Germany).

1.13 Cell viability assay

The cytotoxicity was evaluated by the Cell Counting Kit-8 (CCK8) Assay. Typically, PBS, IR-FEP (50 μM), IR-FEP-RGD (50 μM), and IR-FEP-RGD-S-S-S-Fc (50 μM) (in DMEM) were introduced to different wells containing the cells, respectively. After 6 h, the original medium was discarded and 200 μL of the fresh medium was added. After 1 h, the cells were subjected to laser irradiation for 10 min (808 nm, 0.33W/cm^2) before another incubation for 10 h. Then, the viability of cells was assessed by measuring the absorbance at 450 nm using a Biotek/800TS (Berten, USA). For flow cytometric analysis (BD LSRFortessa, USA), after laser irradiation and incubation for 10 h, the cells were collected and stained with an Annexin V/PI cell assay kit (Invitrogen). For cell live/dead imaging, after laser irradiation and incubation for 10 h, the cells were stained with Calcein-AM/PI and imaged by CLSM (Zeiss LSM880, Germany).

1.14 Animal tumor models

All animal experiments follow the Institutional Animal Use and Care Regulations at the University of South China. The specific pathogen-free female BALB/c mice (6-8

weeks old, approximately 20 g) were employed for establishing the subcutaneous tumor models by injecting 4T1 cells (1.0×10^7 cells/mL) into the selected positions of BALB/c mice. The whole therapeutic process was initiated when the tumor volume reached 130 mm^3.

1.15 *In vivo* NIR-II fluorescence and thermal imaging

IR-FEP, IR-FEP-RGD, and IR-FEP-RGD-S-S-Fc (50 μM, 100 μL) were injected intravenously into 4T1 tumor-bearing mice, respectively. Then, the treated mice were then imaged by an AniView 30F machine (0-12 h) with a fiber-coupled 808-nm diode laser (RMPC, power density: 140 mW/cm^2) as excitation light. The emission light was collected by the 900-nm long-pass filter (Thorlabs) to filter the emitted light and focus on the detector.

Major organs were collected after the mice were euthanized and imaged. The tumor temperature monitor of the mice was carried out with an 808 nm laser irradiation for 10 min at 12 h after injection. The temperature data and corresponding thermal images were recorded by an infrared thermography equipment.

1.16 Pharmacokinetics, biodistribution, and excretion of IR-FEP-RGD-S-S-S-Fc

The healthy mice were injected with IR-FEP-RGD-S-S-S-Fc (50 μM, 100 μL) intravenously to explore the pharmacokinetics, biodistribution, and excretion of IR-FEP-RGD-S-S-S-Fc. The mice blood and feces solutions were obtained for fluorescence analysis at regular intervals at 1030 nm after adding 10 mM GSH to study the blood concentration, hepatic excretion, and distribution of

IR-FEP-RGD-S-S-S-Fc (Their feces and all organs were homogenized and ground). The calculations for the pharmacokinetics, biodistribution, and excretion of IR-FEP-RGD-S-S-S-Fc were performed according to serval previous works [1, 2].

1.17 Antitumor therapy *in vivo*

Tumor-bearing mice were divided into eight groups and, respectively, treated with intravenous administration in different conditions: I: PBS − L, II: PBS + L, III: IR-FEP − L, IV: IR-FEP + L (50 μM, 100 μL), V: IR-FEP-RGD − L, VI: IR-FEP-RGD + L (50 μM, 100 μL), VII: IR-FEP-RGD-S-S-S-Fc − L, VIII: IR-FEP-RGD-S-S-S-Fc + L (50 μM, 100 μL), and + L, and − L were present as 808 nm laser/non-laser irradiation. After 12 h, the tumor site of mice in the laser irradiation groups were subjected to an 808 nm laser irradiation at a power of 0.33 W/cm^2 for 10 min. The therapeutic effects of all mice were monitored by measuring the tumor size based on the equation of $V = (L \times W^2)/2$ (L for length and W for width of tumor). The body weights of all mice were recorded every other day. After 14 days, the tumor or normal tissues were sliced and stained with Hematoxylin-Eosin (H&E), TdT-mediated dUTP-biotin nick end labeling (TUNEL), cleaved caspase-3, GPX4, and heat shock protein. 70 (HP70), Phosphorylated H2A histone family member X (γ-H2AX), and Ki-67, respectively.

1.18 Biochemistry analysis of IR-FEP-RGD-S-S-S-Fc

After injection intravenously with PBS, IR-FEP, IR-FEP-RGD, and IR-FEP-RGD-S-S-S-Fc (50 μM, 100 μL), respectively, the healthy mice blood was

collected for biochemistry analysis. The main organs (heart, liver, spleen, lungs, and kidneys) of the mice were stained with H&E for pathological analysis.

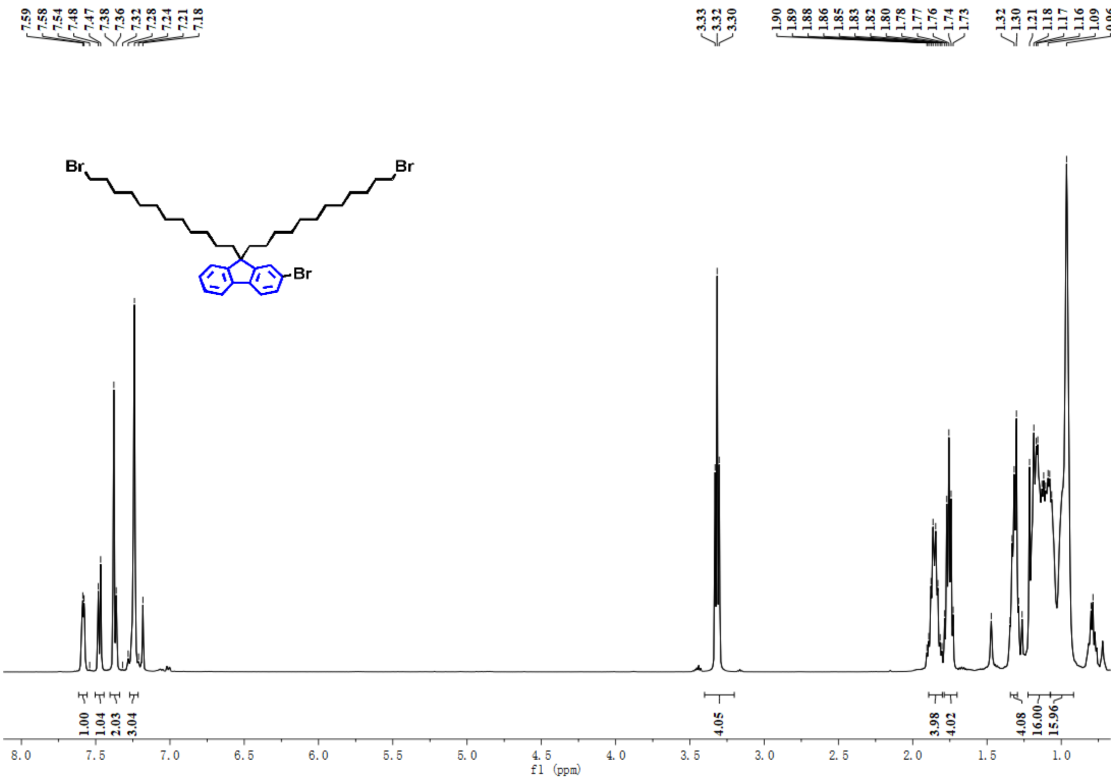

Figure S1. ^{1}H NMR of compound 2

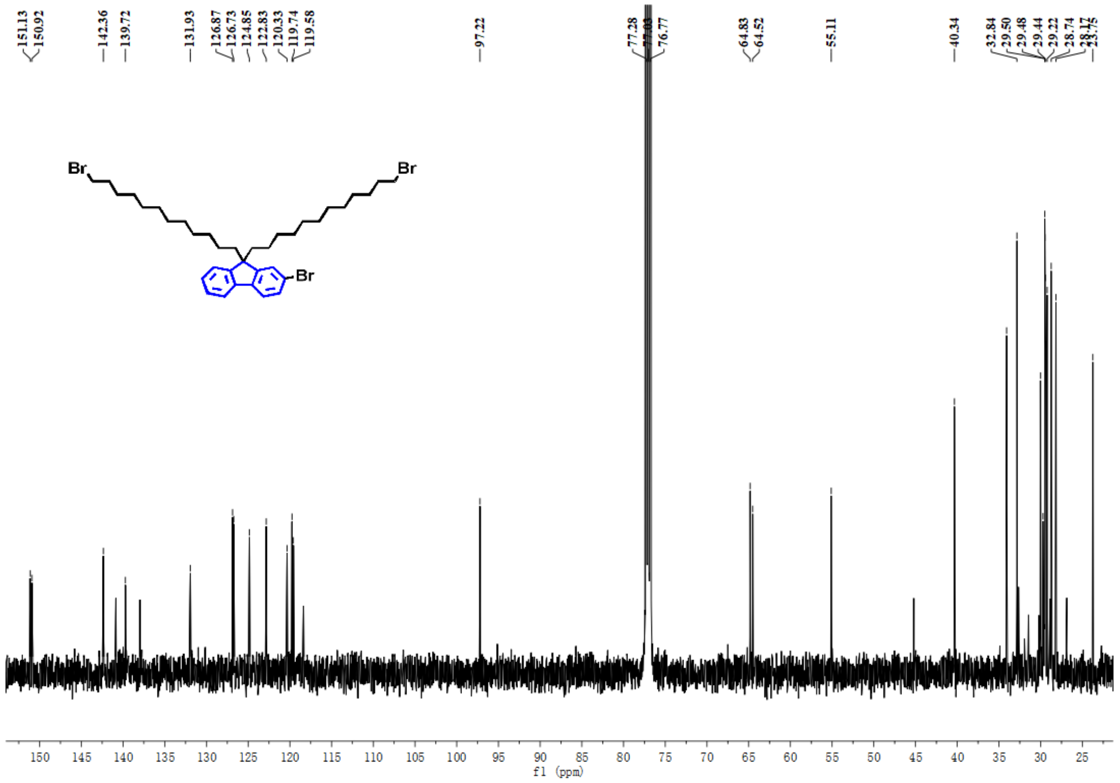

Figure S2. ^{13}C NMR of compound 2

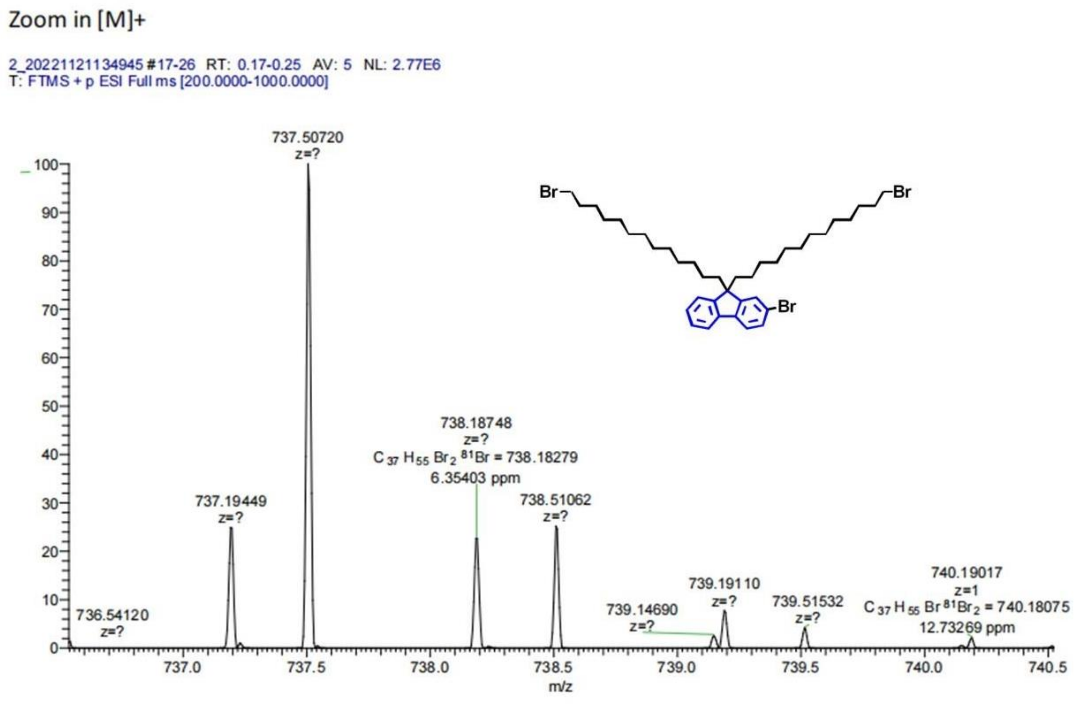

Figure S3. HMRS of compound 2

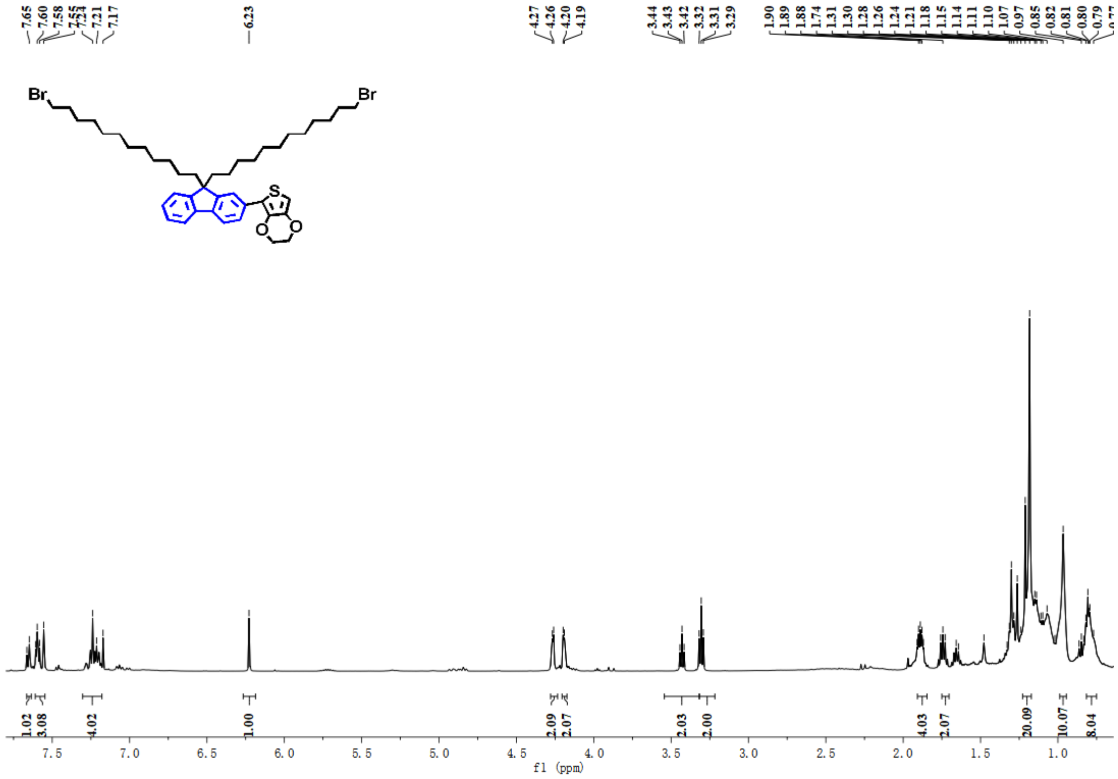

Figure S4. ^{1}H NMR of compound 5

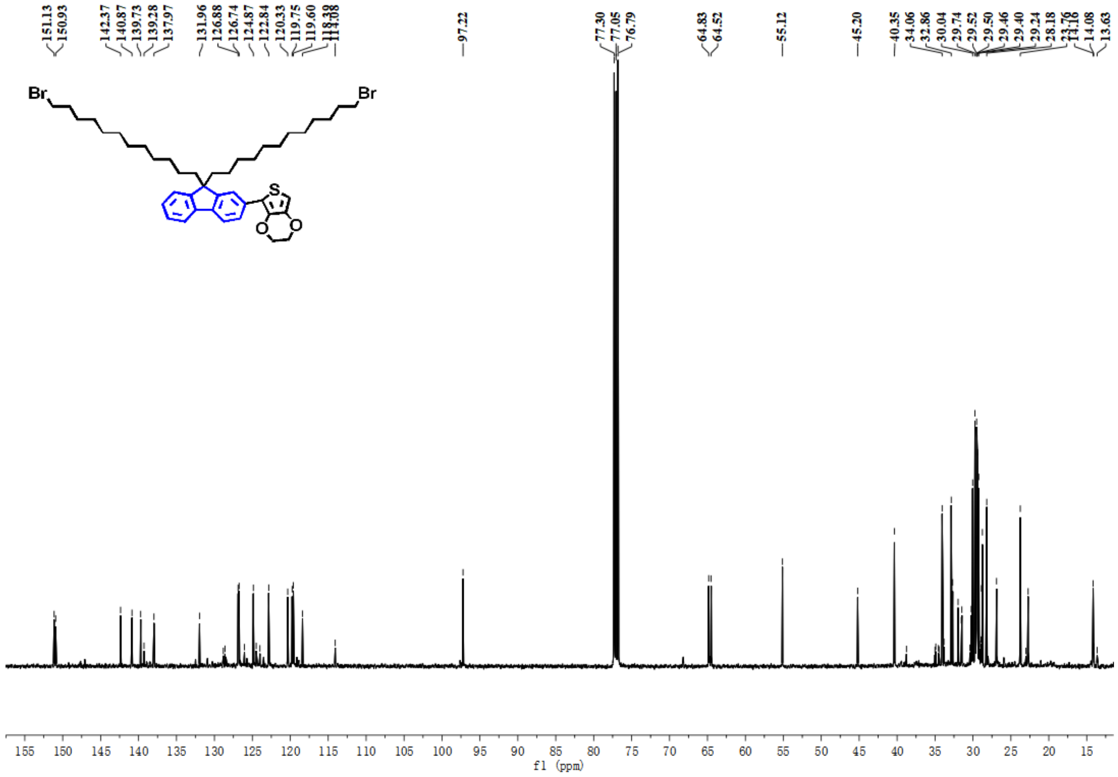

Figure S5. ^{13}C NMR of compound 5

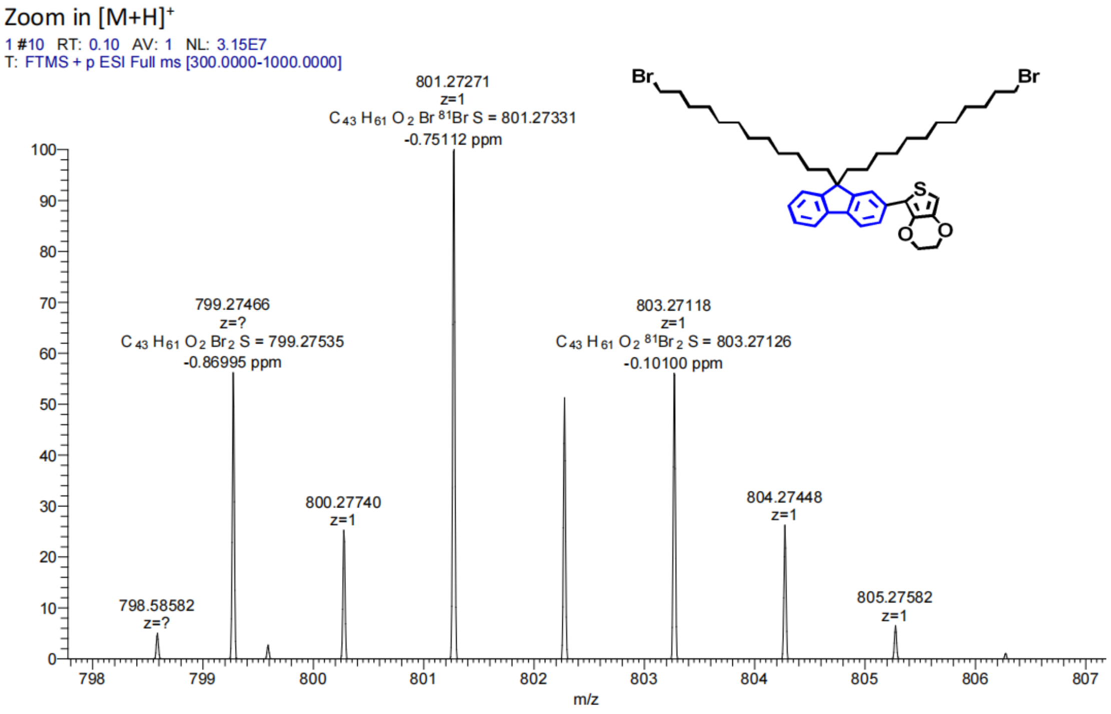

Figure S6. HMRS of compound 5

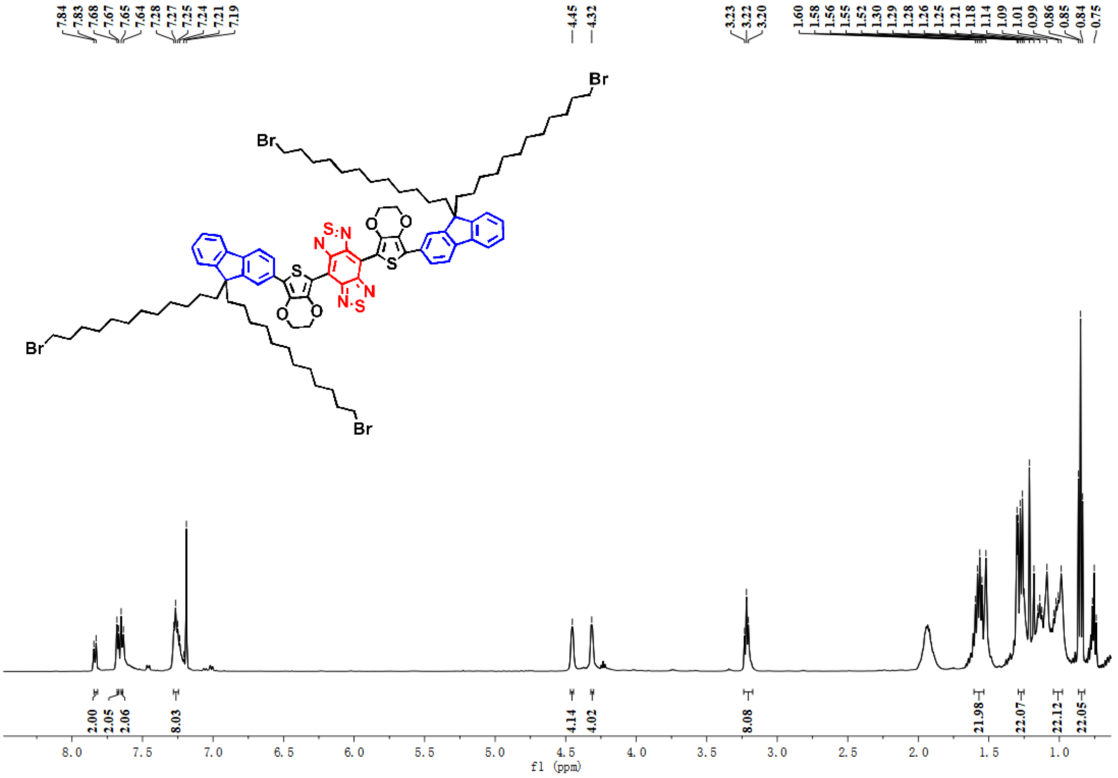

Figure S7. ^{1}H NMR of compound IR-FE (C_{12})

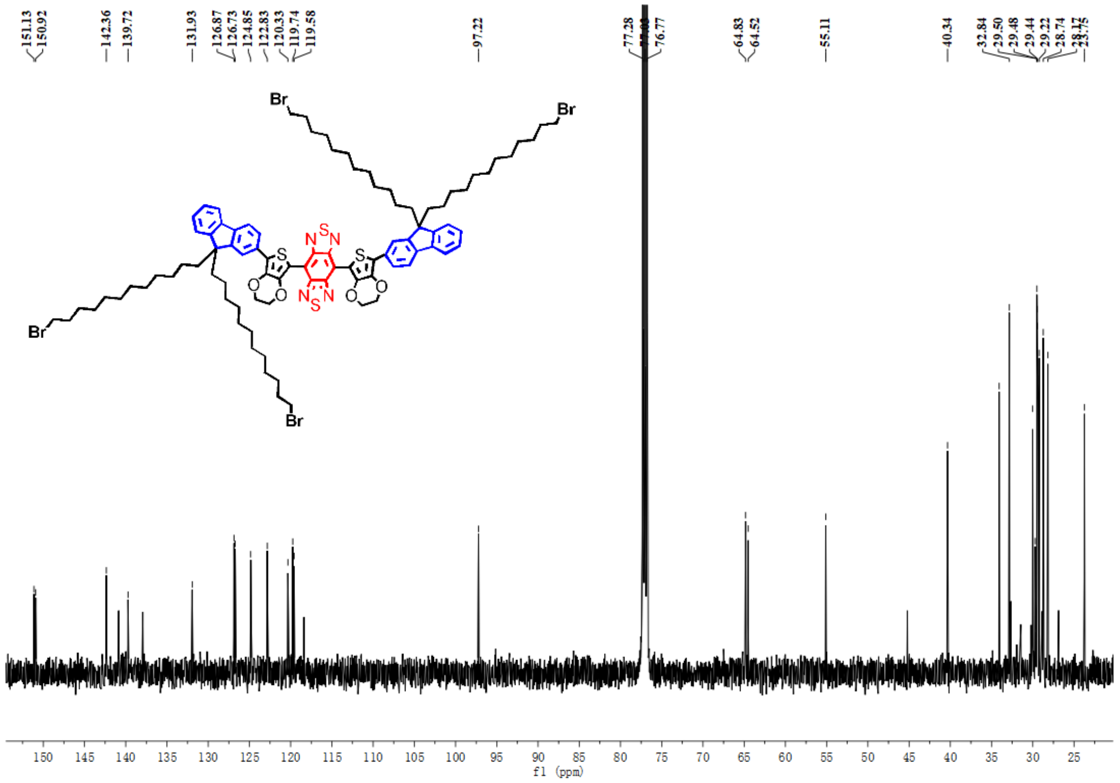

Figure S8. ^{13}C NMR of compound IR-FE (C_{12})

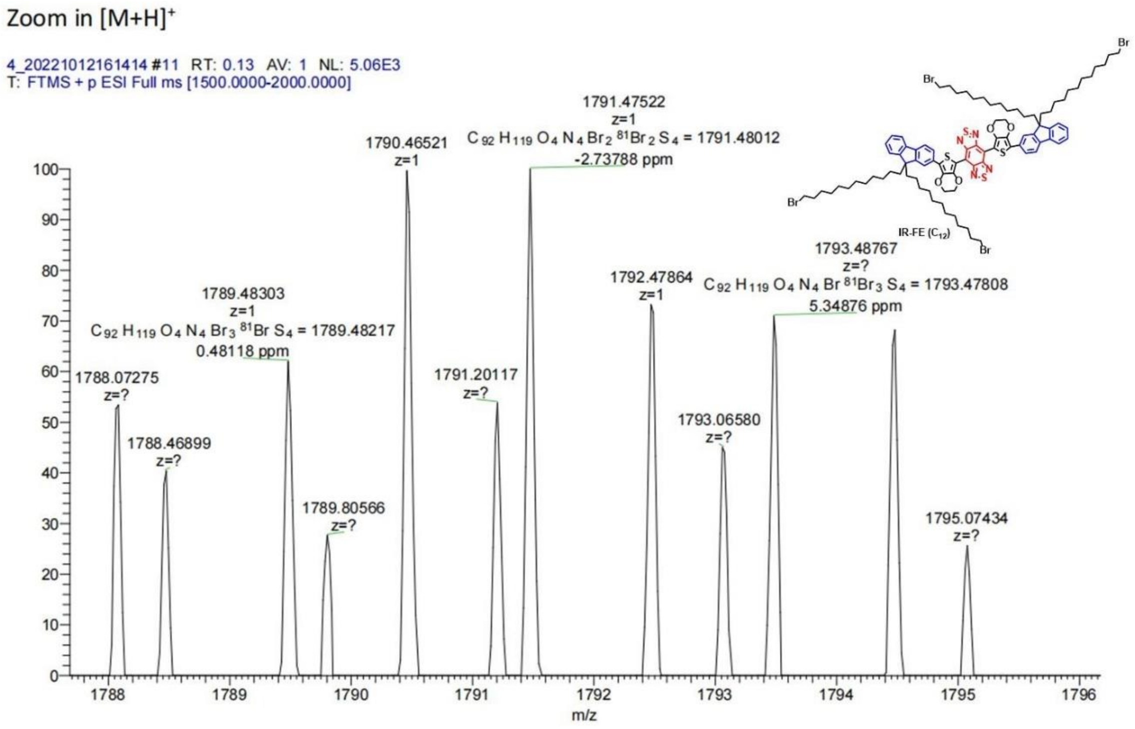

Figure S9. HRMS of compound IR-FE (C_{12}).

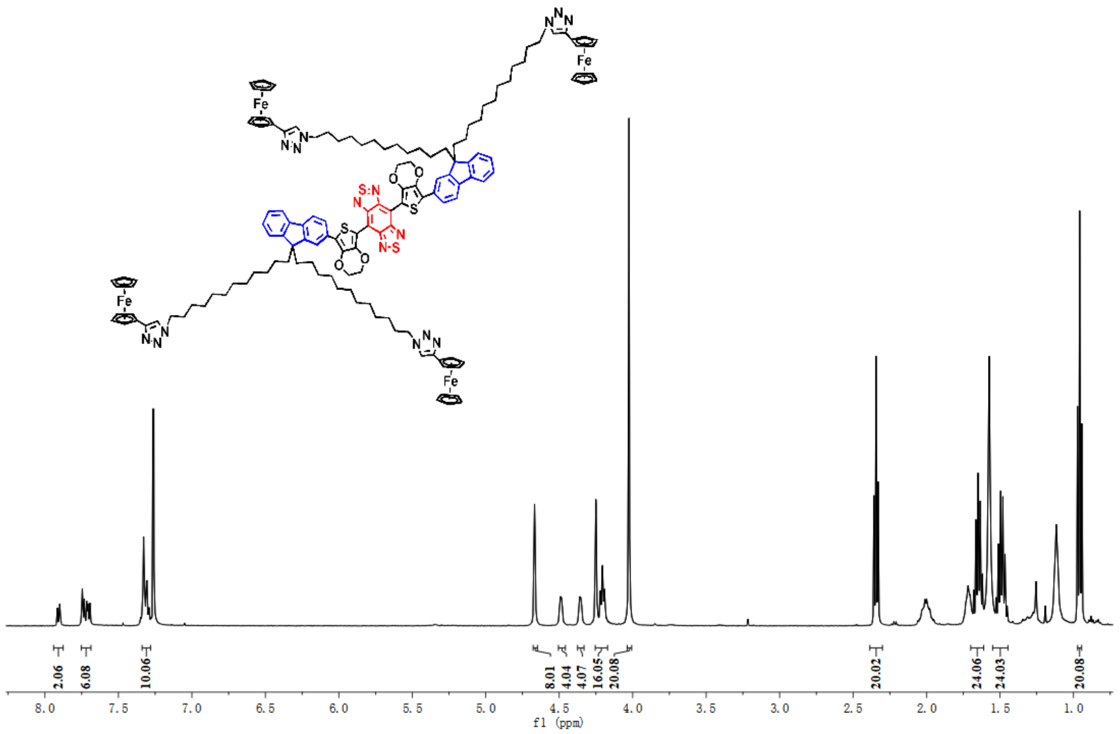

Figure S10. ^{1}H NMR of compound IR-FE-Fc (C_{12})

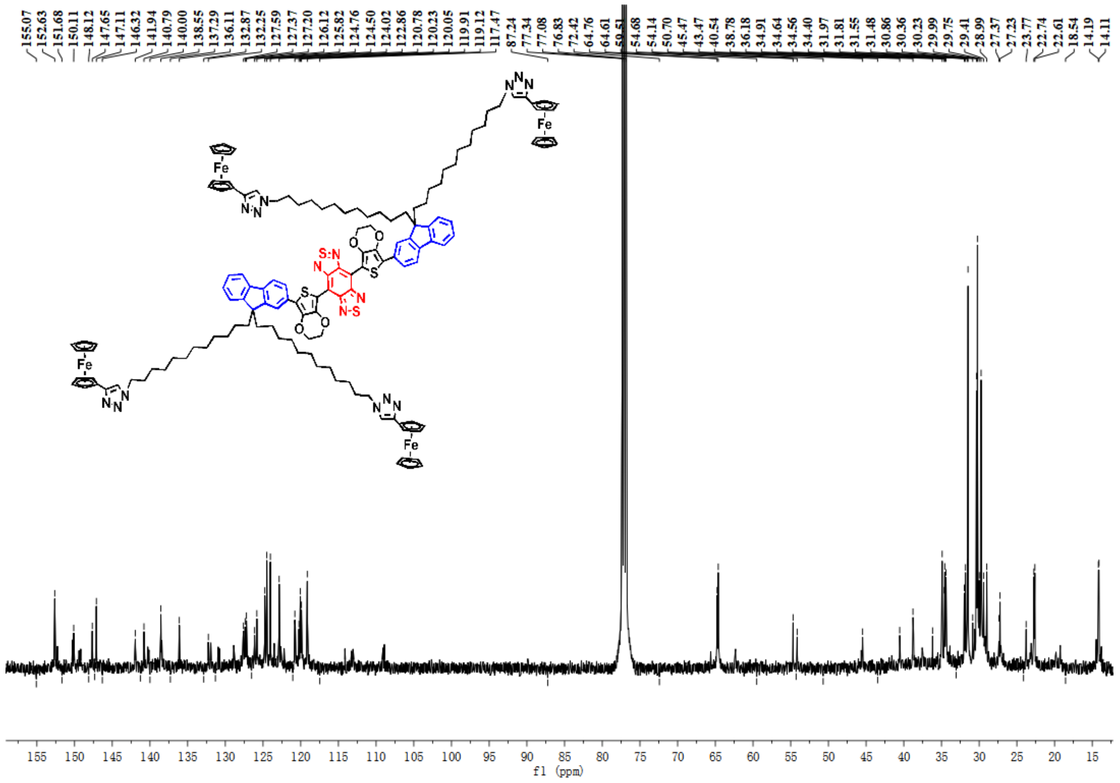

Figure S11. ^{13}C NMR of compound IR-FE (C_{12})

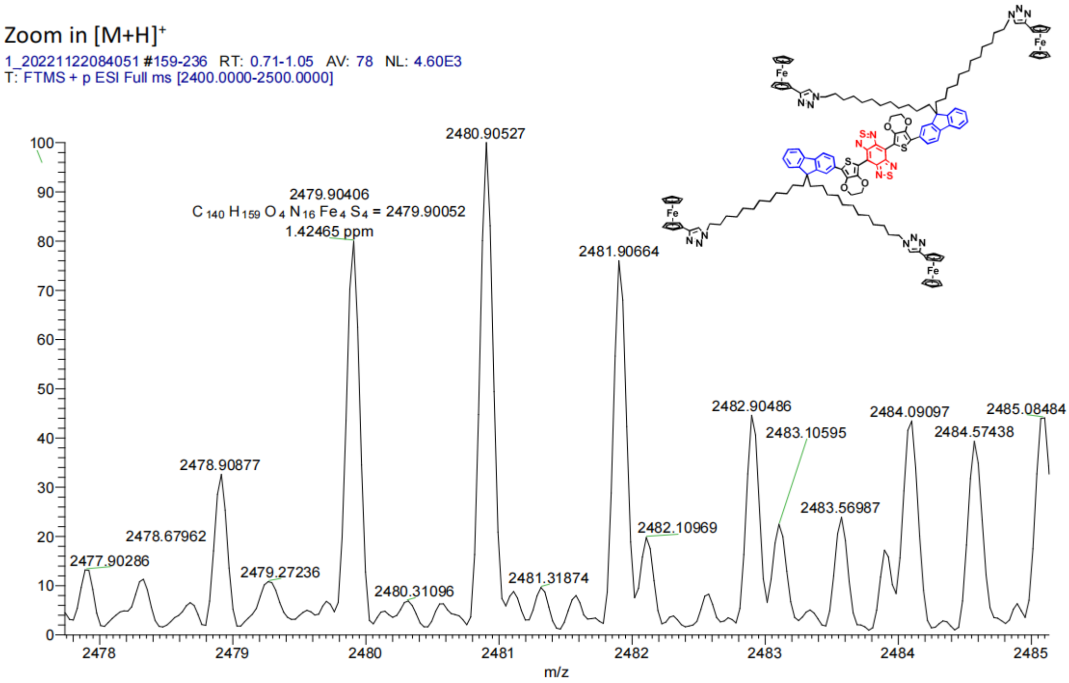

Figure S12. HRMS of compound IR-FE-Fc (C_{12})

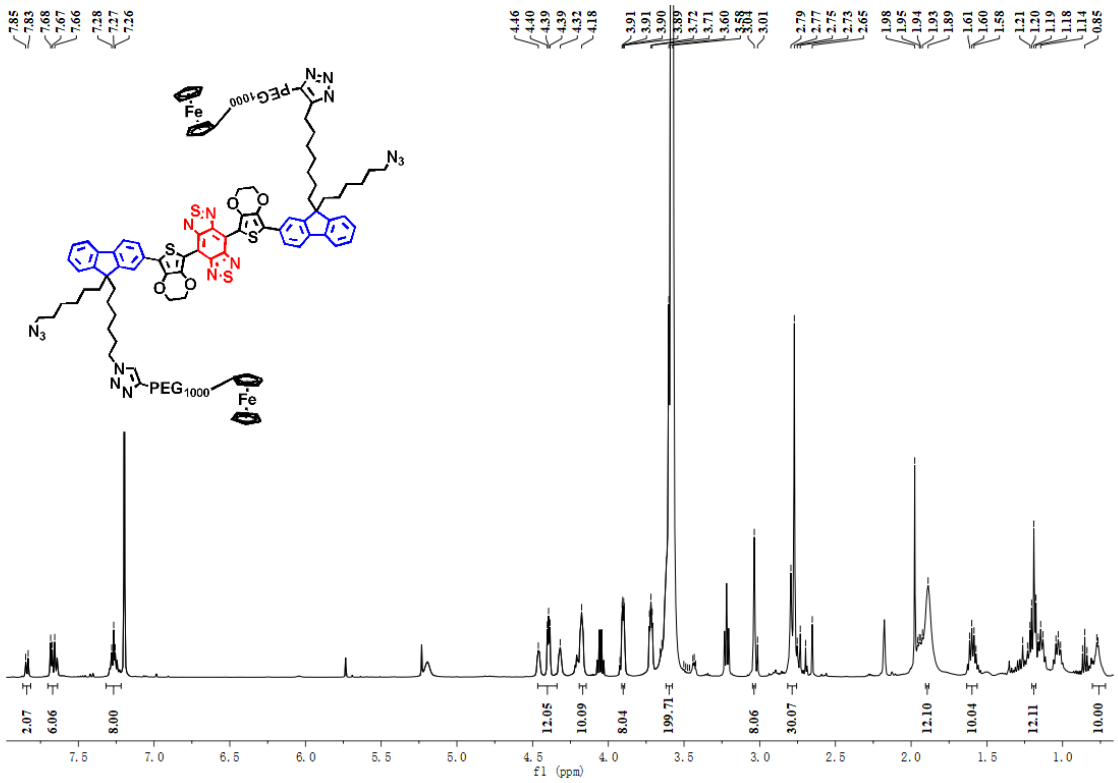

Figure S13. ^{1}H NMR of compound IR-FEP-Fc

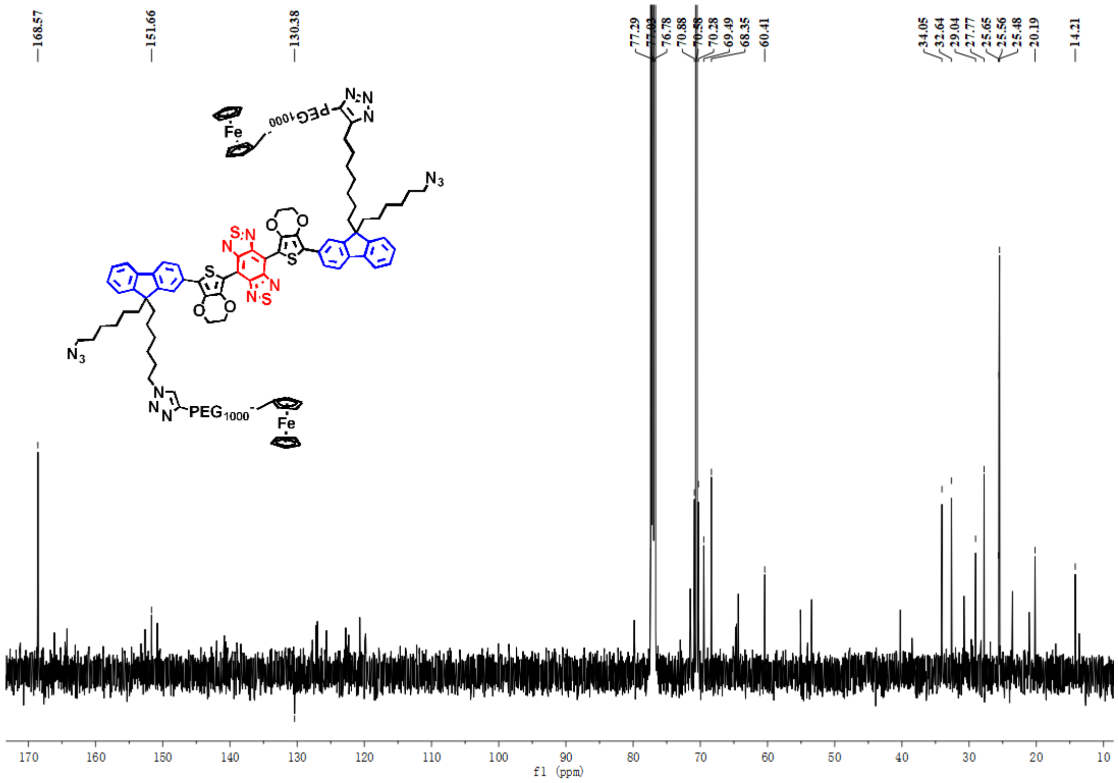

Figure S14. ^{13}C NMR of compound IR-FEP-Fc

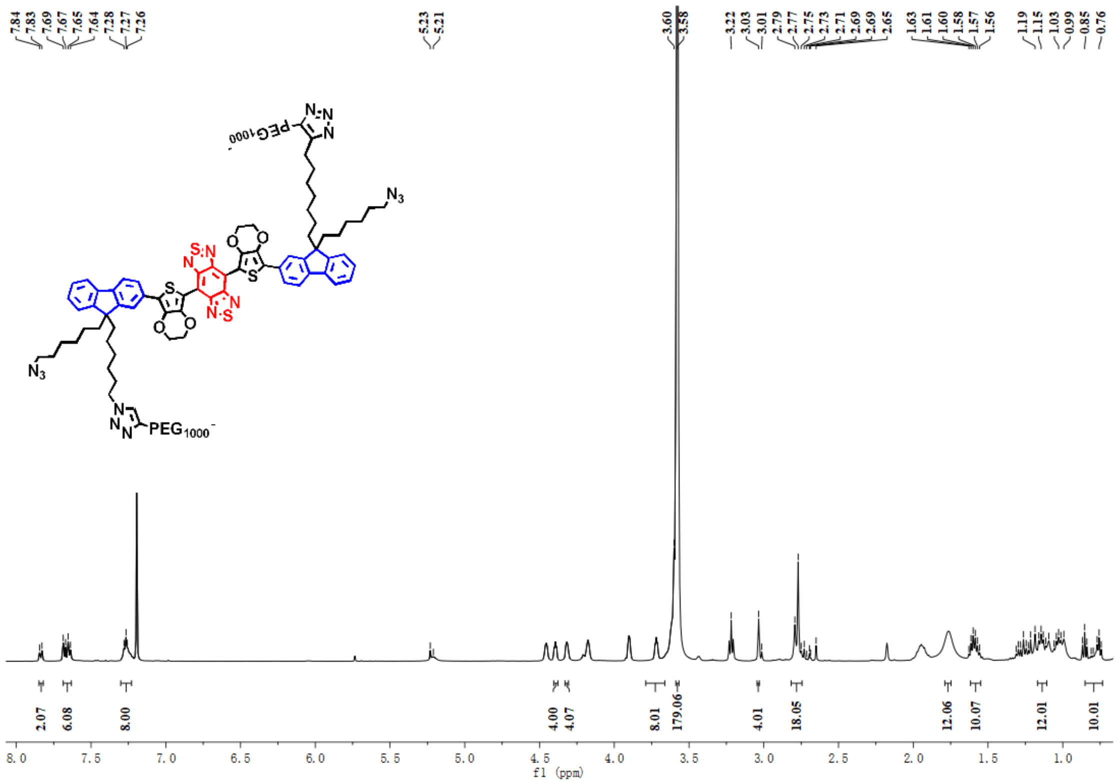

Figure S15. ^{1}H NMR of compound IR-FEP

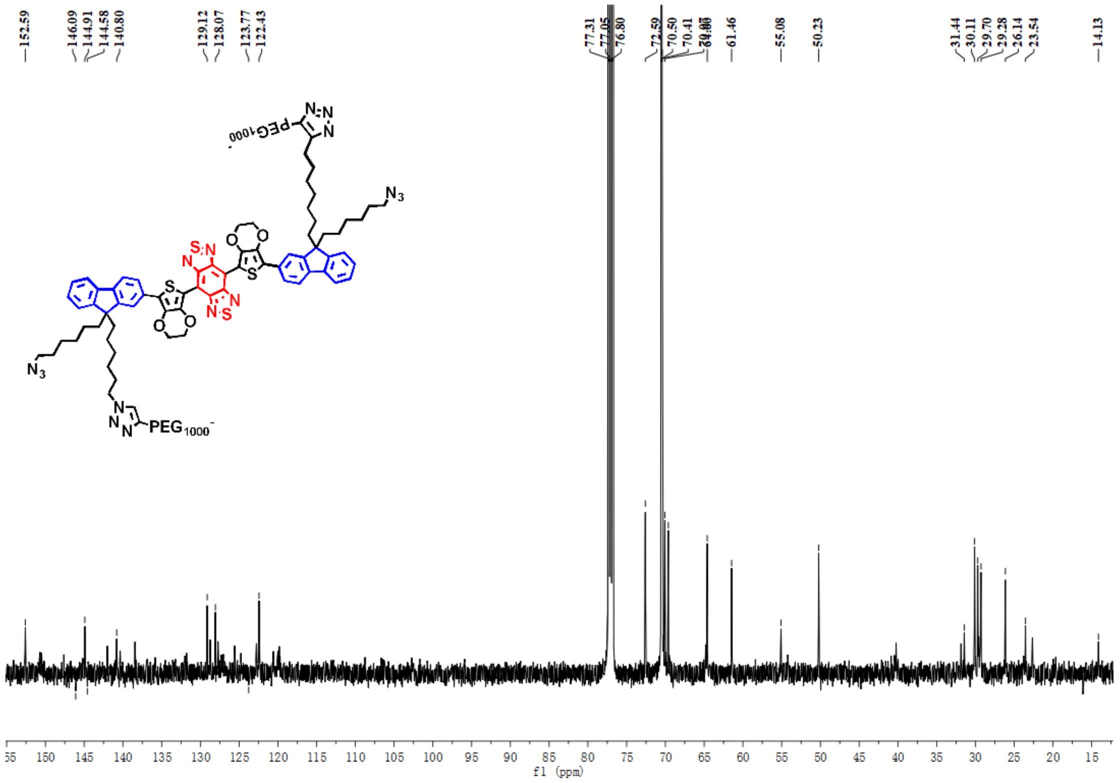

Figure S16. ^{13}C NMR of compound IR-FEP

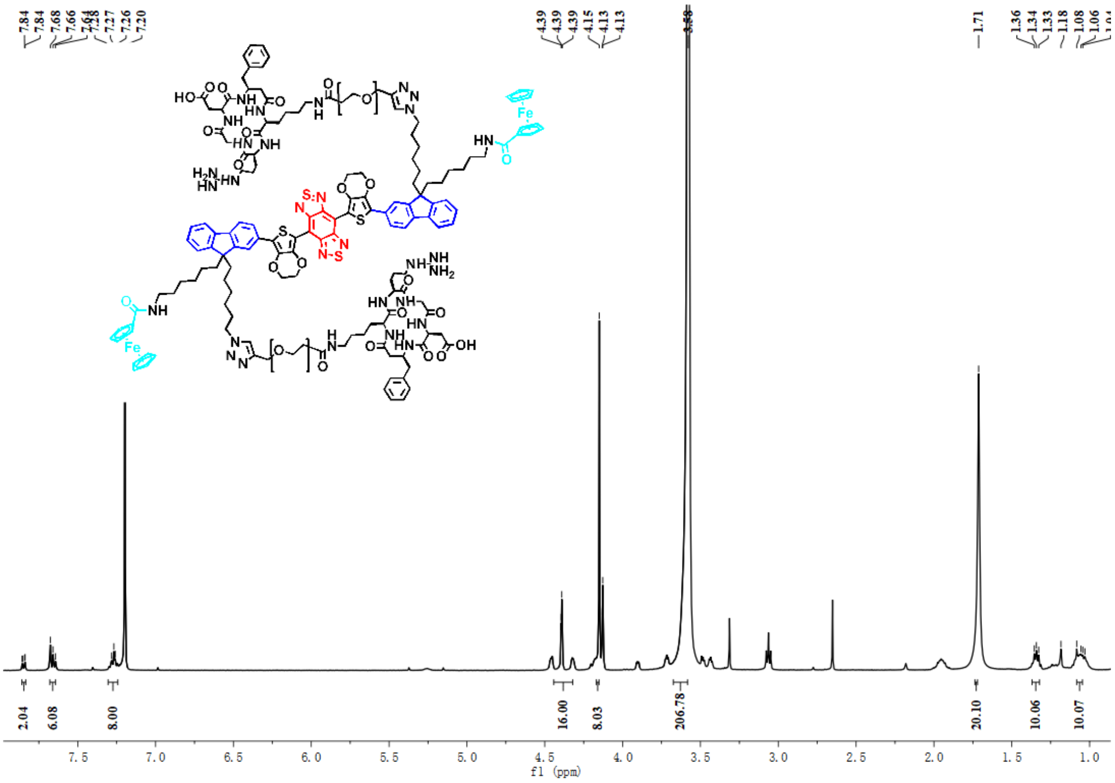

Figure S17. ^{1}H NMR of compound IR-FEP-RGD -Fc

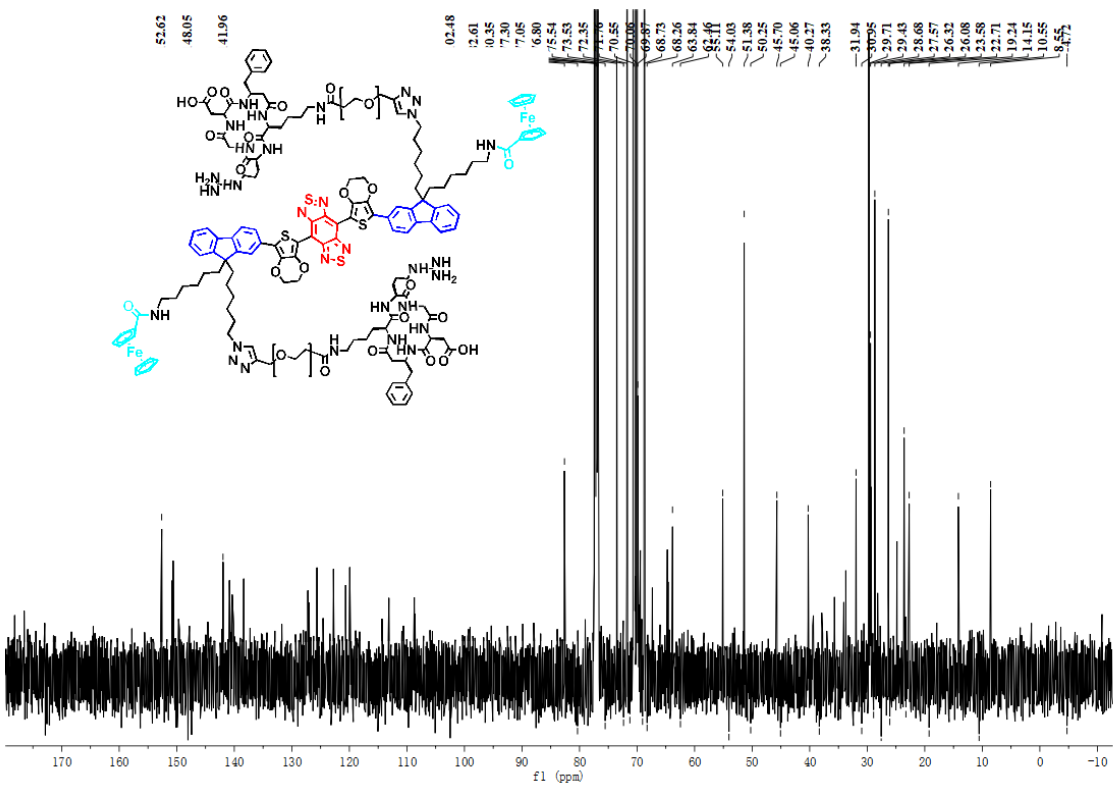

Figure S18. 1C NMR of compound IR-FEP-RGD-Fc

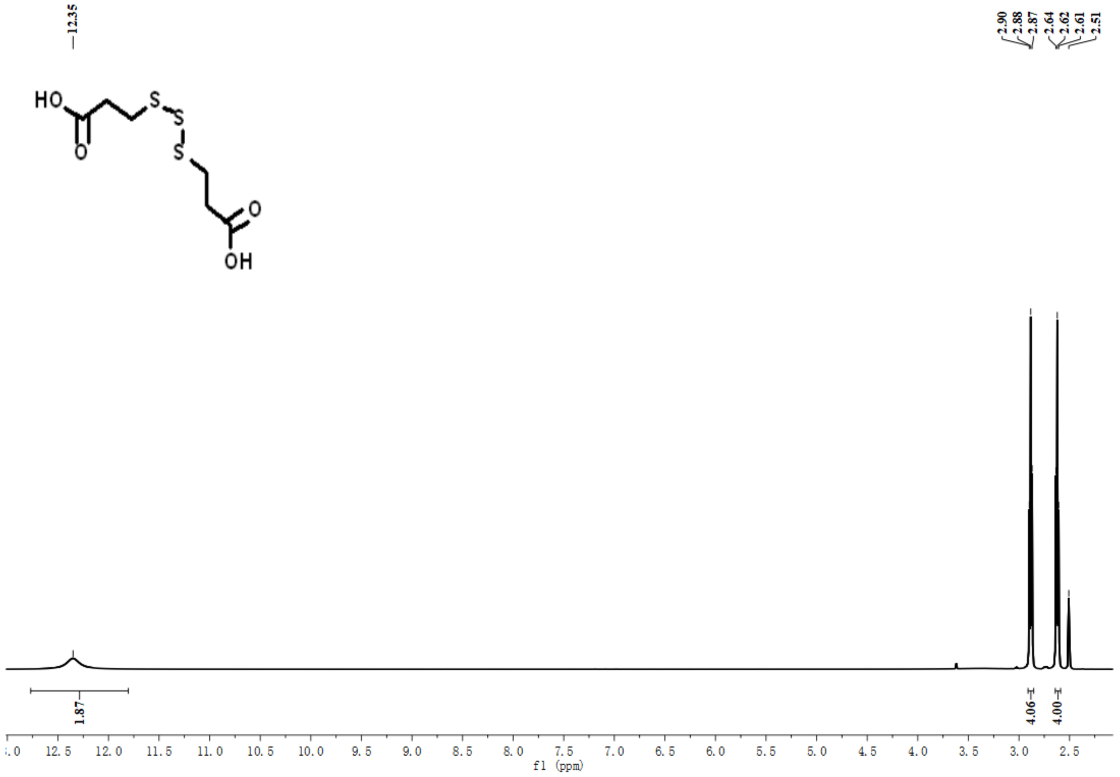

Figure S19. 1H NMR of compound 7

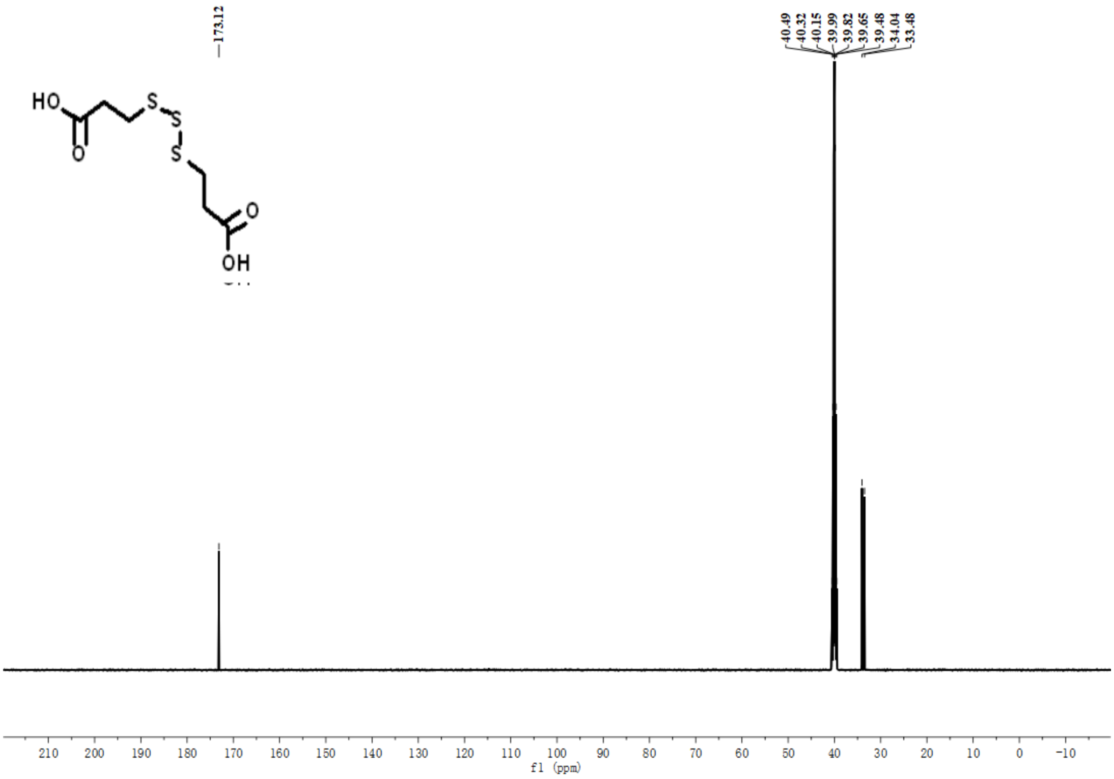

Figure S20. ^{13}C NMR of compound 7

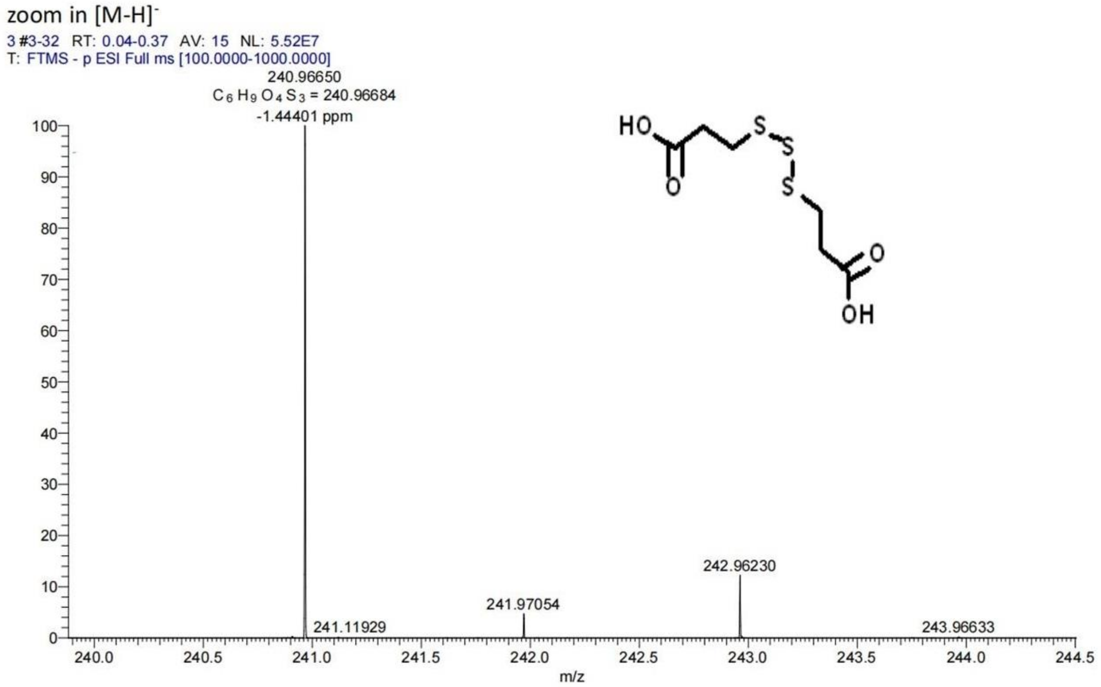

Figure S21. HRMS of compound 7

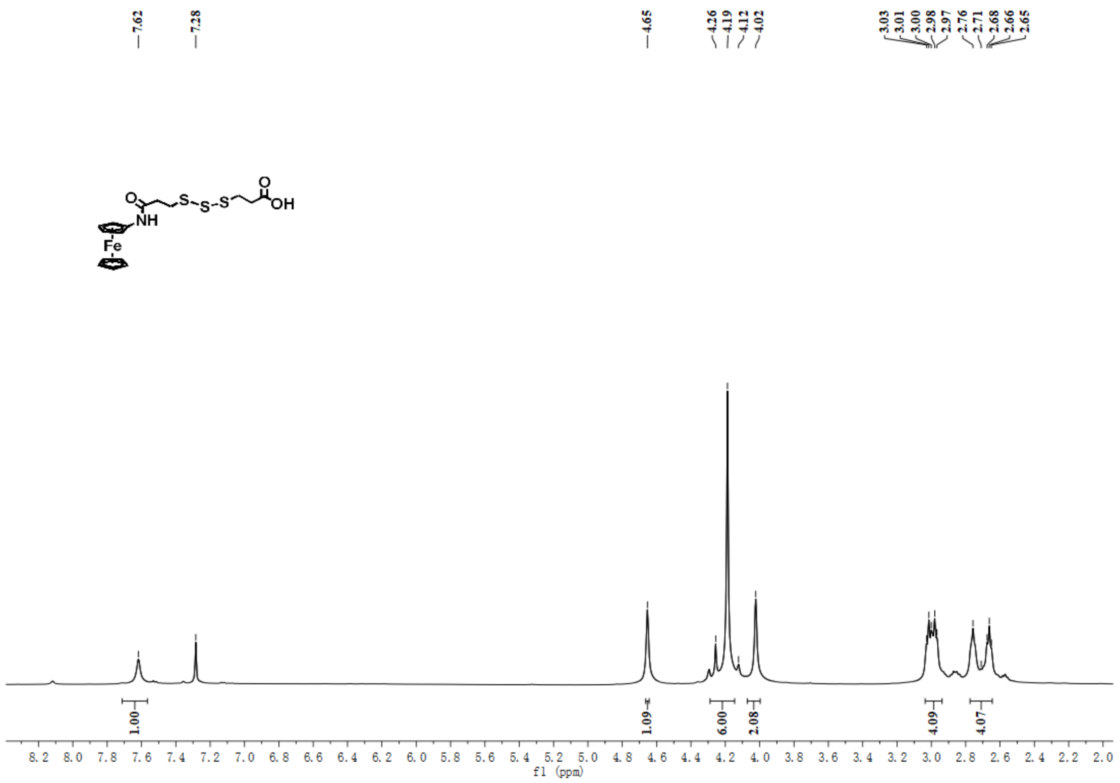

Figure S22. ^{1}H NMR of compound Fc-S-S-S-COOH

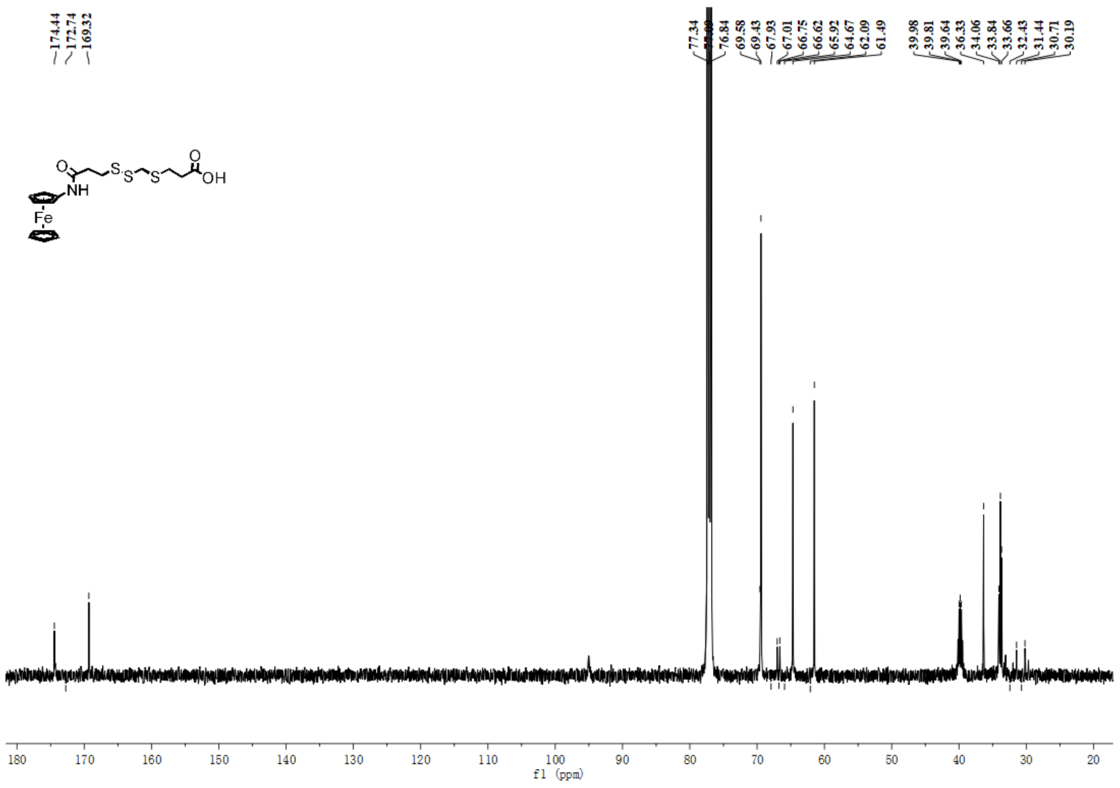

Figure S23. ^{13}C NMR of compound Fc-S-S-S-COOH

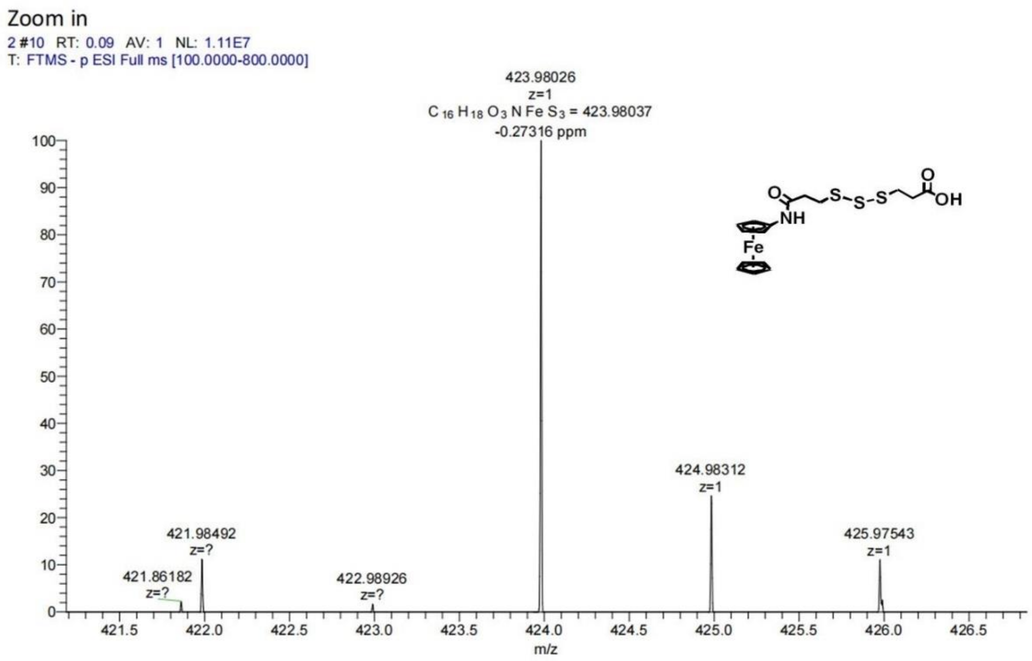

Figure S24. HRMS of compound Fc-S-S-S-COOH

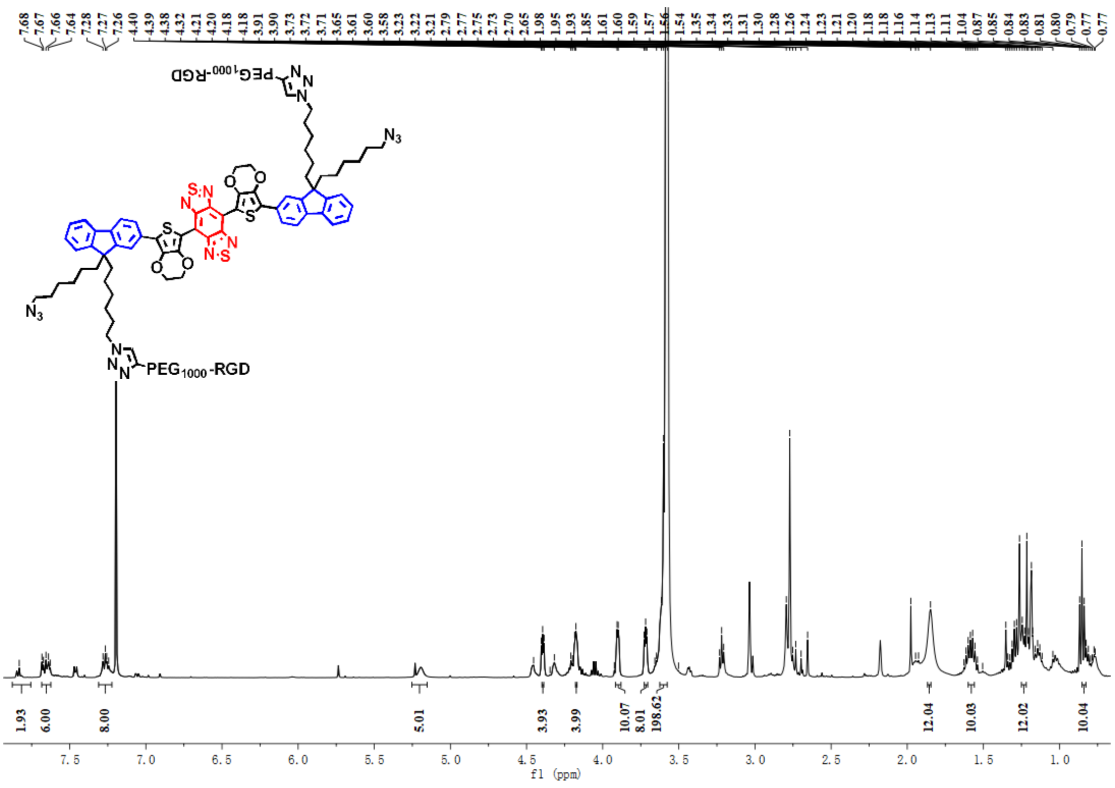

Figure S25. ^{1}H NMR of compound IR-FEP-RGD

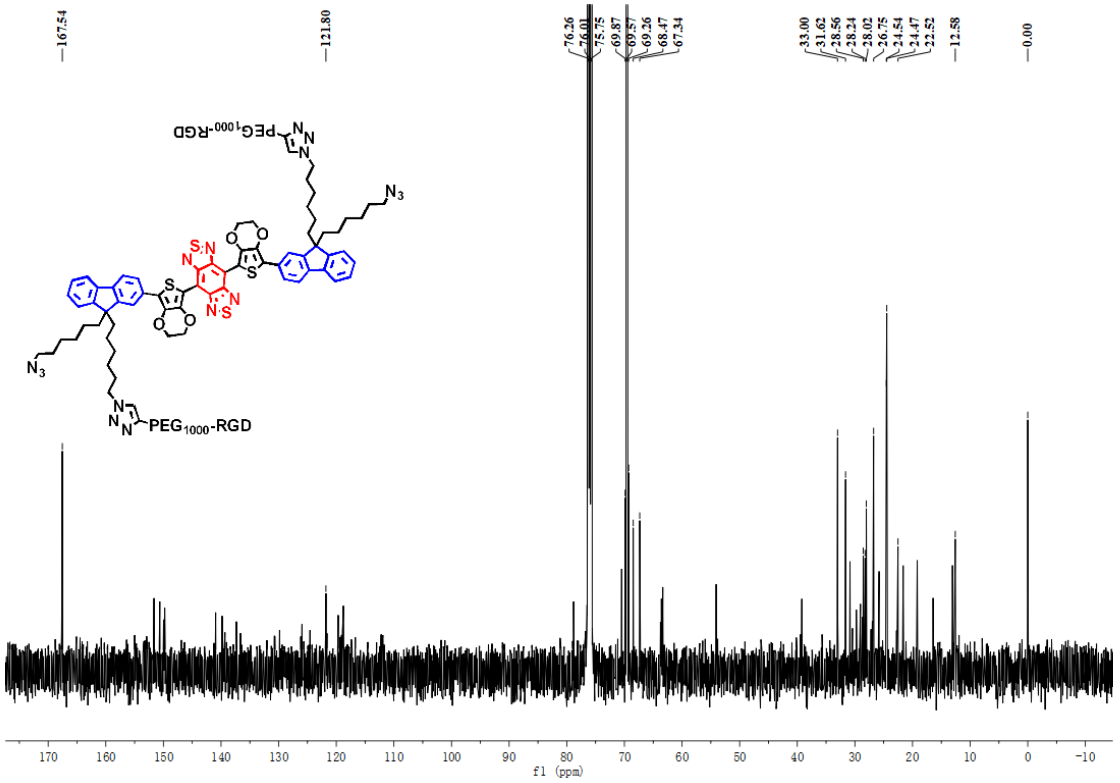

Figure S26. ^{13}C NMR of compound IR-FEP-RGD

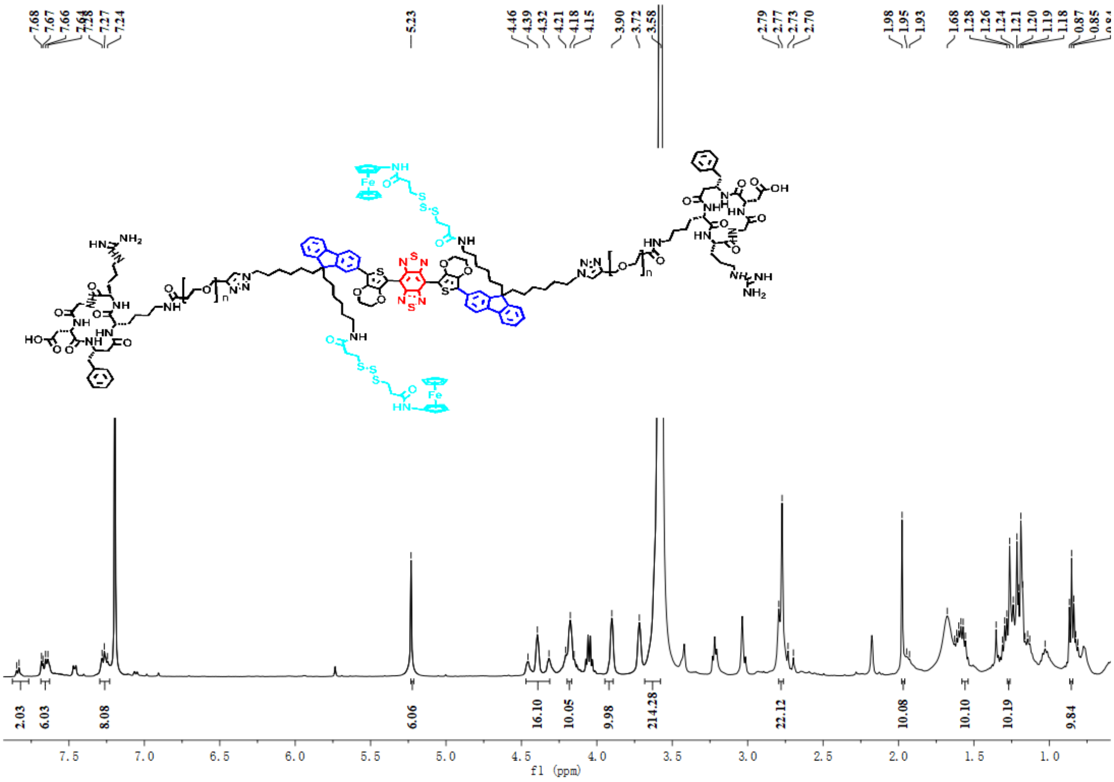

Figure S27. ^{1}H NMR of compound IR-FEP-RGD-S-S-S-Fc

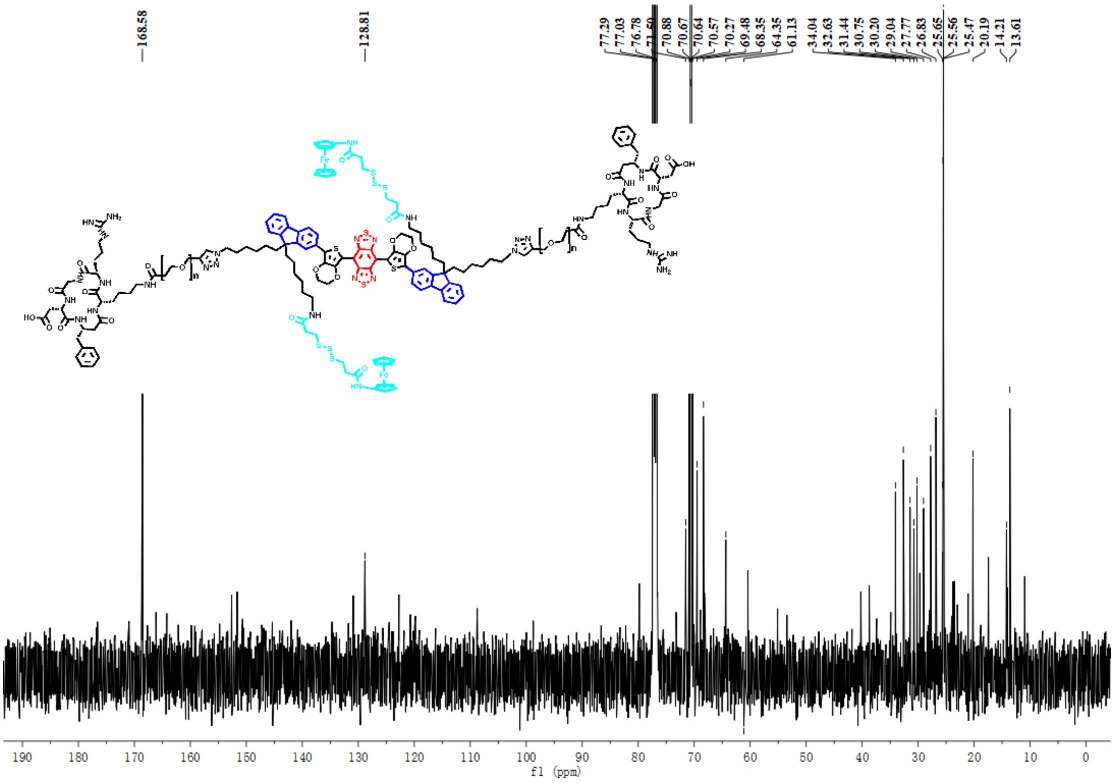

Figure S28. ^{13}C NMR of compound IR-FEP-RGD-S-S-S-Fc

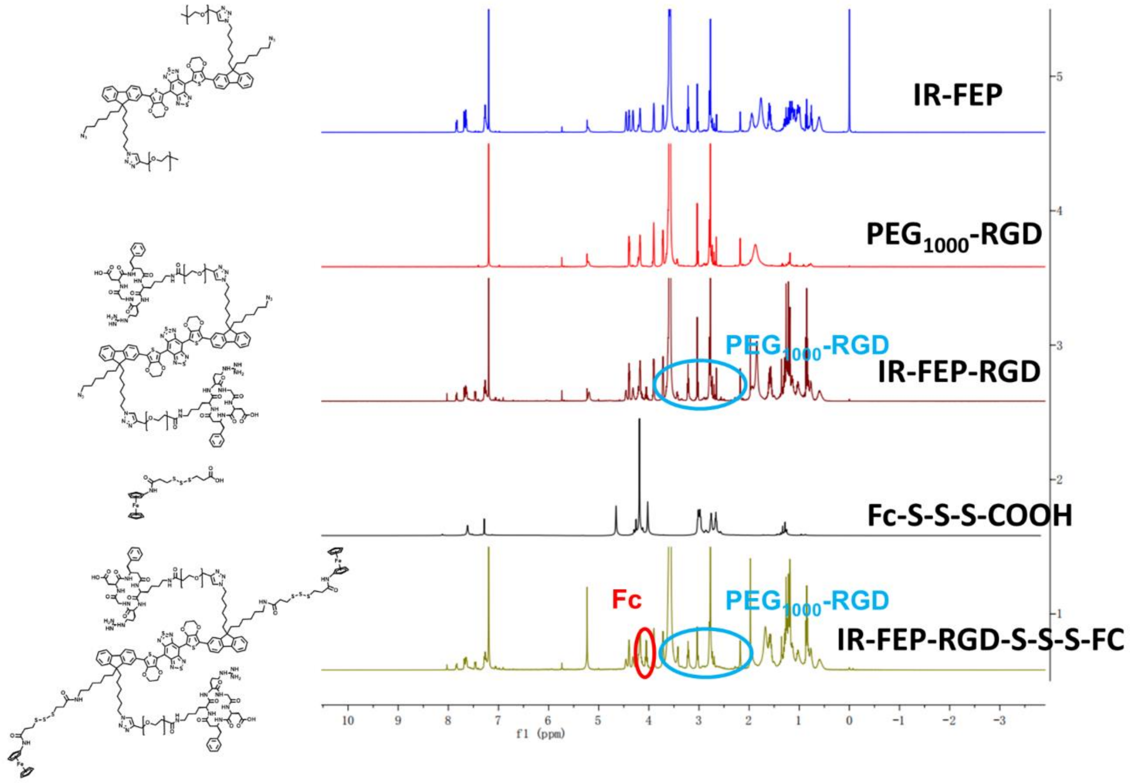

Figure S29. ^{1}H NMR spectra of IR-FEP, IR-FEP-Fc, IR-FEP-RGD and IR-FEP-RGD-S-S-S-Fc in $CDCl_3$

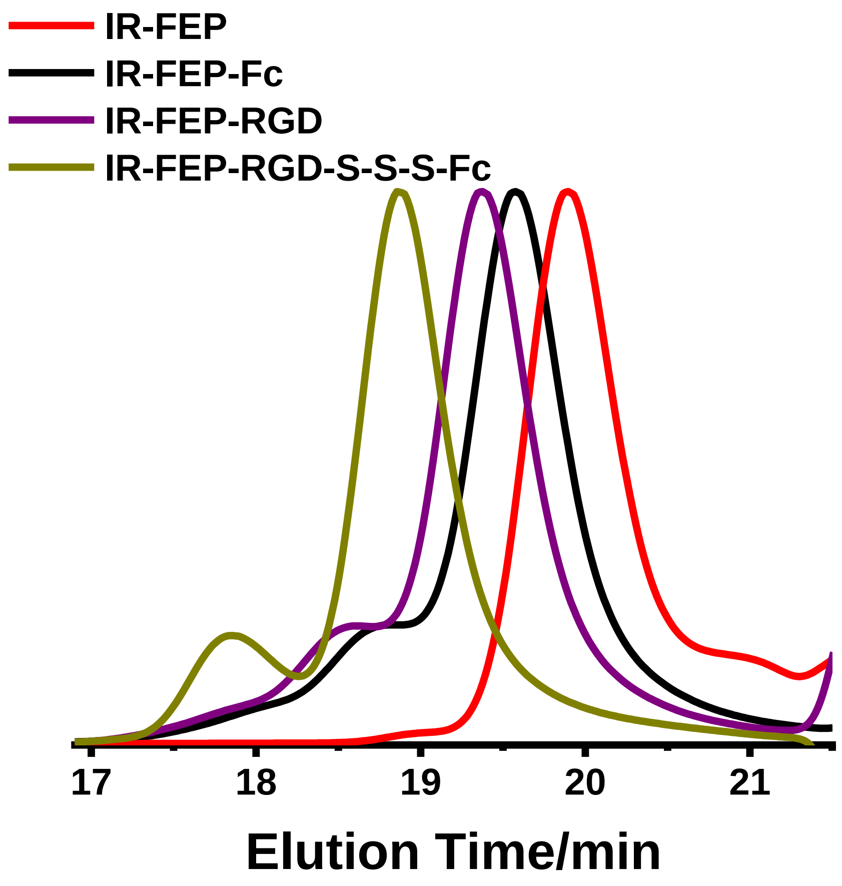

Figure S30. SEC elution traces of IR-FEP, IR-FEP-Fc, IR-FEP-RGD, and IR-FEP-RGD-S-S-S-Fc.

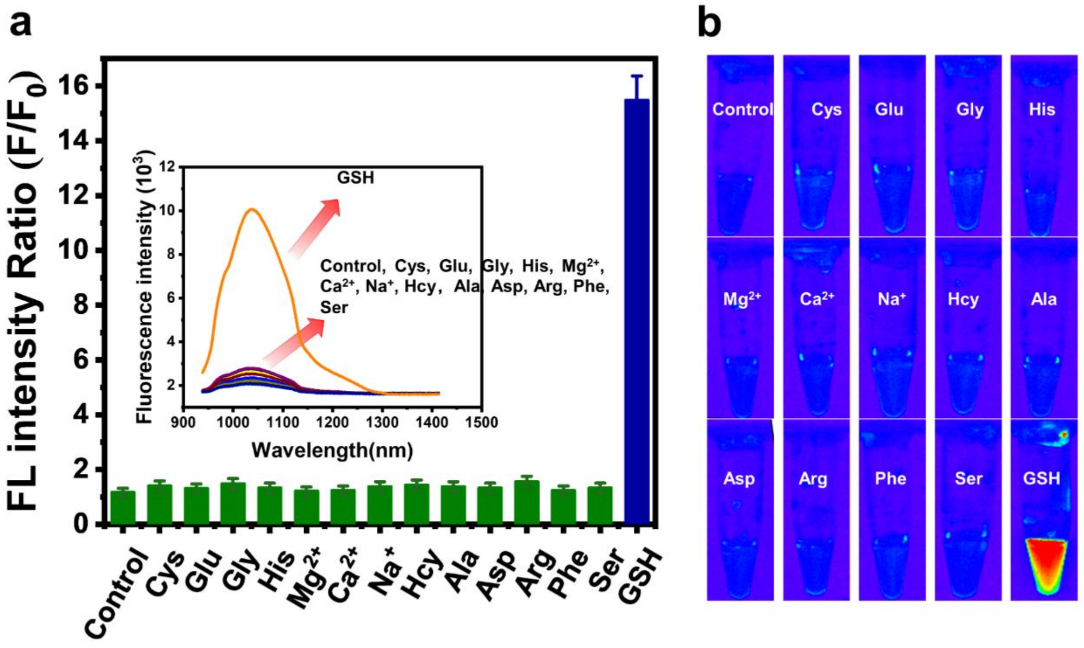

Figure S31. (a) Relative fluorescence intensity ratios of IR-FEP-RGD-S-S-S-Fc (50 μM) with the addition of 10 mM of various amino acids (GSH, Cys, Glu, Gly, His, Phe, Ser, Val, Asp, Ala, Arg, Hcy) and 10 mM metal ions (Na^{+}, $Mg^{2+,}$ Ca^{2+}). Insert: Fluorescence spectra of IR-FEP-RGD-S-S-S-Fc (50 μM) to various amino acids and metal ions (10 mM). F and F0 represent the fluorescence intensity of IR-FEP-RGD-S-S-S-Fc with or without the addition of GSH, respectively; (b) Corresponding fluorescence images of IR-FEP-RGD-S-S-S-Fc in the presence of different amino acids and metal ions detected by NIR-II fluorescence imaging system. Error bars: mean ± SD (n = 3).

a

b

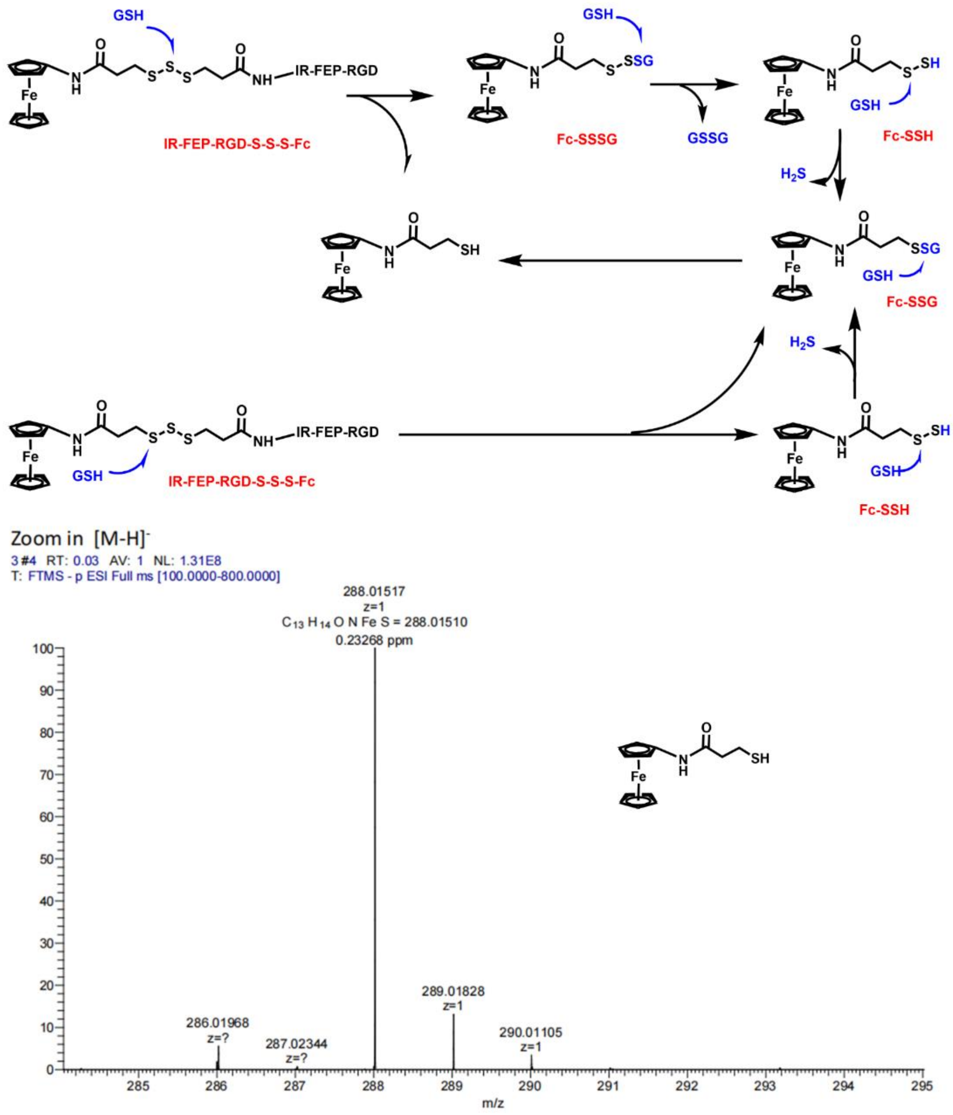

Figure S32. GSH-triggered breakage of trisulfide bonds (a) and (b)HRMS spectra of IR-FEP-RGD-S-S-S-Fc after incubation with 10 mM GSH-containing release media.

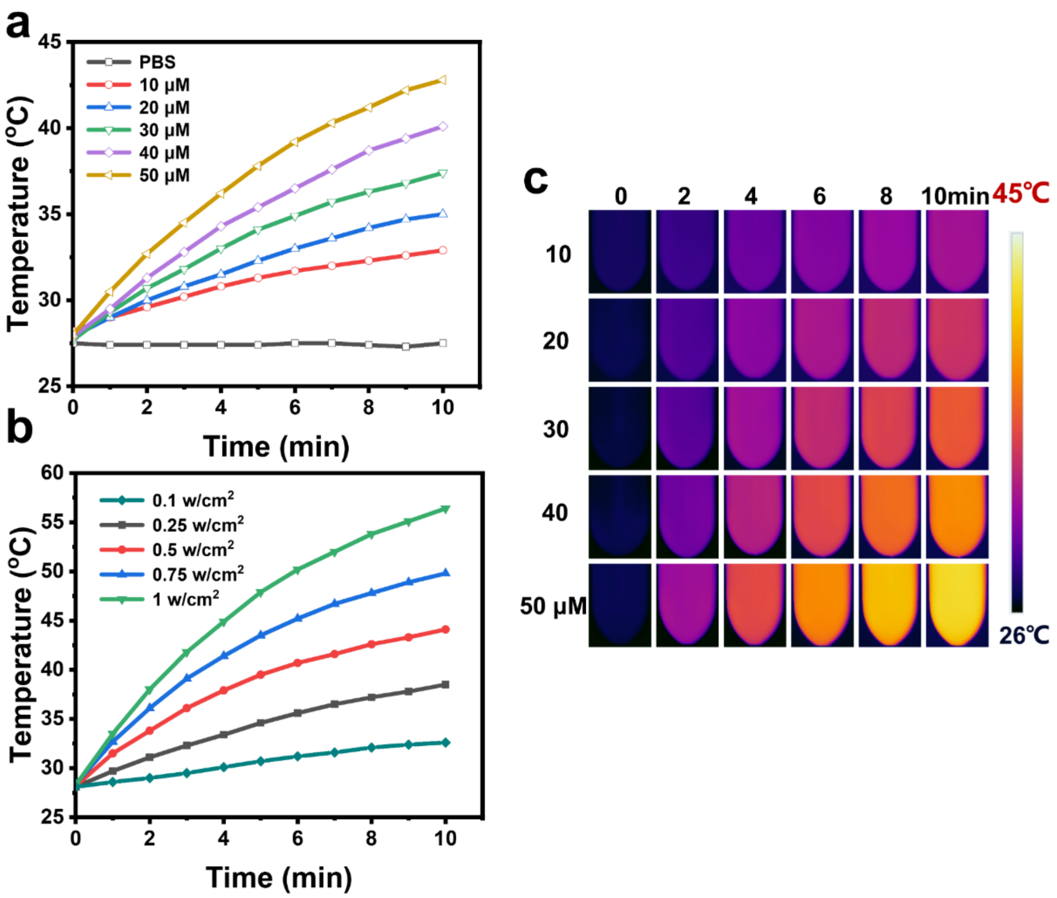

Figure S33. (a) The temperature changes of different concentrations of IR-FEP-RGD-S-S-S-Fc (50 μM) under 808 nm laser irradiation (0.33 W/cm^2). (b) Heating curve of IR-FEP-RGD-S-S-S-Fc (50 μM) under 808 nm laser irradiation of different power densities. c) Infrared thermal images of IR-FEP-RGD-S-S-S-Fc of different concentrations.

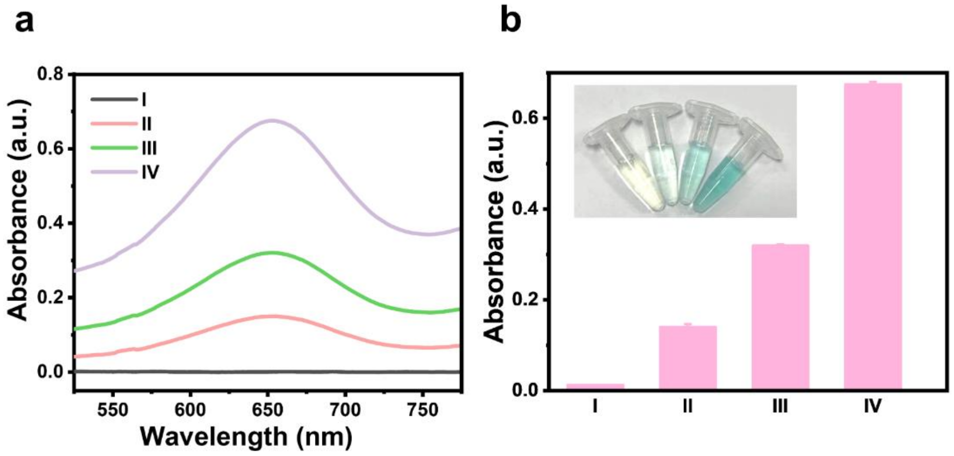

Figure S34. (a) The absorption spectra of TMB aqueous solution with different treatments (I TMB + IR-FEP-RGD-S-S-S-Fc − L, II TMB + H_2O_2 + IR-FEP-RGD-S-S-S-Fc − L, III: TMB + H_2O_2 + IR-FEP-RGD-S-S-S-Fc + GSH, IV: TMB + H_2O_2 + IR-FEP-RGD-S-S-S-Fc +GSH + L, + L and − L are present as 808 nm laser/non-laser irradiation). (o) A652 TMB oxidation intensity of Figure S31a, Inset: images of relevant color variations). Error bars: mean ± SD (n = 4).

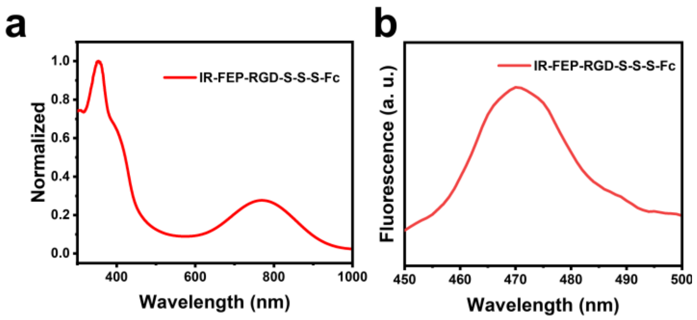

Figure S35. (a) Normalized NIR absorption of IR-FEP-RGD-S-S-S-Fc in aqueous solution. (b) The fluorescence emission spectra of IR-FEP-RGD-S-S-S-Fc excited at 405 nm.

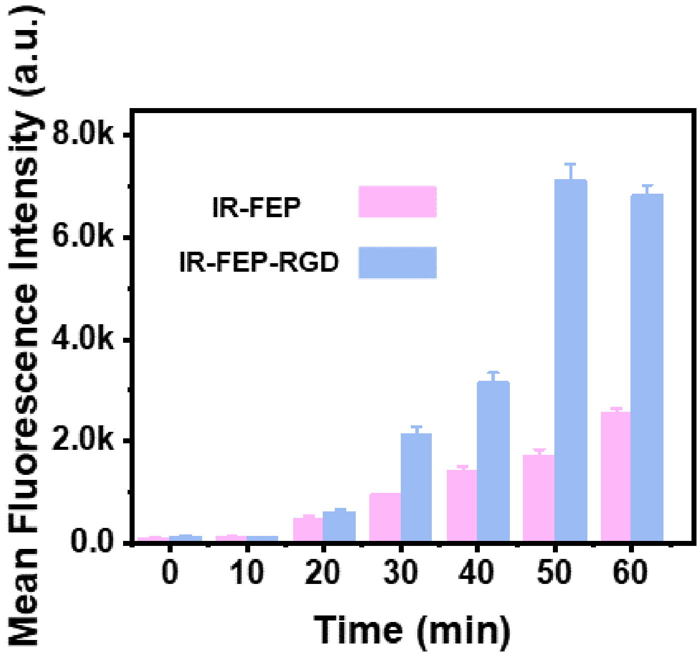

Figure S36. The MFI data of Figure 3c (Flow cytometric analysis of 4T1 cells incubated with IR-FEP, IR-FEP-RGD). Error bars: mean ± SD (n = 4).

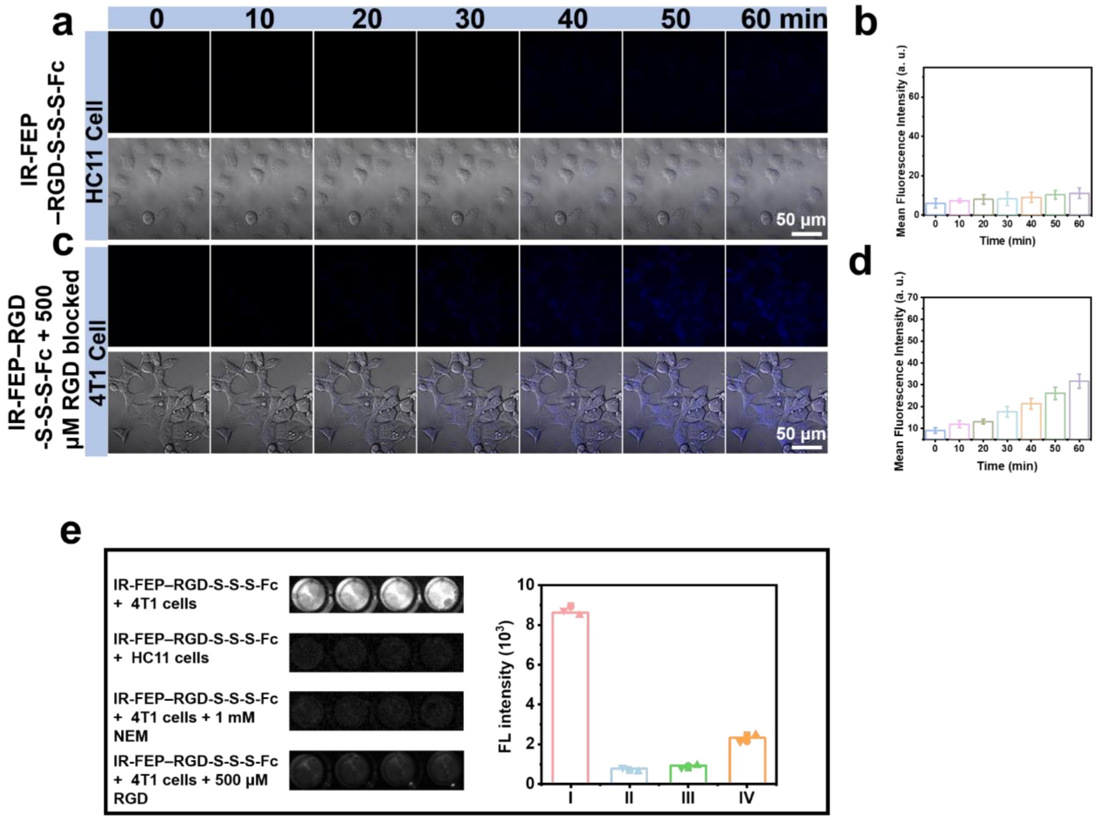

Figure S37. (a) CLSM images of HC11 cells incubated with IR-FEP-RGD-S-S-S-Fc 50 μM for 0-1 h. (b) Mean fluorescence intensity (MFI) of cells, which was quantified from (Figure S37a). Error bars: mean ± SD (n = 3). (c) CLSM images of 4T1 cells incubated with IR-FEP-RGD-S-S-S-Fc 50 μM with 500 μM RGD used for blocking integrin αvβ3 0-1 h. (d) Mean fluorescence intensity (MFI) of cells, which was quantified from (Figure S37c). Error bars: mean ± SD (n = 3). (e)The NIR-II fluorescence images and Semi-quantification of the fluorescence intensity of IR-FEP-RGD-S-S-S-Fc (50 μM) incubated with I: 4T1 cells, II: HC11 cells. III: 4T1 cells (+ 1 mM NEM)， and IV: 4T1 cells with 500 μM RGD used for blocking integrin αvβ3 after 1 h.

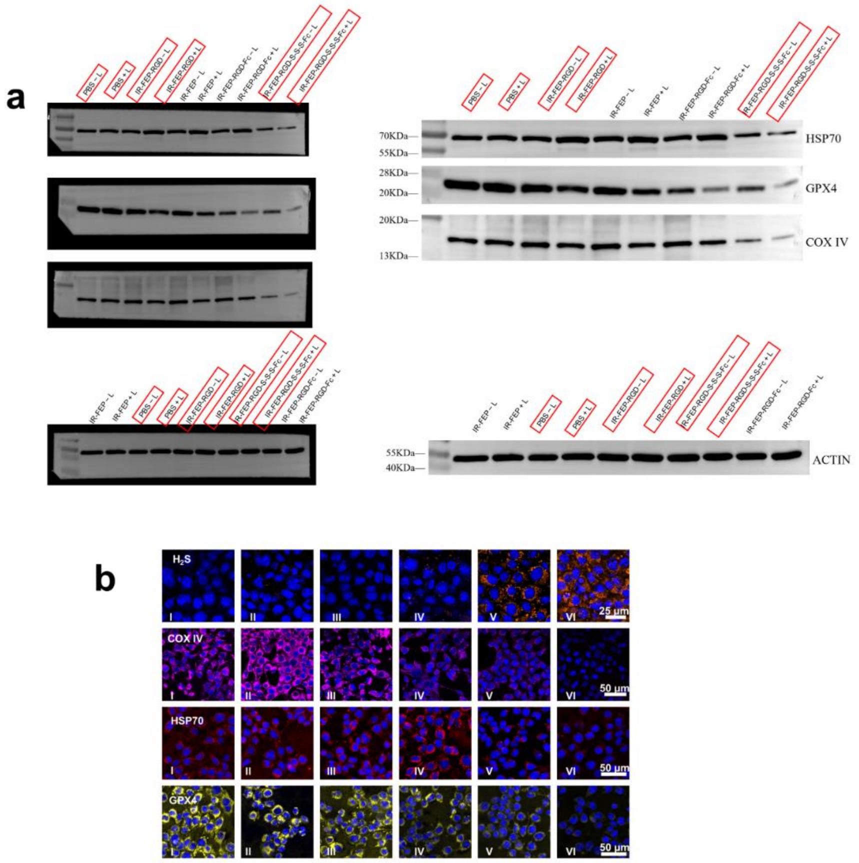

Figure S38. (a) The original Western blot images. (b)CLSM images of Intracellular H_2S detection using WSP-1 (marked as orange) as a probe and Immunofluorescent stained images of COX IV, HSP70 and GPX4 of 4T1 cells after incubation with different treatments**.** Nuclei were stained with DAPI (marked as blue). The treatments methods of different groups in the figure are as follows: I: PBS − L, II: PBS + L, III: IR-FEP-RGD − L, IV: IR-FEP-RGD + L, V: IR-FEP-RGD-S-S-S-Fc − L, VI: IR-FEP-RGD-S-S-S-Fc + L. + L, and − L are present as 808 nm laser/non-laser irradiation.

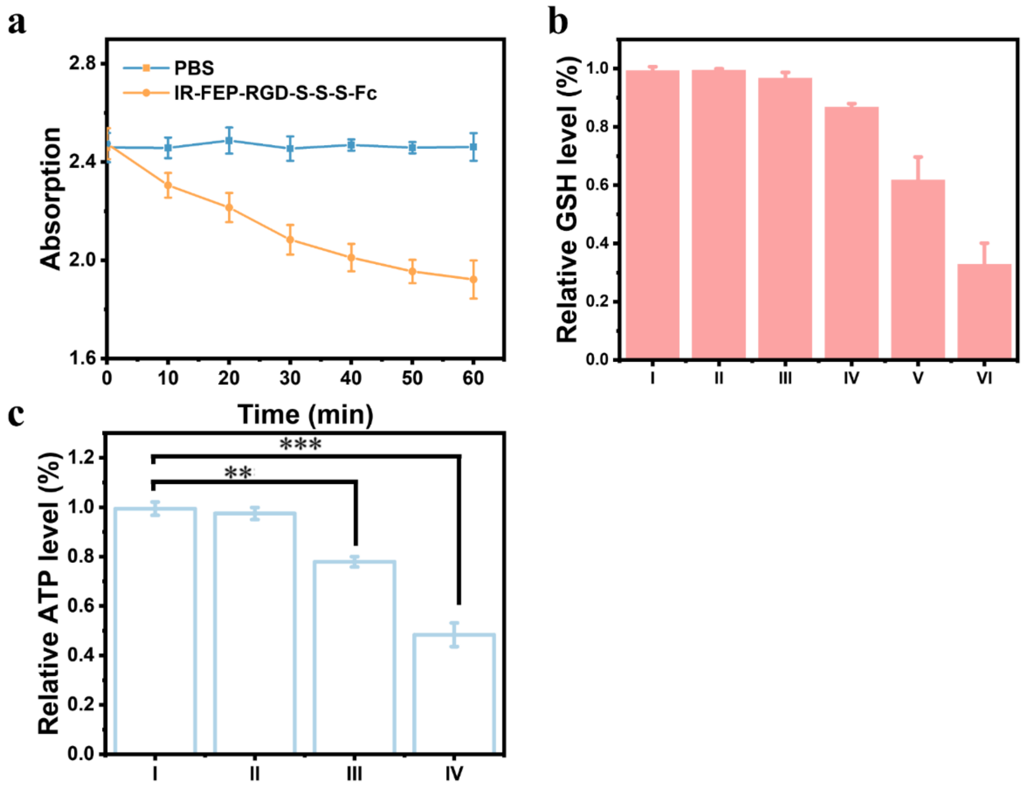

Figure S39. (a) TNB intensity at A_{412} within 60 min in the environment of GSH 10 mM. Error bars: mean ± SD (n = 4). (b) The GSH level of 4T1 cells after incubation with I: PBS − L, II: PBS + L, III: IR-FEP-RGD − L, IV: IR-FEP-RGD + L, V: IR-FEP-RGD-S-S-S-Fc − L, VI: IR-FEP-RGD-S-S-S-Fc + L. Error bars: mean ± SD (n = 4). (c) The ATP levels of the cell after treatment I: PBS − L, II: PBS + L, III: IR-FEP-RGD-S-S-S-Fc − L, IV: IR-FEP-RGD-S-S-S-Fc + L. The significance of the difference of more than two groups was determined *via* ANOVA-LSD *post hoc* test. * $P < 0.05$, ** $P < 0.01$, and *** $P < 0.001$. Error bars: mean ± SD (n = 4).

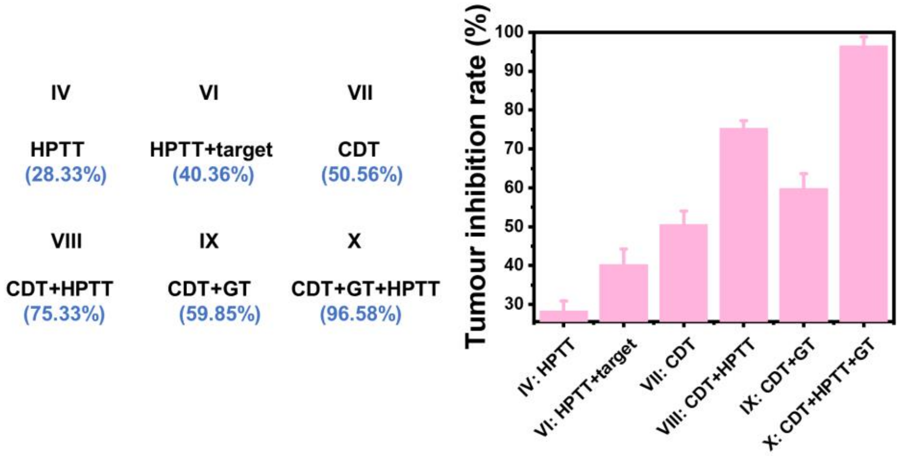

Figure S40. Contribution of different therapeutic modalities concluded from Figure 6b and 6c. Error bars: mean ± SD (n = 4).

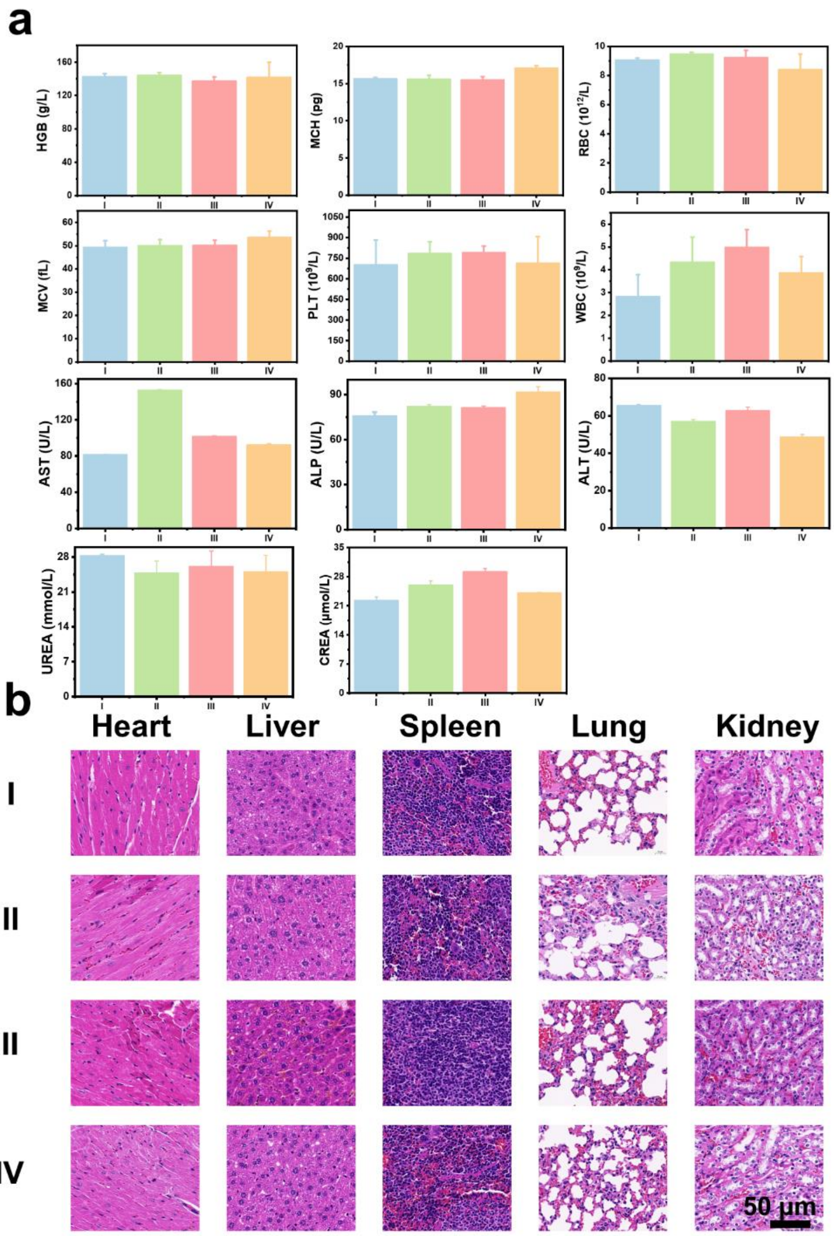

Figure S41. (a) Hematological data of the mice intravenously injected with I: PBS, II: IR-FEP, III: IR-FEP-RGD, IV: IR-FEP-RGD-S-S-S-Fc. The terms are noted as followed: hemoglobin (HGB), mean corpuscular hemoglobin (MCH), mean corpuscular volume (MCV), platelets (PLT), red blood cells (RBC), white blood cells (WBC), alkaline phosphatase (ALP), alanine aminotransferase (ALT), aspartate aminotransferase (AST), carbamide (UREA), and creatinine (CREA). Data are given as mean ± SD (n=3). (b) of sections of the main organs (heart, liver, spleen, lung, kidney) of the five treatment groups: I: PBS, II: IR-FEP, III: IR-FEP-RGD, IV: IR-FEP-RGD-S-S-S-Fc.

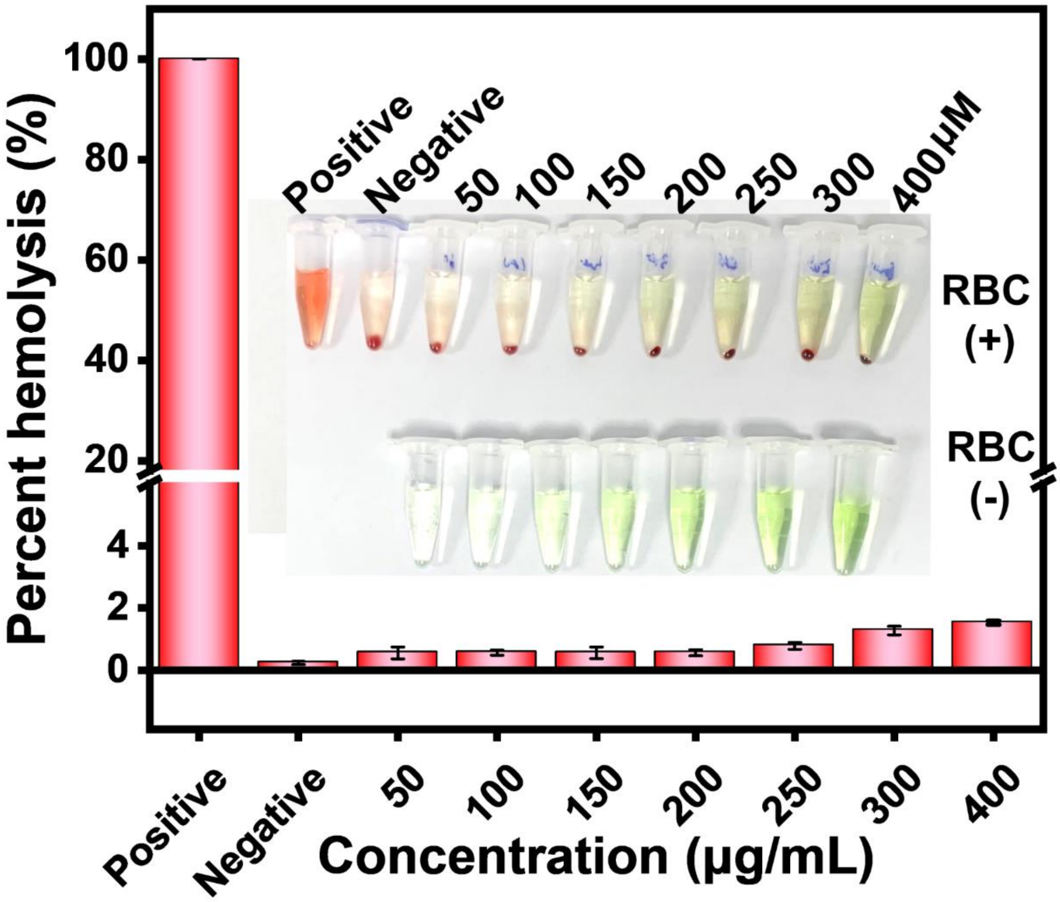

Figure S42. Hemolysis test of IR-FEP-RGD-S-S-S-Fc. Error bars: mean ± SD (n = 4).

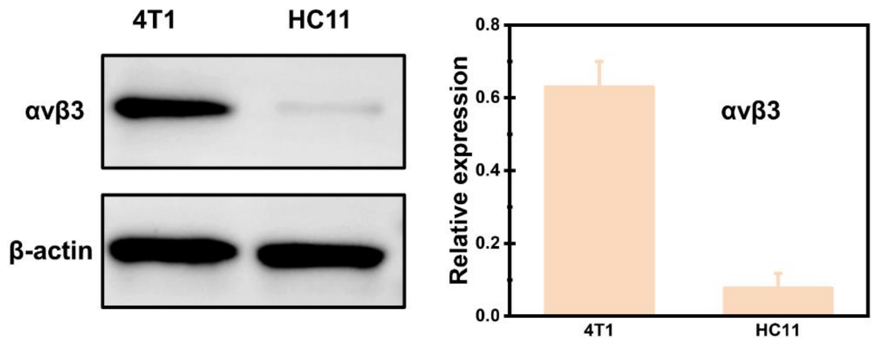

Figure S43. Western blot analysis on the expression levels of αvβ3 in 4T1 and HC11 cells Error bars: mean ± SD (n = 3).

Table S1. Molecule weight determined by SEC-MALLS.

Dye	dn/dc (mL/g)	M_n (Daltons)	M_w (Daltons)	M_w/M_n
IR-FEP	0.040	3100	4000	1.29
IR-FEP-Fc	0.044	4200	4500	1.07
IR-FEP-RGD	0.036	4300	4800	1.12
IR-FEP-RGD-S-S-S-Fc	0.055	5500	5600	1.02

Table S2. Comparison of detection parameters of IR-FEP-RGD-S-S-S-Fc with other GSH fluorescence response platforms.

Samples	Journal/ Publication date	Method	LOD (μM)
CDs-MnO$_2$ NFs	*Sensors and Actuators B: Chemical*/ 2022[6]	fluorometric	0.558
CyA-cRGD	*ACS Applied Materials & Interfaces/ 2018*[7]	fluorometric	0.26
Coumarin-based probe	*Biosensors and Bioelectronics/ 2015*[8]	fluorometric	0.122
Cu NCs	*ACS Applied Materials & Interfaces*/ 2020[9]	fluorometric	0.89
Co-POP	ACS Sustainable Chemistry & Engineering/ 2021[10]	fluorometric	0.71

Reference

[1] Q. Yang, Z. Ma, H. Wang, B. Zhou, S. Zhu, Y. Zhong, J. Wang, H. Wan, A. Antaris, R. Ma, X. Zhang, J. Yang, X. Zhang, H. Sun, W. Liu, Y. Liang, H. Dai, *Adv Mater.* **2017**, *29*, 1605497.

[2] G.-l. Wu, B. Sun, Y. He, X. Tan, Q. Pan, S. Yang, N. Li, M. Wang, P. Wu, F. Liu, H. Xiao, L. Tang, S. Zhu, Q. Yang, *Chem Eng J.* **2023**, *463*, 142372.

[3] Y. Yang, B. Sun, S. Zuo, X. Li, S. Zhou, L. Li, C. Luo, H. Liu, M. Cheng, Y. Wang, S. Wang, Z. He, J. Sun, *Sci Adv.* **2020**, *6*, eabc1275.

[4] S. Diao, G. Hong, J. T. Robinson, L. Jiao, A. L. Antaris, J. Z. Wu, C. L. Choi, H. Dai, *J Am Chem Soc.* **2012**, *134*, 16971-16974.

[5] L. H. Fu, Y. Wan, C. Qi, J. He, C. Li, C. Yang, H. Xu, J. Lin, P. Huang, *Adv Mater.* **2021**, *33*, 2006892.

[6] D. Wang, Y.-t. Meng, Y. Zhang, Q. Wang, W.-j. Lu, S.-m. Shuang, C. Dong, *Sensor Actuat B-Chem.* **2022**, *367*, 132135.

[7] Z. Yuan, L. Gui, J. Zheng, Y. Chen, S. Qu, Y. Shen, F. Wang, M. Er, Y. Gu, H. Chen, *Acs Appl Mater Inter.* **2018**, *10*, 30994-31007.

[8] C. Chen, W. Liu, C. Xu, W. Liu, *Biosens Bioelectron.* **2015**, *71*, 68-74.

[9] C. Liu, Y. Cai, J. Wang, X. Liu, H. Ren, L. Yan, Y. Zhang, S. Yang, J. Guo, A. Liu, *Acs Appl Mater Inter.* **2020**, *12*, 42521-42530.

[10] D. Guo, C. Li, G. Liu, X. Luo, F. Wu, *Acs Sustain Chem Eng.* **2021**, *9*, 5412-5421.
